# Elevated blood pressure and risk of mitral regurgitation: A longitudinal cohort study of 5.5 million United Kingdom adults

**DOI:** 10.1371/journal.pmed.1002404

**Published:** 2017-10-17

**Authors:** Kazem Rahimi, Hamid Mohseni, Catherine M. Otto, Nathalie Conrad, Jenny Tran, Milad Nazarzadeh, Mark Woodward, Terence Dwyer, Stephen MacMahon

**Affiliations:** 1 The George Institute for Global Health, University of Oxford, Oxford, United Kingdom; 2 Oxford University Hospitals NHS Foundation Trust, Oxford, United Kingdom; 3 University of Washington, Seattle, Washington, United States of America; 4 The Collaboration Center of Meta-analysis Research, Sabzevar University of Medical Sciences, Sabzevar, Iran; 5 Torbat Heydariyeh University of Medical Sciences, Torbat Heydariyeh, Iran; 6 The George Institute for Global Health, University of Sydney, Sydney, Australia; 7 Department of Epidemiology, Johns Hopkins University, Baltimore, Maryland, United States of America; Edinburgh University, UNITED KINGDOM

## Abstract

**Background:**

Mitral regurgitation in people without prior cardiac disease is considered a degenerative disease with no established risk factors for its prevention. We aimed to test the hypothesis that elevated systolic blood pressure (SBP) across its usual spectrum is associated with higher risk of mitral regurgitation.

**Methods and findings:**

We used linked electronic health records from the United Kingdom Clinical Practice Research Datalink (CPRD) from 1 January 1990 to 31 December 2015. CPRD covers approximately 7% of the current UK population and is broadly representative of the population by age, sex, and ethnicity. About 5.5 million UK patients with no known cardiovascular or valve disease at baseline were included in this cohort study. We investigated the relationship between blood pressure (BP) and risk of mitral regurgitation using Cox regression models. Our primary exposure variable was SBP and our primary outcome was incident reports of mitral regurgitation, which were identified from hospital discharge reports or primary care records.

Of the 5,553,984 patients in the CPRD that met our inclusion criteria, during the 10-year follow-up period, 28,655 (0.52%) were diagnosed with mitral regurgitation and a further 1,262 (0.02%) were diagnosed with mitral stenosis. SBP was continuously related to the risk of mitral regurgitation with no evidence of a nadir down to 115 mmHg (*p* < 0.001). Each 20 mmHg increment in SBP was associated with a 26% higher risk of mitral regurgitation (hazard ratio [HR] 1.26; CI 1.23, 1.29). The observed association was partially mediated by diseases affecting the left ventricle during follow-up (myocardial infarction [MI], ischaemic heart disease [IHD], cardiomyopathy, and heart failure). However, the percentage of excess risk mediated (PERM) by these proximate causes of secondary mitral regurgitation was only 13% (CI 6.1%, 20%), and accounting for them had little effect on the long-term association between SBP and mitral regurgitation (mediator-adjusted HR 1.22; CI 1.20, 1.25; *p* < 0.001). Associations were similar for each 10 mmHg increment in diastolic blood pressure (DBP) (*p* < 0.001) or each 15 mmHg increment in pulse pressure (PP) (*p* < 0.001). By contrast, there was no association between SBP and risk of mitral stenosis (HR per 20 mmHg higher SBP 1.03; CI 0.93, 1.14; *p* = 0.58). These analyses are based on routinely collected data from health records which may be sensitive to measurement errors, and the observed associations may not be generalizable to less severe and subclinical cases of mitral regurgitation.

**Conclusions:**

Long-term exposure to elevated BP across its whole spectrum is associated with an increased risk of primary and secondary mitral regurgitation. These findings suggest that BP control may be of importance in the prevention of mitral regurgitation.

## Abstract 2

Using data from the UK Clinical Practice Research Datalink, Kazem Rahimi and colleagues examine whether elevated blood pressure is associated with greater risk of mitral regurgitation.

## Author summary

**Why was this study done?:**

- Elevated blood pressure (BP) is a major risk factor for a range of cardiovascular conditions.
- Mitral regurgitation in people without prior cardiac disease is considered a degenerative disease with no established risk factors for its prevention.
- Whether elevated BP is also a risk factor for mitral regurgitation is unknown.

**What did the researchers do and find?:**

- About 5.5 million UK patients with no known cardiovascular or valve disease at baseline were included in this cohort study and were followed up for about 10 years.
- This study shows a clear log-linear association between elevated BP and risk of mitral regurgitation (but not mitral stenosis) with no apparent threshold below or above which the relationship ceased to exist.
- The association was only partially mediated by conditions that are established causes of secondary mitral regurgitation, suggesting that elevated BP has a direct and independent effect on valve degeneration.

**What do these findings mean?:**

- The findings from this study suggest that mitral regurgitation is not an inevitable consequence of ageing and that its risk might be modifiable with established interventions.
- Future studies should establish the causality of the observed associations and test whether BP lowering might reduce the risk of mitral regurgitation.

## Introduction

Mitral regurgitation is the most common valvular heart disorder in high-income countries, and its prevalence increases with age [1,2]. The strong age-dependent prevalence rates suggest that, as a consequence of worldwide ageing and population growth, crude rates of mitral regurgitation are likely to increase further in the coming decades.

Despite substantial progress in our understanding of the pathophysiology of mitral regurgitation and advances in surgical and interventional valve replacement therapies, there are no established preventive strategies [3]. Causes of mitral regurgitation are categorised as “primary” when due to abnormalities of the valve leaflets and chordae or as “secondary” when due to distortion of ventricular shape related to ischaemic heart disease (IHD) or a cardiomyopathy. However, the distinction between these subtypes is not always obvious, and about two-thirds of all mitral regurgitation cases are classified as degenerative [4], implying that they are a natural consequence of ageing with no possibility of altering their course.

Elevated blood pressure (BP) is a strong risk factor for a range of cardiovascular conditions [5]. Given that increased BP correlates with higher left ventricular pressure, and this, in turn, exposes the mitral valve to higher physical stress, it seems plausible that long-term exposure to higher BP could also lead to structural and functional changes of the mitral valve. A cross-sectional analysis of the Framingham study showed a positive association between hypertension and mitral regurgitation [6]. However, to our knowledge, there are no longitudinal studies that have reported associations. We, therefore, aimed to test the hypothesis that elevated systolic blood pressure (SBP) across its usual spectrum is associated with higher risk of mitral regurgitation.

## Methods

### Data source

We used linked electronic health records from the UK Clinical Practice Research Datalink (CPRD) from 1 January 1990 to 31 December 2015. The CPRD contains anonymised patient data from 674 general practices in the UK [7]. It covers approximately 7% of the current UK population and is broadly representative of the population by age, sex, and ethnicity. It partially links primary care records with discharge diagnosis from secondary care (Hospital Episode Statistics) and mortality data from national death registries (Office for National Statistics). The dataset is considered the most comprehensive longitudinal primary care database with serial collection of information relating to diagnosis, treatments, investigations, and outcomes [8] and has been validated for epidemiological research for a range of conditions, including those heavily relying on imaging tests [7,9]. Scientific approval for this study was given by the CPRD Independent Scientific Advisory Committee (ISAC). The CPRD Group has obtained ethics approval from a National Research Ethics Service Committee for all purely observational research using anonymised CPRD data. A separate ethics approval was not required for this work.

### Study population

A total of 6,613,644 patients between 30 and 90 years old with at least 1 BP measurement were identified. Patients entered the study at the date of their earliest BP measurement (baseline) and exited the study at the earliest date of transfer out of the general practice, death, end of the study period, or a record of mitral regurgitation. We further excluded all individuals who at baseline had a prior clinical diagnosis of cardiovascular disease (409,075), mitral valve disease including mitral valve prolapse (14,592), or were prescribed lipid-lowering or antihypertensive medication (196,451). Cardiovascular disease was defined, as previously reported [10,11], using ICD-10 and Read codes for myocardial infarction (MI), IHD (including reports of angina or coronary revascularization), stroke, transient ischaemic attack, heart failure, cardiomyopathy, chronic kidney disease, peripheral arterial disease, atrial fibrillation, or venous thromboembolism. We further excluded individuals with extreme values of baseline SBP with recordings outside the range 50 to 300 mmHg (14,233; 0.08% of all recordings, but their inclusion led to virtually unchanged associations). Patients with less than 1 year follow-up with the general practice (126,358) and patients whose BP measurements were before 1990 (313,633) were also excluded. This led to the selection of 5,553,984 individuals for this study with a median (interquartile interval [IQI]) follow-up duration of 10 (4.7, 17) years.

### Outcomes and exposures

Our primary outcome was incident reports of mitral regurgitation, which were identified from hospital discharge reports, death registers, or primary care records using the diagnostic codes shown in S1 Table. We excluded diagnostic codes which classified mitral valve disease as congenital or when mixed mitral valve disease was reported with no clear indication of the dominant condition. We did not have complete information on echocardiography for all patients. However, previous validation studies based on electronic health records have shown that the majority of clinically recorded valve disease codes are based on echocardiographic assessments and have a high positive predictive value for the valve diagnosis (>85%) [2]. They further show that recorded cases typically represent moderate to severe severity ratings that are clinically relevant [2] (whereas epidemiological studies with population screening tend to identify a larger number of patients with mild and asymptomatic diseases [12]). For more direct validation of valve disease and its severity in our study, we performed 3 subgroup analyses (see Statistical analysis). In addition, as a negative control secondary outcome, we chose incident reports of mitral stenosis, expecting no association with BP, since mitral stenosis without regurgitation is nearly always due to rheumatic valve disease. Finally, to test the validity of database and methods for measuring and modelling exposure variables and covariates, we chose incident stroke as a positive control outcome and report BP associations with stroke.

BP measurements were identified from the CPRD database as measurements with an entity type 1 (the entity type corresponding to a BP measurement). We defined SBP as our primary exposure because SBP has been shown to have the strongest predictive ability among most other measures of BP for most cardiovascular outcomes [13,14] and because it is a better indicator of afterload than diastolic blood pressure (DBP) and hence could play a stronger role in the pathophysiology of mitral regurgitation. However, in additional prespecified analyses, we chose DBP and pulse pressure (PP; defined as SBP–DBP) as alternative exposure variables to investigate any differential effect of BP indices on risk and to enable comparison of our findings with epidemiological studies of BP associations with other outcomes [13–15].

### Statistical analysis

In consideration of measurement error surrounding a single BP measurement and other time-dependent fluctuations of BP (in particular in the context of routinely recorded BP by doctors and nurses), we used multiple BP measurements to calculate ‘usual’ values that are corrected for regression dilution [16]. As previously reported [10,11,17], we used generalised estimating equations to regress serial BP measurements within the median follow-up (mean of 6.7 measurements per patient) on the baseline BP. Usual SBP, DBP, and PP were analysed as continuous variables with 20 mmHg, 10 mmHg, and 15 mmHg increments, respectively. SBP was further analysed as a categorical variable (≤120 mmHg, 121 mmHg to 140 mmHg, 141 mmHg to 160 mmHg, and ≥160 mmHg), modelled as a continuous variable with 1 mmHg increments, and plotted using Cox regression with restricted cubic spline with 4 knots. We determined the association between SBP (and other BP indices) and each outcome using Cox regression, with hazard ratios (HRs) displayed with floating absolute risks, which do not require the selection of an arbitrary baseline group for display of confidence intervals [18]. We tested the proportional hazards assumption of Cox models using log cumulative hazards plots for SBP categories and found no evidence of lack of proportional hazards (S1 Fig). Our primary analyses were adjusted for baseline sex, age, body mass index (BMI, kg/m^2^), smoking status (no, previous, yes), cholesterol (total, low-density lipoprotein [LDL], high-density lipoprotein [HDL]), and year of the initial BP measurement (as a categorical variable [1990 to 1994, 1995 to 1999, 2000 to 2004, 2005 to 2009, and 2010 to 2013]) to control for potential cohort effects, and stratified by general practice. For BMI, smoking, and lipid levels, we utilised the most recent measurement within 2 years of the baseline SBP measurement (index encounter with the general practice when SBP was recorded). If no measurement was available within this timeframe, we classified the covariate as missing. We addressed missing data using multiple imputation by expectation-maximisation with bootstrapping [19], generating 5 imputations [20].

To investigate the extent to which the potential association between BP and mitral regurgitation is mediated by incident MI, IHD, heart failure, or cardiomyopathy, which are established causes of secondary mitral regurgitation, we performed time-varying adjustments for these events during the follow-up. The HR from this confounder- and mediator-adjusted model provides an estimation of association between BP and primary mitral regurgitation. To determine what proportion of the association between usual BP and risk of mitral regurgitation was mediated by these events, we then calculated the percentage of excess risk mediated (PERM) using the formula: PERM = (HR_confounder-adjusted_ − HR_confounder- and mediator-adjusted_)/(HR_confounder-adjusted_). We estimated confidence intervals through percentile bootstrapping based on 1,000 runs.

We performed subgroup analyses and report interactions by age, sex, and BMI groups. We also compared the predictive value of PP, mean arterial pressure, and mid arterial pressure on the risk of mitral regurgitation using Harrell’s c-statistic [21]. Mean arterial pressure was defined as 2/3 DBP + 1/3 SBP and mid arterial pressure as 1/2 SBP + 1/2 DBP.

To investigate the validity of our outcome variables and the potential effect of valve severity on outcomes, we performed the following 3 sensitivity analyses. First, we extracted information on echocardiograms within 14 days prior to the recorded diagnosis of mitral regurgitation and stratified analyses by echocardiographically supported diagnoses of mitral regurgitation versus other reports with no recorded information on diagnostic tools. Second, we stratified our analyses according to whether or not there was a definitive report of valve replacement therapy (i.e., surgical or interventional valve replacement or repair) to assess whether observations might have been diluted due to inclusion of mild or asymptomatic cases. Third, we stratified our analyses by initial source of case reporting (hospital discharge versus outpatient or primary care), assuming hospital diagnosis cases to be more severe and more likely to be verified by specialists. Additional sensitivity and validation analyses are described in S1 Text.

**Fig 1:**
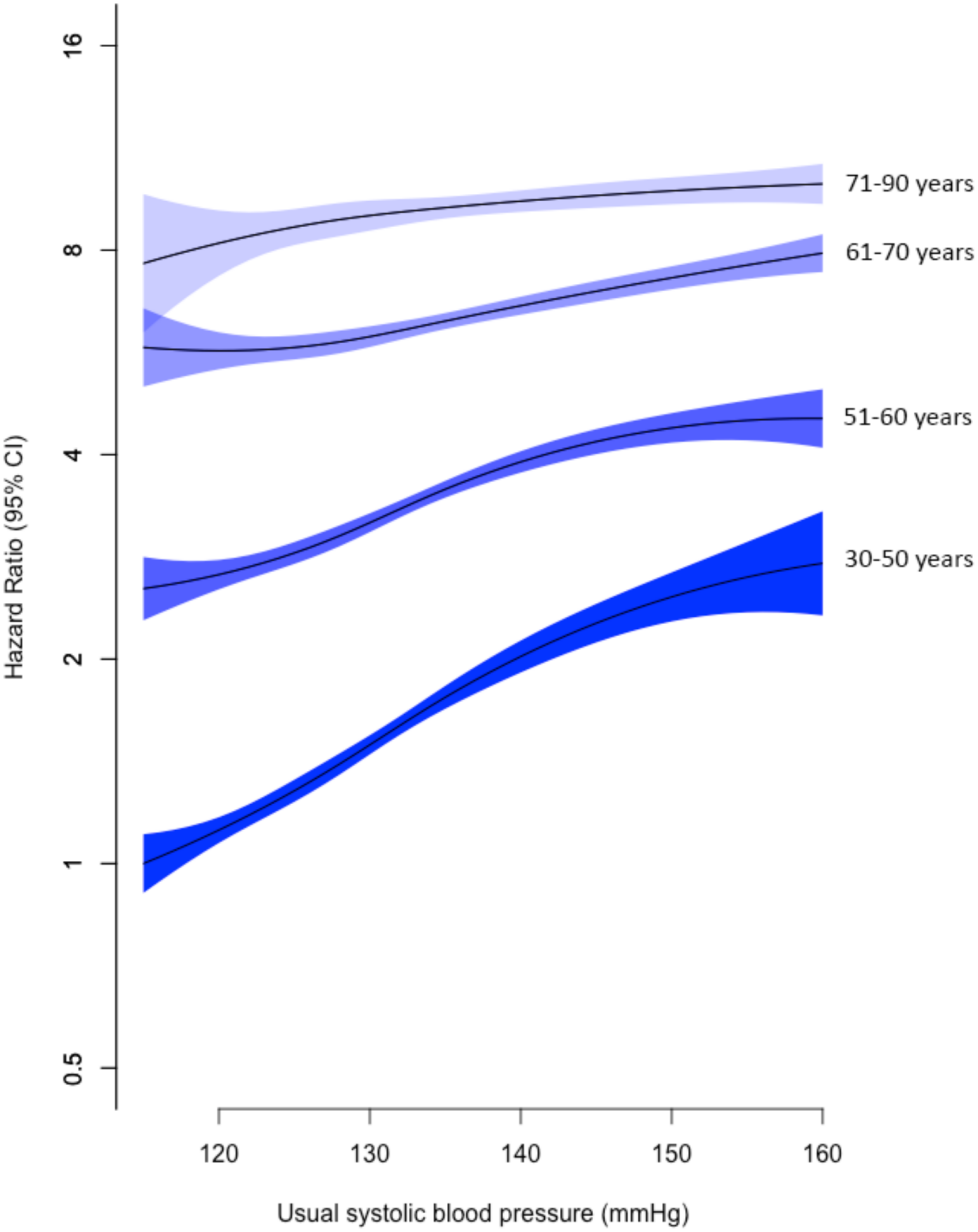
Adjusted age-specific HRs of SBP for mitral regurgitation. Age bands refer to age at baseline. Adjustments were for sex, BMI, smoking, year of BP measurement, total cholesterol, LDL cholesterol, and HDL cholesterol. Confidence intervals are displayed as floating absolute risks. HRs and 95% CI are displayed for 1 mmHg increments in SBP and plotted using Cox regression with restricted cubic spline with 4 knots, relative to the reference category (individuals aged 30–50 years with usual SBP 115–120 mmHg). Abbreviations: BMI, body mass index; BP, blood pressure; HDL, high-density lipoprotein; HR, hazard ratio; LDL, low-density lipoprotein; SBP, systolic blood pressure.

Study findings are reported in accordance with the reporting of studies conducted using Observational Routinely-collected health Data (RECORD) Statement [22]. There was no specific analysis plan for the current study, but the main analysis followed a general analysis plan for investigation of associations between BP and several outcomes including valvular heart disease (S2 Text and earlier reports [10,11,17,23] describing the general analysis plan). Deviations from the general protocol were largely informed by the clinical differences between outcomes and reviewer comments. More specifically, for this paper, we introduced a mediation analysis to assess the mediator-corrected associations and several sensitivity analyses to validate our findings (see above and S1 Text). In addition, the restricted cubic spline Cox regression (Fig 1) and the further division of age groups in Fig 3 were specific requests by reviewers of this journal. Statistical analyses were performed in R, version 3.3 (R Foundation for Statistical Computing, Vienna, Austria).

## Results

Of the 5,553,984 patients in the CPRD that met our inclusion criteria, 28,655 (0.52%) were diagnosed with mitral regurgitation during follow-up and a further 1,262 (0.02%) were diagnosed with mitral stenosis. Patient characteristics by usual SBP categories are shown in Table 1.

**Table 1:** Baseline characteristics and outcome rates by categories of usual SBP.

	<121 mmHg *n* = 1,362,861	121–140 mmHg *n* = 3,669,483	141–160 mmHg *n* = 486,060	>160 mmHg *n* = 35,580	Total *n* = 5,553,984
**Mitral valve disease, *n* (%)**					
Mitral regurgitation	2,621 (0.19)	18,350 (0.5)	7,047 (1.4)	637 (1.8)	28,655 (0.52)
Mitral stenosis	143 (0.01)	796 (0.025)	298 (0.061)	25 (0.07)	1,262 (0.023)
**Age categories, years, *n* (%)**					
30–50	1,264,938 (93)	2,730,650 (74)	133,965 (28)	4,295 (12)	4,133,848 (74)
51–60	65,106 (4.8)	477,074 (13)	111,379 (23)	6,872 (19)	660,431 (12)
61–70	21,510 (1.6)	280,910 (7.7)	119,808 (25)	10,195 (29)	432,423 (7.8)
71–90	11,307 (0.83)	180,849 (4.9)	120,908 (25)	14,218 (40)	327,282 (5.9)
Age, median (IQI)	32 (30, 40)	39 (33, 52)	60 (49, 70)	67 (57, 74)	39 (33, 52)
**Sex, *n* (%)**					
Female	969,470 (71)	1,809,216 (49)	240,430 (49)	20,899 (59)	3,040,015 (55)
**BMI (kg/m^2^) categories, *n* (%)**					
≤25	762,328 (72)	1,376,738 (49)	110,744 (32)	7,548 (32)	2,257,358 (54)
26–30	228,736 (22)	940,840 (34)	134,845 (40)	9,113 (39)	1,313,534 (31)
31–35	53,260 (5.0)	327,748 (12)	63,301 (19)	4,467 (19)	448,776 (11)
>35	18,183 (1.7)	142,824 (5.1)	32,416 (9.5)	2,439 (10)	195,862 (4.6)
Missing, %	30	24	30	34	24
BMI, median (IQI)	23 (21, 26)	25 (23, 28)	27 (24, 31)	27 (24, 31)	25 (22, 28)
**Smoking history, *n* (%)**					
Never smoked	701,039 (58)	1,815,237 (57)	223,881 (57)	16,304 (58)	2,756,461 (57)
Ex-smoker	125,566 (10)	431,724 (14)	71,424 (18)	5,261 (19)	633,975 (13)
Current smoker	373,176 (31)	939,597 (29)	96,528 (25)	6,445 (23)	1,415,746 (29)
Missing, %	12	13	19	21	13
**Cholesterol (mmol/L), median (IQI)**					
Total	5.0 (4.3, 5.7)	5.3 (4.6, 6.1)	5.6 (4.8, 6.3)	5.6 (4.8, 6.4)	5.3 (4.6, 6.1)
Total missing, %	92	84	71	63	85
LDL	3.0 (2.4, 3.7)	3.2 (2.6, 3.9)	3.3 (2.6, 4.0)	3.3 (2.6, 4.1)	3.2 (2.6, 3.9)
LDL missing, %	96	91	83	81	93
HDL	1.4 (1.2, 1.7)	1.3 (1.1, 1.6)	1.3 (1.1, 1.6)	1.39 (1.1, 1.7)	1.3 (1.1, 1.6)
HDL missing, %	95	90	79	76	92
**Intermediary conditions, *n* (%)**	22,279 (1.6)	197,899 (5.4)	87,223 (18)	8,814 (25)	316,215 (5.7)
**Follow-up (year), median (IQI)**	9.2 (4.1, 16)	10.0 (4.7, 17)	12 (6.6, 17)	11 (6.7, 16)	10 (4.7, 17)

Intermediary conditions: IHD, MI, heart failure or cardiomyopathy during follow-up.

Abbreviations: BMI, body mass index; HDL, high-density lipoprotein; IHD, ischaemic heart disease; IQI, interquartile interval; LDL, low-density lipoprotein; MI, myocardial infarction; SBP, systolic blood pressure.

Age-specific analyses showed a continuous and approximately log-linear adjusted relationship between usual SBP and risk of mitral regurgitation throughout the SBP range with no evidence of a threshold below or above which the associations were different (Fig 1). As expected, the HRs for a given difference in usual SBP are smaller in older age groups (as indicated by the slope of the age-specific associations). However, the absolute risk of developing mitral regurgitation increases substantially with age, leading to a substantial net contribution of elevated SBP on risk of mitral regurgitation even in the older age groups (Fig 1).

**Fig 2:**
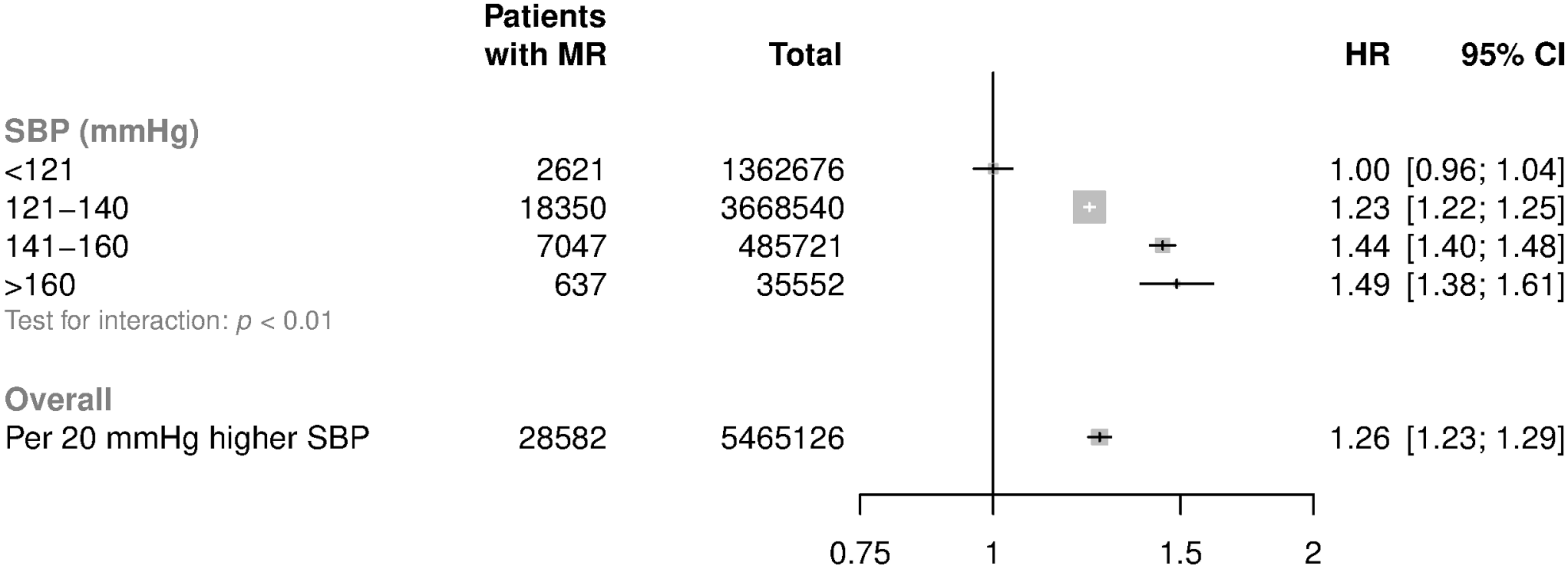
HRs for mitral regurgitation by categories of usual SBP. HRs and 95% CI are displayed using floating absolute risk and are corrected for regression dilution. Models are adjusted for age, sex, BMI, smoking, calendar year, total cholesterol, LDL cholesterol, and HDL cholesterol. Abbreviations: BMI, body mass index; HDL, high-density lipoprotein; HR, hazard ratio; LDL, low-density lipoprotein; MR, mitral regurgitation; SBP, systolic blood pressure.

When usual SBP was analysed as a continuous variable, each 20 mmHg increment in usual SBP was associated with a 26% higher risk of mitral regurgitation (adjusted HR 1.26; CI 1.23, 1.29) (Fig 2). Compared with the reference category of usual SBP ≤ 120 mmHg, patients with usual SBP ≥ 161 mmHg were 1.5 times more likely to be diagnosed with mitral regurgitation (HR 1.49; CI 1.38, 1.61) (Fig 2).

**Fig 3:**
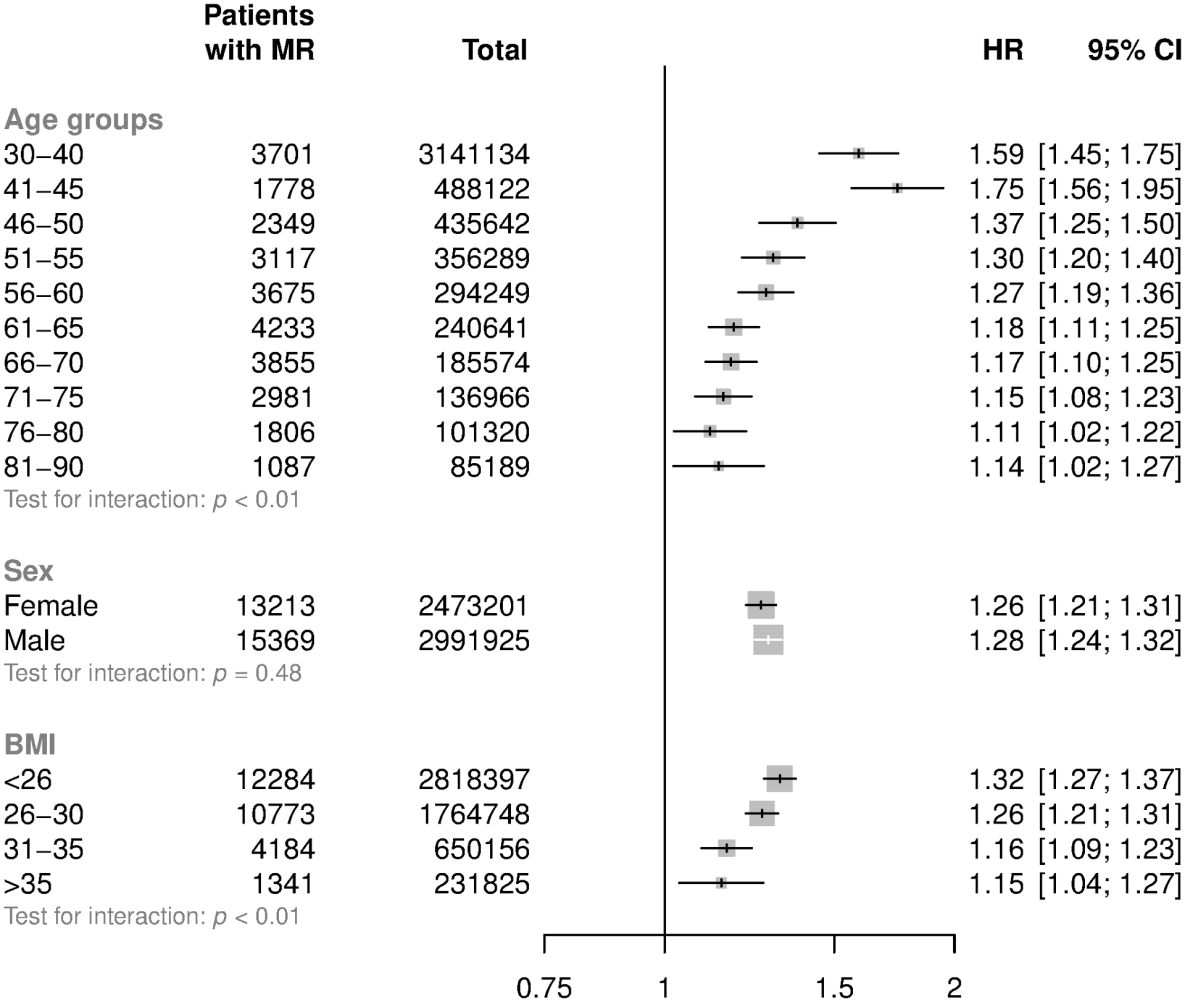
HRs for mitral regurgitation per 20 mmHg higher usual SBP by age, sex, and BMI categories. HRs and 95% CI are displayed using floating absolute risks and are corrected for regression dilution. Models are adjusted for age (per year), sex, BMI, smoking, calendar year, total cholesterol, LDL cholesterol, and HDL cholesterol. Abbreviations: BMI, body mass index; HDL, high-density lipoprotein; HR, hazard ratio; LDL, low-density lipoprotein; MR, mitral regurgitation; SBP, systolic blood pressure.

**Fig 4:**
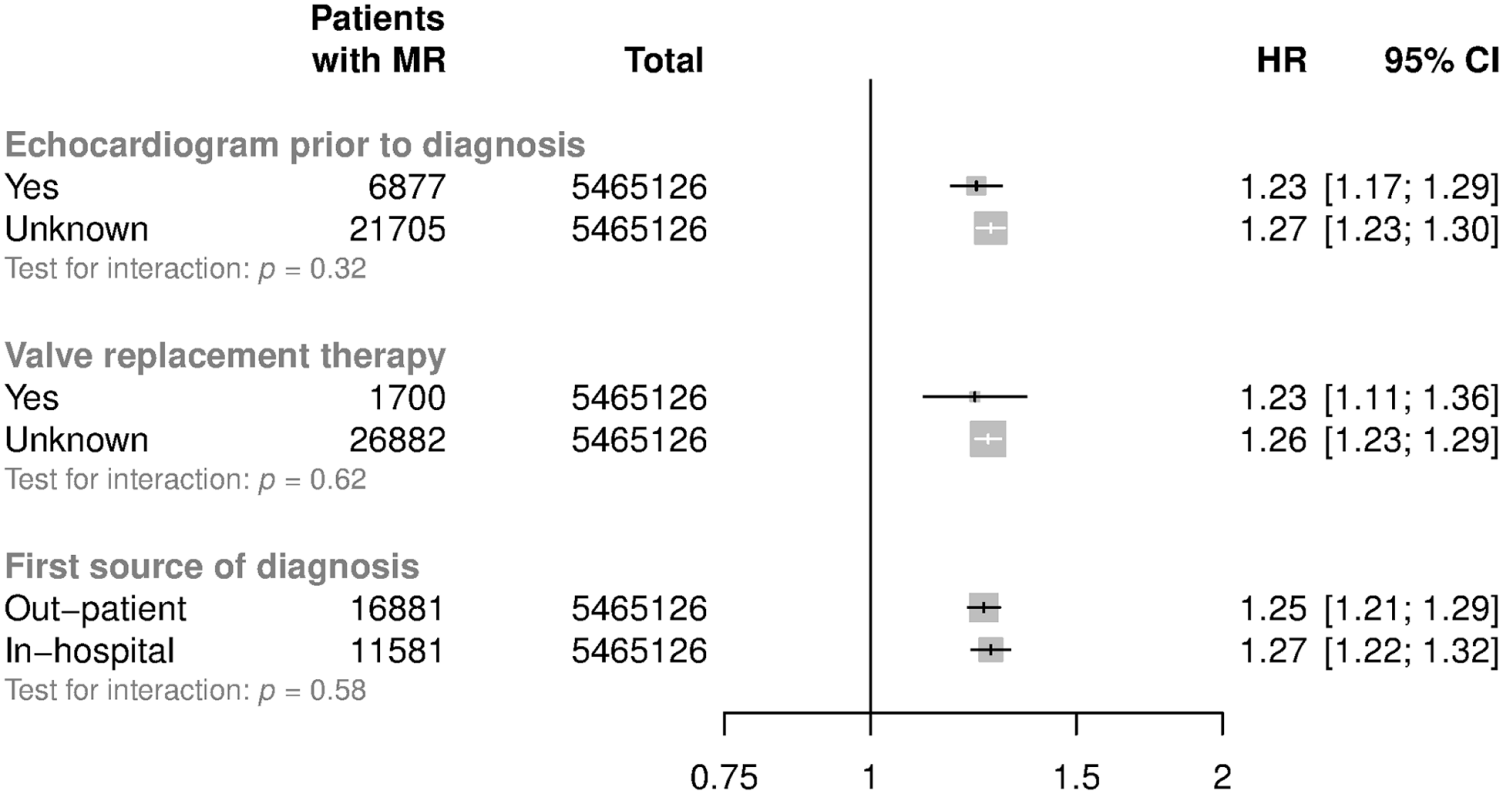
Subgroup analyses for mitral regurgitation per 20 mmHg higher usual SBP. Models are adjusted for sex, BMI, smoking, calendar year, total cholesterol, LDL cholesterol, and HDL cholesterol. Valve replacement therapy refers to mitral valve repair or replacement. Confidence intervals are displayed as floating absolute risks. Abbreviations: BMI, body mass index; HDL, high-density lipoprotein; HR, hazard ratio; LDL, low-density lipoprotein; MR, mitral regurgitation; SBP, systolic blood pressure.

Adjusted HRs among subgroups are shown in Fig 3. HRs attenuated with increasing age (*p* for trend < 0.001). For patients aged less than 50 years at baseline, each 20 mmHg increment in SBP was associated with a 54% higher risk of mitral regurgitation (HR 1.54; CI 1.46, 1.63), whereas in patients aged 71–90 the risk was higher by 13% (HR 1.13; CI 1.08, 1.19) (Fig 3). Proportional differences were broadly similar between men and women. There was evidence that the associations between usual SBP and mitral regurgitation differed by baseline BMI, with weaker associations observed among those with higher BMI (*p* for interaction < 0.01, Fig 3, *p* for trend = 0.44). Associations were similar when echocardiographically supported reports were compared with reports without evidence of an echocardiogram within 14 days before the valve diagnosis, when hospital discharge reports were compared with outpatient diagnoses recorded by general practitioners, or when definitive reports of valve replacement were compared with patients with no clear reports of valve replacement (Fig 4).

Associations of PP and DBP with mitral regurgitation were broadly consistent with those for SBP: each 15 mmHg higher usual PP was associated with a 23% greater risk of mitral regurgitation (HR 1.23; CI 1.20, 1.27), and each 10 mmHg higher usual DBP was associated with a 24% greater risk of mitral regurgitation (HR 1.24; CI 1.20, 1.28). Similar to SBP, these associations differed by age group, with stronger associations observed among the younger groups (*p* for trend < 0.001 for both DBP and PP, S2 Fig). In comparative analyses, SBP showed the strongest, and DBP the weakest, overall predictive power among the BP indices investigated (S2 Table). Age-stratified analyses were broadly consistent with the overall results (S2 Table).

IHD, MI, heart failure, or cardiomyopathy during follow-up were strongly associated with mitral regurgitation (HR 2.94; CI 2.86, 3.03). Adjusting for their effect provides an estimate of the association between BP and mitral regurgitation without a mediating effect of left ventricular disease as the more proximal causes of secondary mitral regurgitation. This adjustment led to a mild attenuation of associations between SBP and mitral regurgitation (from confounder-adjusted HR 1.26; CI 1.23, 1.29 to confounder- and mediator-adjusted HR 1.22; CI 1.20, 1.25).

The percentage of excess risk attributable to these intermediary outcomes was 13% (CI 6.1% to 20%), suggesting that only 13% of the overall association across all primary and secondary mitral regurgitation reports is mediated by left ventricular disease prior to a diagnosis of mitral regurgitation. The potential effect of over-adjustment assessed by a progressive adjustment of covariates, in which consecutively adjusting for each covariate had little effect on the estimated HRs per 20 mmHg higher SBP for mitral regurgitation (S3 Fig).

In our negative control analysis, there was no association between SBP and risk of mitral stenosis (HR per 20 mmHg higher SBP 1.03; CI 0.93, 1.14) (S4–S6 Figs). Also supporting our main findings, our positive control analysis with stroke as the outcome did not show any evidence of bias towards extreme: overall each 20 mmHg increment in SBP was associated with a 34% higher risk of stroke (HR 1.34; CI 1.33, 1.35) across all age groups or a 43% higher risk of stroke (HR 1.43; CI 1.40, 1.47) among the patients aged between 50 and 60 years old. Additional sensitivity analyses produced very similar results to our main analyses (S1 Text).

## Discussion

In this large-scale cohort of UK adults involving approximately 30,000 new cases of mitral regurgitation with 10 years of follow-up, we show that exposure to elevated BP was continuously associated with an increased risk of mitral regurgitation with no apparent threshold at which the association ceased to exist. More specifically, every 20 mmHg increment in usual SBP was associated with a 26% higher risk of mitral regurgitation during follow-up. Associations differed by baseline features of patients, with stronger associations observed in younger and leaner patient groups.

Mitral regurgitation is a heterogeneous condition that can be caused by primary structural abnormalities of the valve apparatus (primary mitral regurgitation) or diseases of the left ventricle leading to incomplete closure of a structurally normal valve (secondary mitral regurgitation). The main diseases of the left ventricle that are commonly considered as more proximate causes of secondary mitral regurgitation are MI, IHD, heart failure, and cardiomyopathy. Because elevated BP is a known risk factor for these conditions, it is possible that the observed association between BP and mitral regurgitation is entirely or largely a reflection of such indirect effects. However, accounting for the possible intermediary effect of diseases of the left ventricle had little impact on the observed associations between elevated BP and risk of mitral regurgitation. In confounder- and mediator-adjusted models, which in essence provide an estimation of the association between BP and primary mitral regurgitation, the overall HR was only slightly attenuated and only 13% of the excess risk could be attributed to the intermediary outcomes. This indicates that 87% of the effect of BP on mitral regurgitation was independent of diseases of the left ventricle that occurred during follow-up, suggesting that BP may exert its main effect on the mitral valve directly or via mechanisms unrelated to left ventricular dilatation or dysfunction. However, our mediation analysis was relatively crude and alternative causal inference modelling approaches might be better suited to disentangle the effect of mediators and confounders during follow-up.

Our study was not designed to investigate the underlying mechanisms of the observed associations between BP and mitral regurgitation, and we can only speculate about possible pathophysiological pathways. Physiologically, the mitral valve is exposed to considerable mechanical force in systole reflecting rapid closure of the valve at the start of systole and subsequent increased pressure difference between the left ventricle and atrium throughout systole. High BP is also associated with elevated left atrial diastolic pressures, which during diastole leads to a higher mitral valve opening pressure. This might alter blood flow patterns, resulting in abnormal shear stress on the atrial surface of the valve leaflets, as well as abnormal shear stress on the ventricular side of the leaflets when the valve is closed. Therefore, it seems plausible that the mechanical stress caused by the elevated BP will lead to gradual structural changes of the valve apparatus. Future studies could explore these and alternative mechanisms further. Such studies, ideally when repeated cardiac imaging is available, might also be able to investigate whether the observed associations differ by presence or type of underlying valve pathologies, such as Barlow disease or mitral valve prolapse [24].

We are not aware of any prior longitudinal studies that have investigated the association between BP and risk of mitral regurgitation. A cross-sectional study based on the Framingham cohort involved 2,881 participants and showed that those with hypertension had a 1.6 times higher risk of concomitant mitral regurgitation (CI 1.2, 2.0) [6]. Further evidence in support for our findings comes from studies that have reported an association between elevated BP and risk of mitral annulus calcification, which is related to degenerative mitral regurgitation [25]. Our large-scale cohort study extends these prior findings and shows that exposure to elevated BP is significantly associated with an increased the risk of mitral regurgitation.

Our analyses are based on routinely collected data from linked electronic health records, which might be more prone to measurement errors than prospectively designed research datasets. However, measurement errors in our exposure variable are unlikely to be biased, given that BP measurements were often recorded several years before the occurrence of outcomes and many of them were within the conventionally ‘normal’ range. This is supported by the lack of association between BP and mitral stenosis in our study (although this does not exclude a significant association in certain groups of patients with mitral stenosis, such as elderly in whom mitral stenosis may be due to mitral annulus calcification). The confirmation of the association between BP and stroke in our validation analysis further supports the suitability of our database and exposure variable for investigation of valve disease [13,14]. To reduce the impact of inevitable random measurement error in our exposure variable, we used multiple BP measurements and corrected baseline values for regression dilution. We did not adjust for anti-hypertensive use during follow-up but this and any other residual uncontrolled measurement error in BP measurements would only be expected to have biased our estimates towards the null and reduced statistical power [16,26]. In addition, our regression dilution correction method for BP measurements indirectly takes account of the effect of anti-hypertensive treatment during follow-up, consistent with prior research that has shown that additional control for anti-hypertensive use has little impact on the regression dilution ratios [27]. As for our outcome variable mitral regurgitation, it is well known that studies based on clinically reported events are more likely to only capture functionally relevant and symptomatic disease states and, for this reason and because of incomplete record linkage, will underestimate rates compared to epidemiological studies that screen the entire population for subclinical and clinical disease. Thus, the observed associations may not be generalizable to less severe and subclinical cases of mitral regurgitation. Within the setting of clinical mitral regurgitation, any random measurement error in diagnostic coding or missingness as a result of incomplete case ascertainment would be expected to make estimates less precise and is unlikely to have any material impact on our overall findings [16]. In addition, our findings were robust to several alternative assumptions, including a comparison of in-patient and out-patient diagnoses and cases with a definitive report of echocardiogram versus others, suggesting that residual confounding is unlikely to have played a substantial role. Nonetheless, as with most observational studies, our study in isolation may not establish causation beyond doubt. New trials with mitral valve regurgitation as an outcome are needed to answer this question reliably. Alternatively or in addition, individual patient data meta-analysis of unreported valvular outcomes from previously published trials or Mendelian randomisation studies might provide additional insights into the potential causal role of BP on mitral regurgitation.

In conclusion, our large-scale prospective study suggests that long-term exposure to elevated BP across its whole spectrum is associated with an increased risk of primary and secondary mitral regurgitation. These findings suggest that BP control may be of importance in the prevention of mitral regurgitation.

## Supporting information

S1 Text Additional sensitivity analyses. (DOCX)

S2 Text General study protocol. (DOC)

S1 Fig Log cumulative hazards plots for SBP relating to mitral regurgitation. Abbreviation: SBP, systolic blood pressure.
(DOCX)

S2 Fig HRs for mitral regurgitation per 10 mmHg higher usual DBP or per 15 mmHg higher usual PP, by age categories. Abbreviations: DBP, diastolic blood pressure; HR, hazard ratio; PP, pulse pressure.
(DOCX)

S3 Fig HRs per 20 mmHg higher SBP for mitral regurgitation, with progressive adjustment for age, sex, calendar year, BMI, smoking, and baseline LDL cholesterol, HDL cholesteral, and total cholesterol, and stratified by practice level. Abbreviations: BMI, body mass index; HDL, high-density lipoprotein; HR, hazard ratio; LDL, low-density lipoprotein; SBP, systolic blood pressure.
(DOCX)

S4 Fig Adjusted age-specific HRs of SBP for mitral stenosis. Abbreviations: HR, hazard ratio; SBP, systolic blood pressure.
(DOCX)

S5 Fig HRs for mitral stenosis by categories of usual SBP. Abbreviations: HR, hazard ratio; SBP, systolic blood pressure.
(DOCX)

S6 Fig HRs per 20 mmHg higher SBP for mitral stenosis, with progressive adjustment for age, sex, calendar year, BMI, smoking, and baseline LDL cholesterol, HDL cholesterol, and total cholesterol, and stratified by practice level. Abbreviations: BMI, body mass index; HDL, high-density lipoprotein; HR, hazard ratio; LDL, low-density lipoprotein; SBP, systolic blood pressure.
(DOCX)

S1 Table Read and ICD-10 codes for mitral valve disease. (DOCX)

S2 Table Relative ability of different BP indices to independently predict mitral regurgitation, by age categories and overall. Abbreviation: BP, blood pressure.
(DOCX)

S1 RECORD Checklist RECORD checklist. (DOCX)

## Abbreviations

BMI: body mass index
BP: blood pressure
CPRD: Clinical Practice Research Datalink
DBP: diastolic blood pressure
HDL: high-density lipoprotein
HR: hazard ratio
IHD: ischaemic heart disease
IQI: interquartile interval
LDL: low-density lipoprotein
MI: myocardial infarction
PERM: percentage of excess risk mediated
PP: pulse pressure
SBP: systolic blood pressure
