# Supplementary material for: Elevated blood pressure and risk of mitral regurgitation: A longitudinal cohort study of 5.5 million United Kingdom adults
Source: PLoS Med. 2017 Oct 17;14(10):e1002404. doi: 10.1371/journal.pmed.1002404 (PMC5644976; doi:10.1371/journal.pmed.1002404)

### **S1 Text.** Additional sensitivity analyses

Methods:

We performed three additional sensitivity analyses to test the validity of our findings. First, we repeated the primary analyses and excluded the first two years of follow-up and then the first three years of follow-up, to account for potential reverse causality (i.e., incipient disease-causing changes in SBP as opposed to the other way around). Second, we excluded patients with any diagnostic reports of rheumatic valve disease. Third, we excluded total cholesterol, LDL and HDL as covariates from the primary analyses due to their high proportion of missing values and assessed the impact of progressive covariate adjustment. Forth, we increased the number of imputations from 5 to 20 to test the robustness of our findings.

Results:

These sensitivity analyses produced very similar results to our main analyses (per each 20 mmHg SBP difference; HR 1.26; CI 1.23, 1.29; Figure 2): First, excluding patients with a report of mitral regurgitation during the first two years of follow-up had no effect (HR 1.26; CI 1.22; 1.29); and excluding the first three years had minimal impact on associations (HR 1.26; CI 1.23, 1.29). Second, excluding diagnostic mention of rheumatic valve disease had no impact on the estimates (HR 1.26, CI 1.23, 1.29). Third, excluding total cholesterol, LDL and HDL cholesterol from the model did not have any impact on associations either (HR 1.26, CI 1.23, 1.29). This was further supported by our analysis of progressive adjustment for covariates, which showed that after adjustment for age, additional adjustments for covariates had little impact on estimates (S3 Fig). Forth, 20 sets of imputations also did not have any impact on the observed associations (see below Fig).

### **Fig.** Hazard ratios for mitral regurgitation per 20 mmHg higher usual systolic blood pressure after 20 sets of imputations for missing co-variates, by age categories.


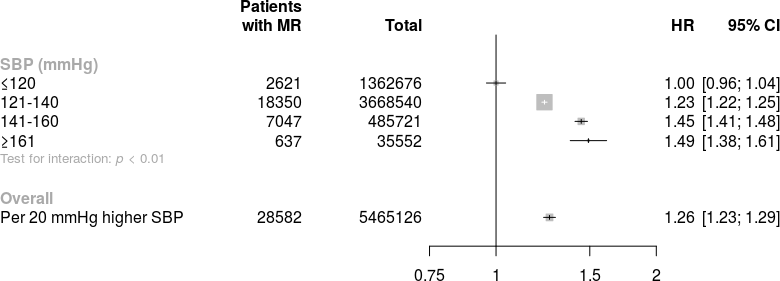

Supplement: S1 Text — (DOCX) [file pmed.1002404.s001.docx]
