# Supplementary material for: Elevated blood pressure and risk of mitral regurgitation: A longitudinal cohort study of 5.5 million United Kingdom adults
Source: PLoS Med. 2017 Oct 17;14(10):e1002404. doi: 10.1371/journal.pmed.1002404 (PMC5644976; doi:10.1371/journal.pmed.1002404)
Supplement: S2 Text — (DOC) [file pmed.1002404.s002.doc]

**The association between blood pressure and cardiovascular events: an analysis of CPRD.**

*Lay summary*

High blood pressure is the leading risk factor for death and disability worldwide.[1] A study that examined the association between blood pressure and cardiovascular disease in over 900,000 individuals found a log-linear relationship between the incidence of cardiovascular mortality and usual blood pressure;[2] for every 10 mm Hg difference in resting systolic blood pressure, there was an associated 25% difference in the incidence of coronary heart disease (CHD) mortality and a 36% difference in the incidence of stroke mortality.[2] In this analysis, there was no threshold beyond which a lower blood pressure was not associated with a lower risk of vascular mortality. However, this study was not able to examine blood pressure levels below 115 mm Hg systolic. Additionally, other observational analyses have contradicted these results and concluded that lower blood pressures in the range of 120 or even higher can be harmful.[3] Consequently, there is controversy over what diastolic and systolic blood pressure levels are associated with the lowest risk of vascular mortality and whether the levels differ by prior cardiovascular disease or different types of outcomes (e.g. stroke vs. myocardial infarction). In order to clarify this controversy, and associate blood pressures below the formal cut-off for classification of ‘hypertension’ with vascular death, data from a very large population of individuals must be analyzed. The purpose of this study is to use the Clinical Practice Research Datalink (CPRD) to reliably assess the association between systolic and diastolic blood pressure at different thresholds and by different types of patient population.

*Background and rationale*

High blood pressure remains the leading risk factor for mortality and disease worldwide.[1] The global burden of hypertension is expected to increase by 60% from 2000 to 2025, with the largest increase in low- and middle-income countries.[4] Evidence from prospective observational studies have suggested a log-linear relationship between blood pressure and cardiovascular risk, with no apparent threshold below which a reduced blood pressure is not associated with a reduced risk of cardiovascular events.[2] A collaboration of prospective cohort studies based on 900,000 individuals showed that at ages 40–69 years, each difference of 20 mm Hg usual systolic blood pressure (or, approximately 10 mm Hg usual diastolic blood pressure) is associated with more than a twofold difference in the stroke death rate, and with twofold differences in the death rates from ischaemic heart disease (IHD), down to a threshold of 115/75 mm Hg.[2] Similarly, a meta-analysis of blood pressure lowering trials suggests a 22% reduction in IHD events for each 10 mm Hg reduction in systolic BP, with no evidence of any change in risk with different baseline blood pressures.[5]

In light of this evidence, it is clear that hypertension should be tightly controlled in the general population. However, worldwide only half of individuals with a blood pressure elevated above 140/90 mm Hg are aware that they suffer from hypertension, and of those receiving pharmacological treatment for hypertension, only a third have controlled their blood pressure to below 140/90 mm Hg.[6] This poor control of high blood pressure in the public may be due, in part, to residual uncertainties of the effect of blood pressure lowering in different patient subgroups.

Whilst a physiological threshold must exist below which blood pressure levels are associated with increased cardiovascular mortality, current evidence has not established this natural threshold reliably.[3] Reflecting this uncertainty, recent guidelines actually raised the target for blood pressure control in the elderly (age >60 years) from 140 mm Hg to 150 mm Hg.[7] This study aims to clarify these residual uncertainties and determine the threshold for which a lower blood pressure is associated with an increased risk of cardiovascular mortality.

The CPRD stores routinely collected information from UK General Practitioners on blood pressure, cholesterol levels and cardiovascular events. The unique size of this longitudinal database provides a good opportunity to determine the nadir of blood pressure, below which the risk of cardiovascular events is increased. Additionally, analysis of CPRD would allow the association of blood pressure with cardiovascular events to be clarified in sub-groups of individuals, by systolic or diastolic blood pressures, and by different types of cardiovascular outcomes.

*Aims and Objectives*

The aim of this study is to determine the age-specific association between usual blood pressure and cardiovascular mortality in a large sample of individuals with different clinical characteristics (usual blood pressure refers to measured blood pressure corrected for measurement error and time-dependent regression to the mean.). Cardiovascular deaths will be stratified into stroke outcomes, renal outcomes, ischaemic heart disease outcomes and heart failure outcomes, and examined separately.

The key objectives are to:

- Define a baseline systolic and diastolic blood pressure for each individual patient.
- Determine, for each individual patient, whether a cardiovascular event (defined below) occurred, and to measure the at-risk time interval.
- Define a cohort of patients based on baseline systolic blood pressures (SBP), from 85 mm Hg to 205 mm Hg, with 10 mm Hg intervals, and age, from 30 to 80, with 10-year intervals.
- Define a cohort of patients based on baseline diastolic blood pressures (DBP) from 55 mm Hg to 135 mm Hg, with 10 mm Hg intervals, and age, from 30 to 80, with 10-year intervals.
- Determine the number of cardiovascular deaths in each group, stratified into death due to stroke, ischemic heart disease, renal disease, and heart failure.
- Determine the age-specific hazard ratio for each patient subgroup, taking into account regression dilution.

To locate the nadir, the key objectives are to:

- Fit non-parametric curves to the data to continuously predict the hazard ratio for outcomes from SBP and DBP.[8]
- Fit semi-parametric Cox models to estimate the nadir for each outcome.

**Methods**

*Study type*

This is an observational study (both descriptive and hypothesis generating) that utilizes the CPRD, a database generated from clinical encounters and medical records kept with UK general practitioners. The database currently covers approximately 15 million UK patients and is growing. It contains information on medical history, medications and demographics. CPRD data are provided linked to mortality data from the Office of National Statistics.

*Study period*

To maximize the power of the study (see below), the study period will be the longest possible period available. With the requirement of at least one year follow up for individuals included in the analysis, the actual case selection period will start in 01/01/1990 and finish in 01/01/2013 (with follow-up to continue to the last time interval possible).

*Study Population*

- Adults aged 30 or more
- At least one blood pressure measurement (Readcode = 246) performed and recorded in CPRD
- Blood pressure measurement performed between 01/01/1990 and 01/01/2013
- Age recorded
- Registered at the GP for > 1 year period
- At least 1 year of follow up after an initial blood pressure measurement

*Sample size*

We wish to guarantee that we can associate blood pressure with the risk of vascular mortality in the category of 94-104 mm Hg, age 40-49. While we will attempt to associate even lower blood pressures with risk of vascular mortality, this subgroup will be 20 mm Hg lower than the Prospective Studies Collaboration [2] was able to analyze and would represent a significant extension of our current knowledge of the association of blood pressure levels with risk of vascular mortality. Therefore, in order to determine the sample size necessary to allow for accurate association of this subgroup with risk of vascular mortality, the minimum number of events in this subgroup must be estimated. A 2010 analysis of IHD mortality within the CPRD found an overall rate of 110 fatal myocardial infarctions per 100 000 patient years.[9] Therefore, the number of patient-years necessary in this subgroup to obtain a minimum number of events will be calculated using this incidence rate as a baseline (described in further detail below).

The Prospective Studies Collaboration (PSC) analyzed twelve million patient-years. They had a total of 34283 IHD mortality events, an event rate of 280 IHD events per 100 000 patient-years. For the youngest age group with the lowest blood pressure (age 40-49, BP 115 - 124 mm Hg) PSC had a total of approximately 70 IHD events and was able to calculate a hazard ratio with a margin of error of 10%. Therefore, for the lowest category in our study, we should aim to have a total number of 70 IHD events to allow for accurate association of BP with IHD mortality.

Assuming that the lowest systolic BP category (95-104 mm Hg) had 22 IHD mortality events per 100 000 patient years (20% of the population rate – likely a significant underestimate of the true event rate), it would require approximately 300 000 patient-years within this category to obtain 70 IHD events. Approximately, 6% of the UK adult population has a BP within 95-104 mm Hg.[10] Assuming a mean follow-up of seven years, the sample size of our population would have to be 760 000. As CPRD in its current form has 15 million patients, and we have been advised by the Oxford Primary Care site to expect 6 to 8 million patients suitable for analysis, the database will provide us with excellent power to examine the hazard ratio for 95 -104 mm Hg, and will likely allow us to examine blood pressure subgroups that are even lower (85-94 mm Hg and 75-84 mm Hg)

Additionally, a sample size of 6 to 8 million would allow for secondary analyses examining the association of BP with mortality among patient subgroups. Patients with a history of vascular disease (ischemic heart disease, stroke, heart failure and diabetes) will be excluded in the main analysis but evaluated in secondary analyses. The prevalence of CHD, stroke, heart failure and diabetes in the UK is 3.5%, 1.8%, 0.8% and 4.6% respectively. [11,12] A sample size of 8 million would result in a cohort of 64 000 patients with heart failure and a cohort of 368 000 for diabetes, both of which would allow for accurate association of blood pressure with mortality within a range of 125-195 mm Hg SBP.

*Patients’ entry/exit dates from study*

- Entry date: Measurement of blood pressure at GP (see Appendix for read code)
- Study exit date - latest of: date of death, date of leaving practice, end of study period

*Outcome*

*Primary Outcomes*

Mortality (available from linked ONS data)

- Time to fatal CHD event from initial blood pressure measurement (identified through both HES/CPRD data and ONS linked mortality - see Appendix for read codes, ICD-10 codes I20-I25)
- Time to fatal stroke event from initial blood pressure measurement (identified through both HES/CPRD data and ONS linked mortality - see Appendix for read codes, ICD-10 codes I60-I66)
- Time to non-fatal myocardial infarction event (see Appendix for read codes) from initial BP measurement
- Time to non-fatal stroke event (Appendix) from initial BP measurement
- Time to all-cause death

*Secondary Outcomes*

Mortality (available from linked ONS data)

- Time to heart failure death (ICD-10 I50 code listed as any cause of death, not just underlying cause) from initial BP measurement
- Time to renal disease death (ICD-10 code of N17, N18 or N19 listed as any cause of death) from initial BP measurement
- Time to death from aortic aneurysm (ICD-10 I71 code listed as any cause of death)
- Time to death from hypertensive heart disease (ICD-10 I71 code listed as any cause of death)
- Time to death from rheumatic heart disease (ICD-10 I05-I09 listed as any cause of death)
- Time to death from pulmonary embolism (ICD-10 I26 code listed as any cause of death)
- Time to death from cancer (ICD-10 C00-D48 listed as any cause of death)

Non-fatal events (available from linked HES data and MINAP)

- Time to fall (identified through CPRD and HES, see Appendix for read codes)
- Time to fracture (identified through CPRD and HES, see Appendix for read codes)

*Data analysis and statistical plan*

Eligible patients will be defined as any individual included in CPRD who has had a single blood pressure reading (Readcode 246, Appendix) taken. To deal with reverse causality, restrictions will be placed on the individuals allowed to enter into the study. Individuals with a prior history of vascular disease, namely IHD (Read code G3) acute myocardial infarction (Readcode G30), stroke (G66), heart failure (G58) and diabetes (C1) will be excluded from the main analysis. For secondary analyses, individuals with a prior history of vascular disease will be considered as subgroups and examined for heterogeneity relative to those without vascular disease. Publicly available algorithms, previously used to analyze CPRD and described below, will be used to screen for individuals who suffer from vascular disease but have been misrecorded in the CPRD database. [13-15] This will require that medication data be provided and linked to CPRD patients.

Once an eligible population of individuals is defined, subgroups of individuals within specific blood pressure-age ranges will be specified. Individuals will be stratified into 12 subgroups based on their initial blood pressure reading (85-94 mm Hg, 95-104, 105-114, 115-124, 125-134, 135-144, 145-154, 155-164, 165-174, 175-184 and185-194 mm Hg). Their age at death with be stratified into six subgroups (30-39, 40-49, 50-59, 60-69, 70-79 and 80-89). As performed in the Prospective Studies Collaboration, the risk of death for each age subgroup will be analyzed relative to the age at the start of each decade. [2] For example, the risk of death during the age of 50-59 will be related to the estimated usual blood pressure measurement at the age of 50 (not necessarily the age of the initial baseline blood pressure measurement). Patients will thus be able to contribute to multiple subgroups (as a patient who entered the study at 40 and died at 58 would contribute patient-years to both the subgroups of 40-49 and 50-59).

Central mortality data, containing both the date of death and the cause of death, will be linked to CPRD patients’ medical records. For patients who have died, the time to death in years from the date of entry into the study and death will be assigned. For patients who are alive, the time under observation will instead be included. This will produce a flat file of a baseline BP measurement and age for each patient, with any additional BP measurements, a time to death measurement (or no death recorded) and a coded cause of death.

In order to estimate the relationship between usual blood pressure and mortality, rather than measured blood pressure, time-dependent regression dilution must be taken into account. A simple linear regression model will be fit to repeat measurements, with baseline blood pressure as the x-variable and repeat measured blood pressure as the y-variable. The attenuation coefficient is the reciprocal of the slope. As a sensitivity analysis, the nonparametric MacMahon-Peto method will also be used to estimate the attenuation coefficient for each blood pressure-age subgroup. [16,17] The cohort of patients who have repeated blood pressure measurements (likely to be quite large) will be used to estimate the blood pressure at each decade from the initial baseline BP measurement.

Cox proportional hazard models will be used to estimate the cause-specific mortality hazard ratio for each subgroup of patients relative to a baseline subgroup. For the primary analysis, only the age-specific association between blood pressure and vascular mortality will be calculated. However, for secondary analyses, associations will be adjusted for measured cholesterol (44P), deprivation, smoking, sex, body mass index, practice level cluster and treatment with blood pressure lowering drugs. For the primary analysis, floating absolute risks will be used to describe the hazard ratio for each age and blood pressure subgroup.

In order to estimate the nadir for each vascular outcome, non-parametric curves will be fit to the entire dataset to continuously estimate a hazard ratio from systolic and diastolic blood pressure. [8] Additionally, a quadratic Cox proportional hazards function will be fit to the local region around the predicted nadir, if such exists. The estimated location of the nadir of the two approaches will be compared. As a sensitivity analysis, fractional polynomial curves will be fit to the data, to examine how sensitive the location of the nadir is to the modeling approach used.

In order to deal with missing data points within CPRD, non-parametric multiple imputation will be performed. The algorithm to be used is described in greater detail below.

*Sensitivity analyses*

A sensitivity analysis, excluding patients taking blood pressure lowering drugs (defined in the Appendix with read codes), will be performed. Although this may reduce the sample size for patients with BP >150 mm Hg significantly, it will likely have little effect on the cohort of patients < 130 mm Hg.

*Pilot analysis*

A pilot analysis using the methodology described above will be performed on a sample of three hundred thousand patients, selected as a random sample of patients eligible in the above criteria.

*Confounding*

The primary issue related to confounding is reverse causality, that is, that individuals with vascular disease at baseline will have low blood pressure as a result of prior cardiovascular disease and not the other way around. For this reason, a sensitive approached is being proposed to exclude all individuals with possible prior vascular disease in the primary analysis, using both diagnoses recorded in CPRD and medications suggestive of possible vascular disease (discussed in greater detail below).

A separate issue related to confounding is that individuals with high blood pressure will be treated with antihypertensive medication (defined with corresponding read codes in the Appendix). This will reduce the apparent correlation between measured blood pressure and risk of cardiovascular events, as individuals with a high measured blood pressure at baseline have their BP reduced by treatment, reducing their cardiovascular risk. However, this problem is also faced in traditional epidemiological studies, which have successfully controlled for this effect by controlling for time-dependent regression dilution. [2] As CPRD will contain multiple individuals who have had repeated blood pressure measurements, regression dilution in individuals on antihypertensive treatment can be controlled for, by controlling for regression dilution within aggregate age-blood pressure categories. [2]

Finally, confounding by treatment with antihypertensive medication for non-hypertension related diseases must be considered. For example, ACE inhibitors are used in the treatment of heart failure while beta-blockers are used in the treatment of cardiac arrhythmias. Inclusion of these patients may increase the risk of reverse causality, particularly at low blood pressures. Consequently, a sensitivity analysis will be performed with the exclusion of patients on antihypertensives at baseline, to demonstrate that results (particularly in the blood pressure range < 130 mm Hg) are not caused by the inclusion of patients with unobserved vascular disease.

*Limitations in the design*

1. Accurate definition of subgroups of stroke mortality.

As it is difficult to distinguish between hemorrhagic and ischemic stroke, subgroups of stroke mortality may be misclassified by OHS. Consequently, our main analysis will not distinguish between hemorrhagic and ischemic stroke. However, in a secondary analysis, we will use a publicly available validated algorithm to distinguish between hemorrhagic and ischemic stroke based on prescription patterns following stroke events. This algorithm has been previously used in a CPRD analysis associating Alzheimer’s disease with incidence of stroke. [13]

1. Screening of patients with previous history of vascular disease.

If patients with a prior history of vascular disease enter into the assembled cohort, association will be spurious. Clinical diagnoses may not be recorded in CPRD. Consequently, publicly available algorithms, previously used to identify patients with myocardial infarction, stroke and heart failure in CPRD, will be used to screen out ineligible patients. [13-15] For myocardial infarction, this will require linkage to MINAP, to more accurately determine mortality after myocardial infarction.[15] Additionally, if available, open source algorithms from the Farr Institute will be used to screen patients for a history of vascular disease using combinations of medications recorded in CPRD.

1. Missing data within CPRD different from missing data on clinical history described above.

A sensitivity analysis will be performed using multiple imputation to deal with missing data points within CPRD. Traditional multiple imputation has been shown to lead to biased results when dealing with epidemiological data that contains non-linear relationships, such as interaction effects. Consequently, random forest-based multiple imputation, a machine learning technique, will be used to impute missing data points using the MICE algorithm.[18]

*Patient and User Group Involvement*

We intend to discuss our research plans with policy advisers at the British Heart Foundation (BHF). The BHF have previously expressed interest in our work and have been willing to support the dissemination of our research and findings at strategic intervals. The George Institute is committed to service-user involvement and has patient and lay members as part of the institute. Additionally, the George Institute is in the midst of developing a patient advisory group for all research projects occurring at the institute.

*Plans for dissemination of results*

The results of this study will be disseminated through peer-reviewed literature and presented if appropriate at peer reviewed international medical conferences.

References

1 Lim SS, Vos T, Flaxman AD, *et al.* A comparative risk assessment of burden of disease and injury attributable to 67 risk factors and risk factor clusters in 21 regions, 1990-2010: a systematic analysis for the Global Burden of Disease Study 2010. *Lancet* 2012;**380**:2224–60.

2 Lewington S, Clarke R, Qizilbash N, *et al.* Age-specific relevance of usual blood pressure to vascular mortality: a meta-analysis of individual data for one million adults in 61 prospective studies. *Lancet* 2002;**360**:1903–13.

3 Messerli FH, Panjrath GS. The J-curve between blood pressure and coronary artery disease or essential hypertension: exactly how essential? *J Am Coll Cardiol* 2009;**54**:1827–34.

4 Kearney PM, Whelton M, Reynolds K, *et al.* Global burden of hypertension: analysis of worldwide data. *Lancet* 2005;**365**:217–23.

5 Law MR, Morris JK, Wald NJ. Use of blood pressure lowering drugs in the prevention of cardiovascular disease: meta-analysis of 147 randomised trials in the context of expectations from prospective epidemiological studies. *BMJ* 2009;**338**:b1665.

6 Rahimi K, MacMahon S. Blood pressure management in the 21st century: maximizing gains and minimizing waste. *Circulation* 2013;**128**:2283–5.

7 James PA, Oparil S, Carter BL, *et al.* 2014 evidence-based guideline for the management of high blood pressure in adults: report from the panel members appointed to the Eighth Joint National Committee (JNC 8). *JAMA : the journal of the American Medical Association* 2014;**311**:507–20.

8 Ning Y, Woodward M. Relative risk regression models with inverse polynomials. *Statistics in medicine* 2013;**32**:3235–46.

9 Smolina K, Wright FL, Rayner M, *et al.* Determinants of the decline in mortality from acute myocardial infarction in England between 2002 and 2010: linked national database study. *BMJ* 2012;**344**:d8059.

10 Port S, Demer L, Jennrich R, *et al.* Systolic blood pressure and mortality. *Lancet* 2000;**355**:175–80.

11 Townsend N, Wickramasinghe K, Bhatnagar P, *et al.* Coronary Heart Disease Statistics, 2012 edition. *British Heart Foundation* 2012.

12 UK Diabetes. Diabetes in the UK 2012. *London: Diabetes UK* 2012.

13 Imfeld P, Bodmer M, Schuerch M, *et al.* Risk of incident stroke in patients with Alzheimer disease or vascular dementia. *Neurology* 2013;**81**:910–9.

14 Hsieh PH, Hsiao FY. Risk of heart failure associated with dopamine agonists: a nested case-control study. *Drugs Aging* 2013;**30**:739–45.

15 Herrett E, Shah AD, Boggon R, *et al.* Completeness and diagnostic validity of recording acute myocardial infarction events in primary care, hospital care, disease registry, and national mortality records: cohort study. *BMJ* 2013;**346**:f2350.

16 MacMahon S, Peto R, Cutler J, *et al.* Blood pressure, stroke, and coronary heart disease. Part 1, Prolonged differences in blood pressure: prospective observational studies corrected for the regression dilution bias. *Lancet* 1990;**335**:765–74.

17 Clarke R, Shipley M, Lewington S, *et al.* Underestimation of risk associations due to regression dilution in long-term follow-up of prospective studies. *American journal of epidemiology* 1999;**150**:341–53.

18 Shah AD, Bartlett JW, Carpenter J. Comparison of Random Forest and Parametric Imputation Models for Imputing Missing Data Using MICE: A CALIBER Study. *American journal of epidemiology* 2014.

19 Lee S, Shafe ACE, Cowie MR. UK stroke incidence, mortality and cardiovascular risk management 1999-2008: time-trend analysis from the General Practice Research Database. *BMJ Open* 2011;**1**:e000269.

20 Kontopantelis E, Springate D, Reeves D, *et al.* Withdrawing performance indicators: retrospective analysis of general practice performance under UK Quality and Outcomes Framework. *BMJ* 2014;**348**:g330.

**Protocol Amendment**

We wish to propose three amendments to the protocol as currently defined:

1. Addition of new onset diabetes as a secondary outcome and exclusion of individuals in a secondary analysis. This includes time to new onset type 2 diabetes (identified through CPRD and HES, see Appendix for read codes) and time to death from type 2 diabetes (ICD-10 E11 listed as any cause of death).
2. Time to onset of atrial fibrillation as a secondary outcome, both non-fatal (identified through CPRD and HES, see Appendix for read codes) and fatal Time to death from atrial fibrillation (ICD-10 I48 listed as any cause of death)
3. Time to onset of vascular dementia or dementia as a secondary outcome, both non-fatal (identified through CPRD and HES, see Appendix for read codes) and fatal (ICD-10 F01 and F03 listed as any cause of death).

**Protocol Amendment #2**

**The association between blood pressure and cardiovascular events: an analysis of CPRD.**

**ISAC Protocol 14_075**

We wish to propose three amendments to the protocol as currently defined

1. Time to deep vein thrombosis as an outcome, both non-fatal (identified through CPRD and HES, see Appendix for read codes) and fatal (ICD-10 I80-I82 listed as any cause of death)
2. Time to valvular heart disease as an outcome, both non-fatal (identified through CPRD and HES, see Appendix for read codes) and fatal (ICD-10 I34-I37, I05-I08, Q22-Q23 listed as any cause of death).
3. Time to peripheral arterial disease as an outcome, both non-fatal (identified through CPRD and HES, see Appendix for read codes) and fatal (ICD-10 I73.1, I73.8, I73.9, I74.3, I74.4, I74.5 listed as any cause of death)

Appendix: Read Codes

*Blood pressure*

Extraction based on all measurements of Entity Type 1, as is performed in the CALIBER dataset.

*Myocardial infarction (Read codes validated for identification of myocardial infarction*[15]

323..00 ECG: myocardial infarction

3233.00 ECG: antero-septal infarct.

3234.00 ECG:posterior/inferior infarct

3235.00 ECG: subendocardial infarct

3236.00 ECG: lateral infarction

323Z.00 ECG: myocardial infarct NOS

889A.00 Diab mellit insulin-glucose infus acute myocardial infarct

G30..00 Acute myocardial infarction

G30..11 Attack - heart

G30..12 Coronary thrombosis

G30..13 Cardiac rupture following myocardial infarction (MI)

G30..14 Heart attack

G30..15 MI - acute myocardial infarction

G30..16 Thrombosis - coronary

G30..17 Silent myocardial infarction

G300.00 Acute anterolateral infarction

G301.00 Other specified anterior myocardial infarction

G301000 Acute anteroapical infarction

G301100 Acute anteroseptal infarction

G301z00 Anterior myocardial infarction NOS

G302.00 Acute inferolateral infarction

G303.00 Acute inferoposterior infarction

G304.00 Posterior myocardial infarction NOS

G305.00 Lateral myocardial infarction NOS

G306.00 True posterior myocardial infarction

G307.00 Acute subendocardial infarction

G307000 Acute non-Q wave infarction

G307100 Acute non-ST segment elevation myocardial infarction

G308.00 Inferior myocardial infarction NOS

G309.00 Acute Q-wave infarct

G30A.00 Mural thrombosis

G30B.00 Acute posterolateral myocardial infarction

G30X.00 Acute transmural myocardial infarction of unspecif site

G30X000 Acute ST segment elevation myocardial infarction

G30y.00 Other acute myocardial infarction

G30y000 Acute atrial infarction

G30y100 Acute papillary muscle infarction

G30y200 Acute septal infarction

G30yz00 Other acute myocardial infarction NOS

G30z.00 Acute myocardial infarction NOS

G310.11 Dressler's syndrome

G31y100 Microinfarction of heart

G35..00 Subsequent myocardial infarction

G350.00 Subsequent myocardial infarction of anterior wall

G351.00 Subsequent myocardial infarction of inferior wall

G353.00 Subsequent myocardial infarction of other sites

G35X.00 Subsequent myocardial infarction of unspecified site

G36..00 Certain current complication follow acute myocardial infarction

G360.00 Haemopericardium/ complication following acute myocardial infarction

G361.00 Atrial septal defect/ complication following acute myocardial infarction

G362.00 Ventricular septal defect complication following acute myocardial infarction

G363.00 Ruptured cardiac wall w'out haemopericard/ complication following acute myocardial infarction

G364.00 Ruptured chordae tendinae/ complication following acute myocardial infarction

G365.00 Ruptured papillary muscle/ complication following acute myocardial infarction

G366.00 Thrombosis atrium,auric append&vent/curr comp foll acute MI

G38..00 Postoperative myocardial infarction

G380.00 Postoperative transmural myocardial infarction anterior wall

G381.00 Postoperative transmural myocardial infarction inferior wall

G384.00 Postoperative subendocardial myocardial infarction

G38z.00 Postoperative myocardial infarction, unspecified

G501.00 Post infarction pericarditis

Gyu3400 [X]Acute transmural myocardial infarction of unspecif site

MINAP

*Stroke (previously used identify stroke in CPRD* [19]*)*

G66..00 Stroke and cerebrovascular accident unspecified

G66..11 CVA unspecified

G64z.00 Cerebral infarction NOS

G64..11 CVA - cerebral artery occlusion

G66..13 CVA - Cerebrovascular accident unspecified

G61..11 CVA - cerebrovascular accid due to intracerebral haemorrhage

G64..12 Infarction - cerebral

G667.00 Left sided CVA

G668.00 Right sided CVA

G64..13 Stroke due to cerebral arterial occlusion

G66..12 Stroke unspecified

G61..12 Stroke due to intracerebral haemorrhage

G664.00 Cerebellar stroke syndrome

G663.00 Brain stem stroke syndrome

G64z200 Left sided cerebral infarction

G64z300 Right sided cerebral infarction

G640000 Cerebral infarction due to thrombosis of cerebral arteries

G63y000 Cerebral infarct due to thrombosis of precerebral arteries

G68X.00 Sequelae of stroke,not specfd as h'morrhage or infarction

G63y100 Cerebral infarction due to embolism of precerebral arteries

G676000 Cereb infarct due cerebral venous thrombosis, nonpyogenic

G641000 Cerebral infarction due to embolism of cerebral arteries

Gyu6400 [X]Other cerebral infarction

G683.00 Sequelae of cerebral infarction

L440.11 CVA - cerebrovascular accident in the puerperium

G677200 Occlusion and stenosis of posterior cerebral artery

G677000 Occlusion and stenosis of middle cerebral artery

G677100 Occlusion and stenosis of anterior cerebral artery

*Cholesterol measurement*

Cholesterol levels will be extracted as in CALIBER using the intersection of any measurement of entity type 163 (Serum Cholesterol), Ent type 177 (Low density lipoprotein) or entity type 288 (Other laboratory test) with any measurement of read code:

| 44P..00 | Serum cholesterol |
| --- | --- |
| 44P5.00 | Serum HDL cholesterol level |
| 44P6.00 | Serum LDL cholesterol level |
| 44P1.00 | Serum cholesterol normal |
| 9N0J.00 | Seen in cholesterol clinic |
| 44P3.00 | Serum cholesterol raised |
| 1262.11 | FH: Cholesterol high |
| C320z00 | Pure hypercholesterolaemia NOS |
| 13B3.00 | Low cholesterol diet |
| 8BAG.00 | Cholesterol reduction programme |
| 8BAG200 | Cholesterol reduction program - declined |
| 6879.11 | Cholesterol screen |
| ZV65317 | [V]Dietary surveillance in hypercholesterolaemia |
| 44PK.00 | Serum fasting total cholesterol |
| 44PJ.00 | Serum total cholesterol level |
| 44PB.00 | Serum fasting HDL cholesterol level |
| 44PC.00 | Serum random HDL cholesterol level |
| 44d3.00 | Plasma fasting HDL cholesterol level |
| 44PD.00 | Serum fasting LDL cholesterol level |
| 44PI.00 | Calculated LDL cholesterol level |
| 44P7.00 | Serum VLDL cholesterol level |
| 44l2.00 | Cholesterol/HDL ratio |
| 44PG.00 | HDL : total cholesterol ratio |
| 4I3O.00 | Fluid sample cholesterol level |
| 44P8.00 | Serum HDL:non-HDL cholesterol ratio |
| 44lF.00 | Serum cholesterol/HDL ratio |
| 44PF.00 | Total cholesterol:HDL ratio |
| 44OE.00 | Plasma total cholesterol level |
| 44PH.00 | Total cholesterol measurement |
| 662a.00 | Pre-treatment serum cholesterol level |
| C32..11 | Disorder of cholesterol metabolism |
| 44dB.00 | Plasma LDL cholesterol level |
| J656.00 | Cholesterolosis of gallbladder |
| 662X.00 | Target cholesterol level |
| 44PZ.00 | Serum cholesterol NOS |
| 44d2.00 | Plasma random HDL cholesterol level |
| 44P2.00 | Serum cholesterol borderline |
| 44d5.00 | Plasma fasting LDL cholesterol level |
| 9N4K.00 | DNA - Did not attend cholesterol clinic |
| 8I3w.00 | Cholesterol test declined |
| 44d4.00 | Plasma random LDL cholesterol level |
| 44dA.00 | Plasma HDL cholesterol level |
| 44lH.00 | Serum cholesterol/LDL ratio |
| 44P4.00 | Serum cholesterol very high |
| 44P9.00 | Serum cholesterol studies |
| F55y000 | Cholesterol granuloma |
| 8BAG000 | Cholesterol reduction programme - invited |
| 44lG.00 | Plasma cholesterol/HDL ratio |
| 44PE.00 | Serum random LDL cholesterol level |
| 44lI.00 | Plasma cholesterol/LDL ratio |
| 8BAG100 | Cholesterol reduction program - attended |
| 4G21.00 | O/E: cholesterol gall stone |
| 44lJ.00 | Serum cholesterol/VLDL ratio |
| 44PL.00 | Non HDL cholesterol level |
| 4GA6.00 | Calculus = cholesterol |

*Diabetes*

Read codes (previous used in CPRD analysis[20])

| 66A3.00 | Diabetic on diet only |
| --- | --- |
| 66A4.00 | Diabetic on oral treatment |
| 66A5.00 | Diabetic on insulin |
| 66AI.00 | Diabetic - good control |
| 66AJ.00 | Diabetic - poor control |
| 66AJ100 | Brittle diabetes |
| 66AJ.11 | Unstable diabetes |
| 66AJz00 | Diabetic - poor control NOS |
| 66AK.00 | Diabetic - cooperative patient |
| 66AL.00 | Diabetic-uncooperative patient |
| 66AV.00 | Diabetic on insulin and oral treatment |
| C10..00 | Diabetes mellitus |
| C100.00 | Diabetes mellitus with no mention of complication |
| C100000 | Diabetes mellitus; juvenile type; no mention of complication |
| C100011 | Insulin dependent diabetes mellitus |
| C100100 | Diabetes mellitus; adult onset; no mention of complication |
| C100111 | Maturity onset diabetes |
| C100112 | Non-insulin dependent diabetes mellitus |
| C100z00 | Diabetes mellitus NOS with no mention of complication |
| C101.00 | Diabetes mellitus with ketoacidosis |
| C101000 | Diabetes mellitus; juvenile type; with ketoacidosis |
| C101100 | Diabetes mellitus; adult onset; with ketoacidosis |
| C101y00 | Other specified diabetes mellitus with ketoacidosis |
| C101z00 | Diabetes mellitus NOS with ketoacidosis |
| C102.00 | Diabetes mellitus with hyperosmolar coma |
| C102000 | Diabetes mellitus; juvenile type; with hyperosmolar coma |
| C102100 | Diabetes mellitus; adult onset; with hyperosmolar coma |
| C102z00 | Diabetes mellitus NOS with hyperosmolar coma |
| C103.00 | Diabetes mellitus with ketoacidotic coma |
| C103000 | Diabetes mellitus; juvenile type; with ketoacidotic coma |
| C103100 | Diabetes mellitus; adult onset; with ketoacidotic coma |
| C103y00 | Other specified diabetes mellitus with coma |
| C103z00 | Diabetes mellitus NOS with ketoacidotic coma |
| C104.00 | Diabetes mellitus with renal manifestation |
| C104000 | Diabetes mellitus; juvenile type; with renal manifestation |
| C104100 | Diabetes mellitus; adult onset; with renal manifestation |
| C104y00 | Other specified diabetes mellitus with renal complications |
| C104z00 | Diabetes mellitis with nephropathy NOS |
| C105.00 | Diabetes mellitus with ophthalmic manifestation |
| C105000 | Diabetes mellitus; juvenile type; + ophthalmic manifestation |
| C105100 | Diabetes mellitus; adult onset; + ophthalmic manifestation |
| C105y00 | Other specified diabetes mellitus with ophthalmic complicatn |
| C105z00 | Diabetes mellitus NOS with ophthalmic manifestation |
| C106.00 | Diabetes mellitus with neurological manifestation |
| C106000 | Diabetes mellitus; juvenile; + neurological manifestation |
| C106100 | Diabetes mellitus; adult onset; + neurological manifestation |
| C106.12 | Diabetes mellitus with neuropathy |
| C106.13 | Diabetes mellitus with polyneuropathy |
| C106y00 | Other specified diabetes mellitus with neurological comps |
| C106z00 | Diabetes mellitus NOS with neurological manifestation |
| C107.00 | Diabetes mellitus with peripheral circulatory disorder |
| C107000 | Diabetes mellitus; juvenile +peripheral circulatory disorder |
| C107100 | Diabetes mellitus; adult; + peripheral circulatory disorder |
| C107.11 | Diabetes mellitus with gangrene |
| C107.12 | Diabetes with gangrene |
| C107200 | Diabetes mellitus; adult with gangrene |
| C107300 | IDDM with peripheral circulatory disorder |
| C107400 | NIDDM with peripheral circulatory disorder |
| C107z00 | Diabetes mellitus NOS with peripheral circulatory disorder |
| C108.00 | Insulin dependent diabetes mellitus |
| C108000 | Insulin-dependent diabetes mellitus with renal complications |
| C108011 | Type I diabetes mellitus with renal complications |
| C108012 | Type 1 diabetes mellitus with renal complications |
| C108100 | Insulin-dependent diabetes mellitus with ophthalmic comps |
| C108.11 | IDDM-Insulin dependent diabetes mellitus |
| C108.12 | Type 1 diabetes mellitus |
| C108.13 | Type I diabetes mellitus |
| C108200 | Insulin-dependent diabetes mellitus with neurological comps |
| C108211 | Type I diabetes mellitus with neurological complications |
| C108212 | Type 1 diabetes mellitus with neurological complications |
| C108300 | Insulin dependent diabetes mellitus with multiple complicatn |
| C108400 | Unstable insulin dependent diabetes mellitus |
| C108411 | Unstable type I diabetes mellitus |
| C108500 | Insulin dependent diabetes mellitus with ulcer |
| C108511 | Type I diabetes mellitus with ulcer |
| C108600 | Insulin dependent diabetes mellitus with gangrene |
| C108700 | Insulin dependent diabetes mellitus with retinopathy |
| C108711 | Type I diabetes mellitus with retinopathy |
| C108712 | Type 1 diabetes mellitus with retinopathy |
| C108800 | Insulin dependent diabetes mellitus - poor control |
| C108811 | Type I diabetes mellitus - poor control |
| C108812 | Type 1 diabetes mellitus - poor control |
| C108900 | Insulin dependent diabetes maturity onset |
| C108911 | Type I diabetes mellitus maturity onset |
| C108A00 | Insulin-dependent diabetes without complication |
| C108B00 | Insulin dependent diabetes mellitus with mononeuropathy |
| C108B11 | Type I diabetes mellitus with mononeuropathy |
| C108C00 | Insulin dependent diabetes mellitus with polyneuropathy |
| C108D00 | Insulin dependent diabetes mellitus with nephropathy |
| C108D11 | Type I diabetes mellitus with nephropathy |
| C108E00 | Insulin dependent diabetes mellitus with hypoglycaemic coma |
| C108E11 | Type I diabetes mellitus with hypoglycaemic coma |
| C108E12 | Type 1 diabetes mellitus with hypoglycaemic coma |
| C108F00 | Insulin dependent diabetes mellitus with diabetic cataract |
| C108F11 | Type I diabetes mellitus with diabetic cataract |
| C108G00 | Insulin dependent diab mell with peripheral angiopathy |
| C108H00 | Insulin dependent diabetes mellitus with arthropathy |
| C108H11 | Type I diabetes mellitus with arthropathy |
| C108J00 | Insulin dependent diab mell with neuropathic arthropathy |
| C108J12 | Type 1 diabetes mellitus with neuropathic arthropathy |
| C108y00 | Other specified diabetes mellitus with multiple comps |
| C108z00 | Unspecified diabetes mellitus with multiple complications |
| C109.00 | Non-insulin-dependent diabetes mellitus |
| C109000 | Non-insulin-dependent diabetes mellitus with renal comps |
| C109011 | Type II diabetes mellitus with renal complications |
| C109012 | Type 2 diabetes mellitus with renal complications |
| C109100 | Non-insulin-dependent diabetes mellitus with ophthalm comps |
| C109.11 | NIDDM - Non-insulin dependent diabetes mellitus |
| C109111 | Type II diabetes mellitus with ophthalmic complications |
| C109112 | Type 2 diabetes mellitus with ophthalmic complications |
| C109.12 | Type 2 diabetes mellitus |
| C109.13 | Type II diabetes mellitus |
| C109200 | Non-insulin-dependent diabetes mellitus with neuro comps |
| C109211 | Type II diabetes mellitus with neurological complications |
| C109212 | Type 2 diabetes mellitus with neurological complications |
| C109300 | Non-insulin-dependent diabetes mellitus with multiple comps |
| C109400 | Non-insulin dependent diabetes mellitus with ulcer |
| C109411 | Type II diabetes mellitus with ulcer |
| C109412 | Type 2 diabetes mellitus with ulcer |
| C109500 | Non-insulin dependent diabetes mellitus with gangrene |
| C109511 | Type II diabetes mellitus with gangrene |
| C109600 | Non-insulin-dependent diabetes mellitus with retinopathy |
| C109611 | Type II diabetes mellitus with retinopathy |
| C109612 | Type 2 diabetes mellitus with retinopathy |
| C109700 | Non-insulin dependant diabetes mellitus - poor control |
| C109711 | Type II diabetes mellitus - poor control |
| C109712 | Type 2 diabetes mellitus - poor control |
| C109900 | Non-insulin-dependent diabetes mellitus without complication |
| C109A00 | Non-insulin dependent diabetes mellitus with mononeuropathy |
| C109A11 | Type II diabetes mellitus with mononeuropathy |
| C109B00 | Non-insulin dependent diabetes mellitus with polyneuropathy |
| C109B11 | Type II diabetes mellitus with polyneuropathy |
| C109C00 | Non-insulin dependent diabetes mellitus with nephropathy |
| C109C11 | Type II diabetes mellitus with nephropathy |
| C109C12 | Type 2 diabetes mellitus with nephropathy |
| C109D00 | Non-insulin dependent diabetes mellitus with hypoglyca coma |
| C109D11 | Type II diabetes mellitus with hypoglycaemic coma |
| C109D12 | Type 2 diabetes mellitus with hypoglycaemic coma |
| C109E00 | Non-insulin depend diabetes mellitus with diabetic cataract |
| C109E11 | Type II diabetes mellitus with diabetic cataract |
| C109E12 | Type 2 diabetes mellitus with diabetic cataract |
| C109F00 | Non-insulin-dependent d m with peripheral angiopath |
| C109F11 | Type II diabetes mellitus with peripheral angiopathy |
| C109F12 | Type 2 diabetes mellitus with peripheral angiopathy |
| C109G00 | Non-insulin dependent diabetes mellitus with arthropathy |
| C109G11 | Type II diabetes mellitus with arthropathy |
| C109G12 | Type 2 diabetes mellitus with arthropathy |
| C109H00 | Non-insulin dependent d m with neuropathic arthropathy |
| C109H11 | Type II diabetes mellitus with neuropathic arthropathy |
| C109H12 | Type 2 diabetes mellitus with neuropathic arthropathy |
| C109J00 | Insulin treated Type 2 diabetes mellitus |
| C109J11 | Insulin treated non-insulin dependent diabetes mellitus |
| C109J12 | Insulin treated Type II diabetes mellitus |
| C109K00 | Hyperosmolar non-ketotic state in type 2 diabetes mellitus |
| C10C.00 | Diabetes mellitus autosomal dominant |
| C10C.11 | Maturity onset diabetes in youth |
| C10D.00 | Diabetes mellitus autosomal dominant type 2 |
| C10D.11 | Maturity onset diabetes in youth type 2 |
| C10E.00 | Type 1 diabetes mellitus |
| C10E000 | Type 1 diabetes mellitus with renal complications |
| C10E100 | Type 1 diabetes mellitus with ophthalmic complications |
| C10E.11 | Type I diabetes mellitus |
| C10E.12 | Insulin dependent diabetes mellitus |
| C10E200 | Type 1 diabetes mellitus with neurological complications |
| C10E300 | Type 1 diabetes mellitus with multiple complications |
| C10E312 | Insulin dependent diabetes mellitus with multiple complicat |
| C10E400 | Unstable type 1 diabetes mellitus |
| C10E411 | Unstable type I diabetes mellitus |
| C10E412 | Unstable insulin dependent diabetes mellitus |
| C10E500 | Type 1 diabetes mellitus with ulcer |
| C10E600 | Type 1 diabetes mellitus with gangrene |
| C10E700 | Type 1 diabetes mellitus with retinopathy |
| C10E800 | Type 1 diabetes mellitus - poor control |
| C10E812 | Insulin dependent diabetes mellitus - poor control |
| C10E900 | Type 1 diabetes mellitus maturity onset |
| C10EA00 | Type 1 diabetes mellitus without complication |
| C10EA11 | Type I diabetes mellitus without complication |
| C10EB00 | Type 1 diabetes mellitus with mononeuropathy |
| C10EC00 | Type 1 diabetes mellitus with polyneuropathy |
| C10ED00 | Type 1 diabetes mellitus with nephropathy |
| C10EE00 | Type 1 diabetes mellitus with hypoglycaemic coma |
| C10EF00 | Type 1 diabetes mellitus with diabetic cataract |
| C10EG00 | Type 1 diabetes mellitus with peripheral angiopathy |
| C10EH00 | Type 1 diabetes mellitus with arthropathy |
| C10EJ00 | Type 1 diabetes mellitus with neuropathic arthropathy |
| C10EK00 | Type 1 diabetes mellitus with persistent proteinuria |
| C10EL00 | Type 1 diabetes mellitus with persistent microalbuminuria |
| C10EM00 | Type 1 diabetes mellitus with ketoacidosis |
| C10EM11 | Type I diabetes mellitus with ketoacidosis |
| C10EN00 | Type 1 diabetes mellitus with ketoacidotic coma |
| C10EN11 | Type I diabetes mellitus with ketoacidotic coma |
| C10EP00 | Type 1 diabetes mellitus with exudative maculopathy |
| C10EQ00 | Type 1 diabetes mellitus with gastroparesis |
| C10F.00 | Type 2 diabetes mellitus |
| C10F000 | Type 2 diabetes mellitus with renal complications |
| C10F011 | Type II diabetes mellitus with renal complications |
| C10F100 | Type 2 diabetes mellitus with ophthalmic complications |
| C10F.11 | Type II diabetes mellitus |
| C10F200 | Type 2 diabetes mellitus with neurological complications |
| C10F300 | Type 2 diabetes mellitus with multiple complications |
| C10F311 | Type II diabetes mellitus with multiple complications |
| C10F400 | Type 2 diabetes mellitus with ulcer |
| C10F500 | Type 2 diabetes mellitus with gangrene |
| C10F511 | Type II diabetes mellitus with gangrene |
| C10F600 | Type 2 diabetes mellitus with retinopathy |
| C10F611 | Type II diabetes mellitus with retinopathy |
| C10F700 | Type 2 diabetes mellitus - poor control |
| C10F711 | Type II diabetes mellitus - poor control |
| C10F900 | Type 2 diabetes mellitus without complication |
| C10F911 | Type II diabetes mellitus without complication |
| C10FA00 | Type 2 diabetes mellitus with mononeuropathy |
| C10FB00 | Type 2 diabetes mellitus with polyneuropathy |
| C10FB11 | Type II diabetes mellitus with polyneuropathy |
| C10FC00 | Type 2 diabetes mellitus with nephropathy |
| C10FC11 | Type II diabetes mellitus with nephropathy |
| C10FD00 | Type 2 diabetes mellitus with hypoglycaemic coma |
| C10FE00 | Type 2 diabetes mellitus with diabetic cataract |
| C10FF00 | Type 2 diabetes mellitus with peripheral angiopathy |
| C10FG00 | Type 2 diabetes mellitus with arthropathy |
| C10FH00 | Type 2 diabetes mellitus with neuropathic arthropathy |
| C10FJ00 | Insulin treated Type 2 diabetes mellitus |
| C10FJ11 | Insulin treated Type II diabetes mellitus |
| C10FK00 | Hyperosmolar non-ketotic state in type 2 diabetes mellitus |
| C10FL00 | Type 2 diabetes mellitus with persistent proteinuria |
| C10FL11 | Type II diabetes mellitus with persistent proteinuria |
| C10FM00 | Type 2 diabetes mellitus with persistent microalbuminuria |
| C10FN00 | Type 2 diabetes mellitus with ketoacidosis |
| C10FP00 | Type 2 diabetes mellitus with ketoacidotic coma |
| C10FQ00 | Type 2 diabetes mellitus with exudative maculopathy |
| C10FR00 | Type 2 diabetes mellitus with gastroparesis |
| C10G.00 | Secondary pancreatic diabetes mellitus |
| C10G000 | Secondary pancreatic diabetes mellitus without complication |
| C10y.00 | Diabetes mellitus with other specified manifestation |
| C10y100 | Diabetes mellitus; adult; + other specified manifestation |
| C10yy00 | Other specified diabetes mellitus with other spec comps |
| C10yz00 | Diabetes mellitus NOS with other specified manifestation |
| C10z.00 | Diabetes mellitus with unspecified complication |
| C10z000 | Diabetes mellitus; juvenile type; + unspecified complication |
| C10z100 | Diabetes mellitus; adult onset; + unspecified complication |
| C10zy00 | Other specified diabetes mellitus with unspecified comps |
| C10zz00 | Diabetes mellitus NOS with unspecified complication |
| Cyu2.00 | [X]Diabetes mellitus |
| Cyu2000 | [X]Other specified diabetes mellitus |
| Cyu2300 | [X]Unspecified diabetes mellitus with renal complications |
| L180500 | Pre-existing diabetes mellitus; insulin-dependent |
| L180600 | Pre-existing diabetes mellitus; non-insulin-dependent |
| L180X00 | Pre-existing diabetes mellitus; unspecified |

*Smoking*

Entity Type 4.

Read codes:

137..00 Tobacco consumption

1371 Never smoked tobacco

137..11 Smoker - amount smoked

1371.11 Non-smoker

1372 Trivial smoker - < 1 cig/day

1372.11 Occasional smoker

1373 Light smoker - 1-9 cigs/day

1374 Moderate smoker - 10-19 cigs/d

1375 Heavy smoker - 20-39 cigs/day

1376 Very heavy smoker - 40+cigs/d

1377 Ex-trivial smoker (<1/day)

1378 Ex-light smoker (1-9/day)

1379 Ex-moderate smoker (10-19/day)

137a.00 Pipe tobacco consumption

137A.00 Ex-heavy smoker (20-39/day)

137b.00 Ready to stop smoking

137B.00 Ex-very heavy smoker (40+/day)

137c.00 Thinking about stopping smoking

137C.00 Keeps trying to stop smoking

137d.00 Not interested in stopping smoking

137D.00 Admitted tobacco cons untrue ?

137e.00 Smoking restarted

137E.00 Tobacco consumption unknown

137f.00 Reason for restarting smoking

137F.00 Ex-smoker - amount unknown

137g.00 Cigarette pack-years

137G.00 Trying to give up smoking

137h.00 Minutes from waking to first tobacco consumption

137H.00 Pipe smoker

137I.00 Passive smoker

137J.00 Cigar smoker

137K.00 Stopped smoking

137L.00 Current non-smoker

137M.00 Rolls own cigarettes

137N.00 Ex pipe smoker

137O.00 Ex cigar smoker

137P.00 Cigarette smoker

137P.11 Smoker

137Q.00 Smoking started

137Q.11 Smoking restarted

137R.00 Current smoker

137S.00 Ex smoker

137T.00 Date ceased smoking

137U.00 Not a passive smoker

137V.00 Smoking reduced

137W.00 Chews tobacco

137X.00 Cigarette consumption

137Y.00 Cigar consumption

137Z.00 Tobacco consumption NOS

13cA.00 Smokes drugs

13p..00 Smoking cessation milestones

13p0.00 Negotiated date for cessation of smoking

13p1.00 Smoking status at 4 weeks

13p2.00 Smoking status between 4 and 52 weeks

13p3.00 Smoking status at 52 weeks

13p4.00 Smoking free weeks

13p5.00 Smoking cessation programme start date

13WF.11Smoker in the family

13WK.00 No smokers in the household

177..00 Smoke inhalation

6791 Health ed. - smoking

67A3.00 Pregnancy smoking advice

67H1.00 Lifestyle advice regarding smoking

745H.00 Smoking cessation therapy

745H000 Nicotine replacement therapy using nicotine patches

745H100 Nicotine replacement therapy using nicotine gum

745H200 Nicotine replacement therapy using nicotine inhalator

745H300 Nicotine replacement therapy using nicotine lozenges

8B2B.00 Nicotine replacement therapy

8B3f.00 Nicotine replacement therapy provided free

8B3Y.00 Over the counter nicotine replacement therapy

8BP3.00 Nicotine replacement therapy provided by community pharmacis

8CAg.00 Smoking cessation advice provided by community pharmacist

8CAL.00 Smoking cessation advice

8H7i.00 Referral to smoking cessation advisor

8HTK.00 Referral to stop-smoking clinic

8I2I.00 Nicotine replacement therapy contraindicated

8I39.00 Nicotine replacement therapy refused

8I6H.00 Smoking review not indicated

9hG..00 Exception reporting: smoking quality indicators

9hG0.00 Excepted from smoking quality indicators: Patient unsuitable

9hG1.00 Excepted from smoking quality indicators: Informed dissent

9N2k.00 Seen by smoking cessation advisor

9N4M.00 DNA - Did not attend smoking cessation clinic

9OO..00 Anti-smoking monitoring admin.

9OO1.00 Attends stop smoking monitor.

9OO..11 Stop smoking clinic admin.

9OO..12 Stop smoking monitoring admin.

9OO2.00 Refuses stop smoking monitor

9OO3.00 Stop smoking monitor default

9OO4.00 Stop smoking monitor 1st lettr

9OO5.00 Stop smoking monitor 2nd lettr

9OO6.00 Stop smoking monitor 3rd lettr

9OO7.00 Stop smoking monitor verb.inv.

9OO8.00 Stop smoking monitor phone inv

9OO9.00 Stop smoking monitoring delete

9OOA.00 Stop smoking monitor.chck done

9OOZ.00 Stop smoking monitor admin.NOS

H310100 Smokers' cough

SM7y200 Smoke inhalation

SM7z.11 Smoke inhalation

ZG23300 Advice on smoking

ZRaM.00 Motives for smoking scale

ZRh4.00 Reasons for smoking scale

ZRh4.11 RFS - Reasons for smoking scale

Atrial fibrillation

| readcode | desc |
| --- | --- |
| 3272 | ECG: atrial fibrillation |
| 3273 | ECG: atrial flutter |
| 3274 | ECG: paroxysmal atrial tachy. |
| 7936900 | Implantation of intravenous atrial overdrive pacemaker |
| 14AN.00 | H/O: atrial fibrillation |
| 14AR.00 | History of atrial flutter |
| 662S.00 | Atrial fibrillation monitoring |
| 6A9..00 | Atrial fibrillation annual review |
| 7936A00 | Implant intravenous pacemaker for atrial fibrillation |
| 793M100 | Perc transluminal ablation of atrial wall for atrial flutter |
| 9hF..00 | Exception reporting: atrial fibrillation quality indicators |
| 9hF1.00 | Excepted from atrial fibrillation qual indic: Inform dissent |
| 9Os..00 | Atrial fibrillation monitoring administration |
| 9Os0.00 | Atrial fibrillation monitoring first letter |
| 9Os1.00 | Atrial fibrillation monitoring second letter |
| 9Os2.00 | Atrial fibrillation monitoring third letter |
| 9Os3.00 | Atrial fibrillation monitoring verbal invite |
| 9Os4.00 | Atrial fibrillation monitoring telephone invite |
| G570000 | Paroxysmal atrial tachycardia |
| G573.00 | Atrial fibrillation and flutter |
| G573000 | Atrial fibrillation |
| G573100 | Atrial flutter |
| G573200 | Paroxysmal atrial fibrillation |
| G573300 | Non-rheumatic atrial fibrillation |
| G573400 | Permanent atrial fibrillation |
| G573500 | Persistent atrial fibrillation |
| G573z00 | Atrial fibrillation and flutter NOS |
| G576300 | Atrial premature depolarization |

Dementia

| readcode | desc |
| --- | --- |
| 1461 | H/O: dementia |
| E00..11 | Senile dementia |
| E00..12 | Senile/presenile dementia |
| E000.00 | Uncomplicated senile dementia |
| E001.00 | Presenile dementia |
| E001000 | Uncomplicated presenile dementia |
| E001100 | Presenile dementia with delirium |
| E001200 | Presenile dementia with paranoia |
| E001300 | Presenile dementia with depression |
| E001z00 | Presenile dementia NOS |
| E002.00 | Senile dementia with depressive or paranoid features |
| E002000 | Senile dementia with paranoia |
| E002100 | Senile dementia with depression |
| E002z00 | Senile dementia with depressive or paranoid features NOS |
| E003.00 | Senile dementia with delirium |
| E004.00 | Arteriosclerotic dementia |
| E004.11 | Multi infarct dementia |
| E004000 | Uncomplicated arteriosclerotic dementia |
| E004100 | Arteriosclerotic dementia with delirium |
| E004200 | Arteriosclerotic dementia with paranoia |
| E004300 | Arteriosclerotic dementia with depression |
| E004z00 | Arteriosclerotic dementia NOS |
| E041.00 | Dementia in conditions EC |
| Eu00.00 | [X]Dementia in Alzheimer's disease |
| Eu00000 | [X]Dementia in Alzheimer's disease with early onset |
| Eu00011 | [X]Presenile dementia,Alzheimer's type |
| Eu00012 | [X]Primary degen dementia, Alzheimer's type, presenile onset |
| Eu00013 | [X]Alzheimer's disease type 2 |
| Eu00100 | [X]Dementia in Alzheimer's disease with late onset |
| Eu00111 | [X]Alzheimer's disease type 1 |
| Eu00112 | [X]Senile dementia,Alzheimer's type |
| Eu00113 | [X]Primary degen dementia of Alzheimer's type, senile onset |
| Eu00200 | [X]Dementia in Alzheimer's dis, atypical or mixed type |
| Eu00z00 | [X]Dementia in Alzheimer's disease, unspecified |
| Eu00z11 | [X]Alzheimer's dementia unspec |
| Eu01.00 | [X]Vascular dementia |
| Eu01.11 | [X]Arteriosclerotic dementia |
| Eu01000 | [X]Vascular dementia of acute onset |
| Eu01100 | [X]Multi-infarct dementia |
| Eu01111 | [X]Predominantly cortical dementia |
| Eu01200 | [X]Subcortical vascular dementia |
| Eu01300 | [X]Mixed cortical and subcortical vascular dementia |
| Eu01y00 | [X]Other vascular dementia |
| Eu01z00 | [X]Vascular dementia, unspecified |
| Eu02z00 | [X] Unspecified dementia |
| Eu02z11 | [X] Presenile dementia NOS |
| Eu02z13 | [X] Primary degenerative dementia NOS |
| Eu02z14 | [X] Senile dementia NOS |
| Eu02z16 | [X] Senile dementia, depressed or paranoid type |
| Eu04100 | [X]Delirium superimposed on dementia |
| F110.00 | Alzheimer's disease |
| F110000 | Alzheimer's disease with early onset |
| F110100 | Alzheimer's disease with late onset |
| Fyu3000 | [X]Other Alzheimer's disease |

Peripheral Arterial Disease

| 14NB.00 | H/O: Peripheral vascular disease procedure |
| --- | --- |
| 662U.00 | Peripheral vascular disease monitoring |
| 7A10100 | Bypass aorta by anastomosis axillary to femoral artery NEC |
| 7A12100 | Bypass bifurc aorta by anastom aorta to femoral artery NEC |
| 7A12300 | Bypass bifurcation aorta by anastom aorta to iliac artery |
| 7A22000 | Percutaneous transluminal angioplasty of carotid artery |
| 7A27C00 | Operation on aneurysm of subclavian artery |
| 7A27D00 | Operation on aneurysm of axillary artery |
| 7A27E00 | Operation on aneurysm of brachial artery |
| 7A28000 | Percutaneous transluminal angioplasty of subclavian artery |
| 7A28100 | Percutaneous transluminal angioplasty of brachial artery |
| 7A28200 | Percutaneous transluminal angioplasty of vertebral artery |
| 7A28C00 | Percutaneous transluminal angioplasty of axillary artery |
| 7A31300 | Operation on aneurysm of renal artery |
| 7A32000 | Percutaneous transluminal angioplasty of renal artery |
| 7A34D00 | Operation on aneurysm of superior mesenteric artery NEC |
| 7A34E00 | Operation on aneurysm of inferior mesenteric artery NEC |
| 7A34F00 | Operation on aneurysm of suprarenal artery NEC |
| 7A35000 | Percutaneous transluminal angioplasty of coeliac artery NEC |
| 7A35300 | Percutaneous transluminal angioplasty suprarenal artery NEC |
| 7A40.00 | Replacement of aneurysmal iliac artery |
| 7A40.11 | Replacement of aneurysmal iliac artery by anastomosis |
| 7A40000 | Emerg replace aneurysm iliac art by iliac/femoral art anast |
| 7A40A00 | Replace aneurysm iliac art by aorta/ext iliac art anast NEC |
| 7A40y00 | Other specified replacement of aneurysmal iliac artery |
| 7A40z00 | Replacement of aneurysmal iliac artery NOS |
| 7A41.00 | Other bypass of iliac artery |
| 7A41.11 | Other bypass of iliac artery by anastomosis |
| 7A41100 | Bypass iliac artery by iliac/femoral artery anastomosis NEC |
| 7A41200 | Emerg bypass iliac artery by femoral/femoral art anast NEC |
| 7A41300 | Bypass iliac artery by femoral/femoral art anastomosis NEC |
| 7A41900 | Bypass common iliac artery by aorta/com iliac art anast NEC |
| 7A41D00 | Bypass iliac artery by iliac/iliac artery anastomosis NEC |
| 7A41y00 | Other specified other bypass of iliac artery |
| 7A41z00 | Other bypass of iliac artery NOS |
| 7A43200 | Operation on aneurysm of iliac artery NEC |
| 7A44000 | Percutaneous transluminal angioplasty of iliac artery |
| 7A45.00 | Emergency replacement of aneurysmal femoral/popliteal artery |
| 7A45.12 | Emergency replacement of aneurysmal common femoral artery |
| 7A45.14 | Emergency replacement of aneurysmal popliteal artery |
| 7A45.15 | Emergency replacement aneurysmal superficial femoral artery |
| 7A45000 | Emerg replace aneurysm fem art by fem/pop art anast c prosth |
| 7A45200 | Emerg replace aneurysm fem art by fem/pop anast c vein graft |
| 7A45700 | Emerg replace aneurysm pop art by pop/tib anast c vein graft |
| 7A45D00 | Emerg replace aneurysm pop artery by pop/fem art anastomosis |
| 7A45y00 | Emergency replacement aneurysmal femoral/popliteal artery OS |
| 7A46.00 | Other replacement of aneurysmal femoral artery |
| 7A46.11 | Other replacement aneurysmal femoral artery by anastomosis |
| 7A46.14 | Other replacement of aneurysmal popliteal artery |
| 7A46.15 | Other replacement of aneurysmal superficial femoral artery |
| 7A46000 | Replace aneurysm fem art by fem/pop art anastom c prosth NEC |
| 7A46100 | Replace aneurysm pop art by pop/pop art anastom c prosth NEC |
| 7A46300 | Replace aneurysm pop art by pop/pop a anast c vein graft NEC |
| 7A46C00 | Replace aneurysm fem artery by fem/fem art anastomosis NEC |
| 7A46D00 | Replace aneurysm popliteal artery by pop/fem anastomosis NEC |
| 7A46y00 | Other replacement of aneurysmal femoral/popliteal artery OS |
| 7A46z00 | Other replacement of aneurysmal femoral/popliteal artery NOS |
| 7A47.00 | Other emergency bypass of femoral artery or popliteal artery |
| 7A47.12 | Other emergency bypass of common femoral artery |
| 7A47.13 | Other emergency bypass of deep femoral artery |
| 7A47.15 | Other emergency bypass of superficial femoral artery |
| 7A47.16 | Other emergency bypass of femoral artery |
| 7A47C00 | Emerg bypass femoral artery by fem/fem art anastomosis NEC |
| 7A48.00 | Other bypass of femoral artery or popliteal artery |
| 7A48.12 | Other bypass of common femoral artery |
| 7A48.14 | Other bypass of femoral artery |
| 7A48.16 | Other bypass of superficial femoral artery |
| 7A48000 | Bypass femoral artery by fem/pop art anast c prosthesis NEC |
| 7A48200 | Bypass femoral artery by fem/pop art anast c vein graft NEC |
| 7A48400 | Bypass femoral artery by fem/tib art anast c prosthesis NEC |
| 7A48600 | Bypass femoral artery by fem/tib art anast c vein graft NEC |
| 7A48800 | Bypass femoral artery by fem/peron a anast c prosthesis NEC |
| 7A48A00 | Bypass femoral artery by fem/peron a anast c vein graft NEC |
| 7A48C00 | Bypass femoral artery by femoral/femoral art anastomosis NEC |
| 7A48y00 | Other bypass of femoral artery or popliteal artery OS |
| 7A48z00 | Other bypass of femoral artery or popliteal artery NOS |
| 7A4A400 | Ligation of aneurysm of popliteal artery |
| 7A4A500 | Operation on aneurysm of femoral artery NEC |
| 7A4B000 | Percutaneous transluminal angioplasty of femoral artery |
| 7A4B100 | Percutaneous transluminal angioplasty of popliteal artery |
| 9N4h.00 | DNA - Did not attend peripheral vascular disease clinic |
| C109F11 | Type II diabetes mellitus with peripheral angiopathy |
| C109F12 | Type 2 diabetes mellitus with peripheral angiopathy |
| C10EG00 | Type 1 diabetes mellitus with peripheral angiopathy |
| C10FF00 | Type 2 diabetes mellitus with peripheral angiopathy |
| C10FF11 | Type II diabetes mellitus with peripheral angiopathy |
| G720.00 | Aneurysm of artery of arm |
| G720000 | Aneurysm of brachial artery |
| G720100 | Aneurysm of radial artery |
| G720200 | Aneurysm of ulnar artery |
| G720z00 | Aneurysm of arm artery NOS |
| G722.00 | Aneurysm of iliac artery |
| G722000 | Aneurysm of common iliac artery |
| G722100 | Aneurysm of external iliac artery |
| G722200 | Aneurysm of internal iliac artery |
| G722z00 | Aneurysm of iliac artery NOS |
| G723.00 | Aneurysm of leg artery |
| G723000 | Aneurysm of femoral artery |
| G723100 | Aneurysm of popliteal artery |
| G723200 | Aneurysm of anterior tibial artery |
| G723300 | Aneurysm of dorsalis pedis artery |
| G723400 | Aneurysm of posterior tibial artery |
| G723500 | Ruptured popliteal artery aneurysm |
| G723600 | Post radiological femoral false aneurysm |
| G723z00 | Aneurysm of leg artery NOS |
| G72y400 | Aneurysm of subclavian artery |
| G72y500 | Aneurysm of splenic artery |
| G72y600 | Aneurysm of axillary artery |
| G72y700 | Aneurysm of coeliac artery |
| G72y800 | Aneurysm of superior mesenteric artery |
| G72y900 | Aneurysm of inferior mesenteric artery |
| G72yA00 | Aneurysm of hepatic artery |
| G73..00 | Other peripheral vascular disease |
| G731.00 | Thromboangiitis obliterans |
| G731000 | Buerger's disease |
| G73y.00 | Other specified peripheral vascular disease |
| G73y000 | Diabetic peripheral angiopathy |
| G73yz00 | Other specified peripheral vascular disease NOS |
| G73z.00 | Peripheral vascular disease NOS |
| G73z000 | Intermittent claudication |
| G73zz00 | Peripheral vascular disease NOS |
| G76z100 | Femoral artery occlusion |
| G76z200 | Popliteal artery occlusion |
| Gyu7400 | [X]Other specified peripheral vascular diseases |

Venous Thromboembolism (both pulmonary embolism and deep vein thrombosis)

| F051.00 | Thrombosis of central nervous system venous sinuses |
| --- | --- |
| F051000 | Thrombosis cavernous sinus |
| F051100 | Thrombosis of superior longitudinal sinus |
| F051200 | Thrombosis lateral sinus |
| F051300 | Thrombosis transverse sinus |
| F051z00 | Thrombosis of central nervous system venous sinus NOS |
| F423811 | Retinal vein thrombosis |
| G401 | Pulmonary embolism |
| G401-1 | Infarction—pulmonary |
| G401-2 | Pulmonary embolus |
| G4010 | Postoperative pulmonary embolus |
| G676 | Non-pyogenic venous sinus thrombosis |
| G801-1 | DVT |
| G801-2 | DVT, leg |
| G801-3 | DVT |
| G801-99 | DVT—leg |
| G801C | DVT of leg related to air travel |
| G801D | Deep vein thrombosis of lower limb |
| G801D-99 | DVT—leg |
| G801E | DVT of leg related to intravenous drug use |
| G801F | DVT of peroneal vein |
| G820 | Budd-Chiari syndrome (hepatic vein thrombosis) |
| G820-1 | Hepatic vein thrombosis |
| G822 | Embolism and thrombosis of the vena cava |
| G823 | Embolism and thrombosis of the renal vein |
| G824.00 | Axillary vein thrombosis |
| G825.00 | Thrombosis of subclavian vein |
| G826.00 | Thrombosis of internal jugular vein |
| G827 | Thrombosis of external jugular vein |
| G82y.00 | Other embolism and thrombosis |
| G82z.00 | Embolism and thrombosis NOS |
| G82z0 | Venous embolism NOS |
| G82z1 | Venous thrombosis NOS |
| G82zz00 | Embolism and thrombosis NOS |
| SP122 | Postoperative deep vein thrombosis |

*Valvular Heart Disease (excludes congenital valve disease)*

939 G54zz00 Endocarditis, valve unspecified, NOS

1267 G11..00 Mitral valve diseases

1294 G540.15 Mitral valve prolapse

2817 G542.00 Tricuspid valve disorders, non-rheumatic

2977 G540.00 Mitral valve incompetence

3169 791z.00 Heart valve and adjacent structures operations NOS

3731 7910300 Replacement of mitral valve NEC

3911 7910.12 Replacement of mitral valve

4900 7918.00 Other open operations on valve of heart

5643 7914300 Replacement of valve of heart NEC

5743 G54z500 Valvular heart disease

5971 7N40000 [SO]Mitral valve

6631 14S4.00 H/O: heart valve recipient

7276 7914200 Prosthetic replacement of valve of heart NEC

7894 7910.00 Plastic repair of mitral valve

8000 7N40.00 [SO]Valve of heart

9396 7918000 Annuloplasty of mitral valve

9450 G540.14 Mitral valve regurgitation

10111 G54z100 Stenosis of unspecified heart valve

11865 7N40300 [SO]Pulmonary valve

12244 7N40200 [SO]Tricuspid valve

12312 G543.00 Pulmonary valve disorders

15910 7914z00 Plastic repair of unspecified valve of heart NOS

16373 G140.00 Tricuspid valve disease NEC

16544 7916z11 Heart valvotomy NEC

16545 G11..11 Rheumatic mitral valve disease

16636 7916.11 Open heart valvotomy

17257 7915000 Revision of plastic repair of mitral valve

17334 14T3.00 H/O: artificial heart valve

17812 7916000 Open mitral valvotomy

18475 G544200 Combined disorders of mitral, aortic and tricuspid valves

18780 ZV42200 [V]Heart valve transplanted

19246 7917000 Closed mitral valvotomy

19390 7910200 Prosthetic replacement of mitral valve

19699 G544100 Disorders of both mitral and tricuspid valves

19957 G543z00 Pulmonary valve disorders NOS

20221 7917z00 Closed incision of valve of heart NOS

22003 G54z013 Regurgitation of unspecified heart valve

22582 7910400 Mitral valvuloplasty NEC

24557 G540z00 Mitral valve disorders NOS

26153 7918100 Annuloplasty of tricuspid valve

26168 7912.00 Plastic repair of tricuspid valve

26626 P6z0.00 Unspecified anomaly of heart valve

28662 G113.00 Nonrheumatic mitral valve stenosis

28850 G54..11 Heart valve disorders - non rheumatic

28871 ZV43300 [V]Has artificial heart valve

29158 G13z.00 Mitral and aortic valve disease NOS

29481 7917.11 Closed heart valvotomy

29732 7916300 Open pulmonary valvotomy

29887 7914.11 Replacement of unspecified valve of heart

30173 791..00 Valves of heart and adjacent structures operations

30443 G11z.00 Mitral valve disease NOS

30567 7910211 Bjork-Shiley prosthetic replacement of mitral valve

30705 7918200 Annuloplasty of valve of heart NEC

31839 G540200 Mitral valve prolapse

31979 G54z.00 Endocarditis, valve unspecified

32930 7912300 Replacement of tricuspid valve NEC

34932 G543400 Pulmonary valve stenosis with insufficiency

36638 7910.11 Mitral valvuloplasty

36734 7914212 Starr prosthetic replacement of valve of heart

36768 G141z00 Rheumatic pulmonary valve disease NOS

36795 7919300 Percutaneous transluminal pulmonary valvotomy

37451 P6yy700 Atresia of heart valve NEC

38607 7917300 Closed pulmonary valvotomy

34958 7911.11 Aortic valvuloplasty

1756 7911.12 Replacement of aortic valve

35812 7911200 Prosthetic replacement of aortic valve

4548 G541.00 Aortic valve disorders

9498 7911.00 Plastic repair of aortic valve

10078 G13..00 Diseases of mitral and aortic valves

10964 G541400 Aortic valve stenosis with insufficiency

10964 G541400 Aortic valve stenosis with insufficiency

15133 7911300 Replacement of aortic valve NEC

17141 7916100 Open aortic valvotomy

18100 G12..00 Rheumatic aortic valve disease

18475 G544200 Combined disorders of mitral, aortic and tricuspid valves

19019 G541z00 Aortic valve disorders NOS

29158 G13z.00 Mitral and aortic valve disease NOS

30610 G541600 Aortic valve sclerosis

34958 7911.11 Aortic valvuloplasty

35812 7911200 Prosthetic replacement of aortic valve

38894 7913.12 Replacement of pulmonary valve

39671 G54z000 Incompetence of unspecified heart valve

39763 7911100 Xenograft replacement of aortic valve

39828 7919100 Percutaneous transluminal aortic valvotomy

39916 G540300 Mitral valve leaf prolapse

39972 7915300 Revision of plastic repair of pulmonary valve

39977 7919.00 Therapeutic transluminal operations on valve of heart

39992 P60..00 Pulmonary valve anomalies

39996 7919.11 Percutaneous transluminal valvotomy

40062 790D.00 Creation of valved cardiac conduit

40086 7911y00 Other specified plastic repair of aortic valve

40239 G544.00 Multiple valve diseases

40264 ZV45H00 [V]Presence of prosthetic heart valve

40582 G544000 Disorders of both aortic and tricuspid valves

40949 G540.12 Mitral valve insufficiency

41168 7910z00 Plastic repair of mitral valve NOS

41169 7915.00 Revision of plastic repair of valve of heart

42748 7917100 Closed aortic valvotomy

43492 7931000 Inspection of valve of heart

43778 7911000 Allograft replacement of aortic valve

43855 G542z00 Tricuspid valve disorders NOS

43923 7913000 Allograft replacement of pulmonary valve

44167 G141.00 Rheumatic pulmonary valve disease

44690 7918y00 Other specified other open operation on valve of heart

44767 P6yy.11 Hypoplastic aortic orifice or valve

45256 7915100 Revision of plastic repair of aortic valve

45283 791A.00 Remove obstruction from structure adjacent to valve of heart

46135 7914211 Edwards prosthetic replacement of valve of heart

46836 7913.00 Plastic repair of pulmonary valve

47014 7912.11 Replacement of tricuspid valve

48333 791y.00 Heart valve or adjacent structures operations OS

49185 G541700 Aortic valve calcification

49272 Gyu5600 [X]Other aortic valve disorders

49338 TB01200 Implant of heart valve prosthesis + complication, no blame

49379 7918z00 Other open operation on valve of heart NOS

49413 7916.00 Open incision of heart valve

49592 7910213 Carpentier prosthetic replacement of mitral valve

50398 7914400 Valvuloplasty of heart NEC

50649 7913400 Pulmonary valvuloplasty NEC

50809 G12z.00 Rheumatic aortic valve disease NOS

51472 G54z300 Endocarditis, valve unspecified, OS

51658 7910100 Xenograft replacement of mitral valve

51940 7917311 Brock pulmonary valvulotomy

52271 G542200 Nonrheumatic tricuspid valve stenosis with insufficiency

53164 7911400 Aortic valvuloplasty NEC

53696 7919000 Percutaneous transluminal mitral valvotomy

53756 Gyu1000 [X]Other mitral valve diseases

53804 7911z00 Plastic repair of aortic valve NOS

53920 7N40z00 [SO]Valve of heart NEC

53959 Gyu5800 [X]Other pulmonary valve disorders

55918 A932100 Syphilitic endocarditis of mitral valve

57318 7910212 Bjork-Shiley prosthetic replacement of mitral valve

57338 G544X00 Multiple valve disease, unspecified

57633 G114.00 Ruptured mitral valve cusp

59275 G14z.11 Rheumatic valvulitis, chronic NOS

60265 7919400 Percutaneous transluminal valvuloplasty

60533 7913300 Replacement of pulmonary valve NEC

60957 7910y00 Other specified plastic repair of mitral valve

61108 7914.00 Plastic repair of unspecified valve of heart

61427 7915200 Revision of plastic repair of tricuspid valve

61436 A932300 Syphilitic endocarditis of tricuspid valve

62095 7918300 Excision of vegetations of valve of heart

62361 7916z00 Open incision of valve of heart NOS

62633 SP00200 Mechanical complication of heart valve prosthesis

63499 7915y00 Other specified revision of plastic repair of valve of heart

63601 7913200 Prosthetic replacement of pulmonary valve

63710 SP00400 Infect and inflammatory reaction due to cardiac valve pros

64509 790D700 Replacement of valved cardiac conduit

66201 7912200 Prosthetic replacement of tricuspid valve

67659 7917.00 Closed incision of heart valve

67931 7916200 Open tricuspid valvotomy

68807 A932.11 Syphilitic valve disease

69189 7914100 Xenograft replacement of valve of heart NEC

70698 G13y.00 Multiple mitral and aortic valve involvement

71004 Gyu5A00 [X]Aortic valve disorders in diseases classified elsewhere

72417 7912z00 Plastic repair of tricuspid valve NOS

72523 7912y00 Other specified plastic repair of tricuspid valve

72613 G140z00 Rheumatic tricuspid valve disease NOS

72761 7914000 Allograft replacement of valve of heart NEC

73592 P651.00 Fused commissure of the mitral valve

73754 ZVu6e00 [X]Presence of other heart valve replacement

73904 7910214 Edwards prosthetic replacement of mitral valve

73967 7913y00 Other specified plastic repair of pulmonary valve

85856 7910411 Mitral valve repair NEC

85957 7919600 Percutaneous transluminal pulmonary valve replacement

89459 791D100 Pulmonary valvectomy

89579 G54z014 Insufficiency of unspecified heart valve

89936 7913100 Xenograft replacement of pulmonary valve

91802 7919500 Percutaneous transluminal pulmonary valve perfor dilation

92070 7911411 Aortic valve repair NEC

92226 791D000 Tricuspid valvectomy

92297 A932200 Syphilitic endocarditis of aortic valve

93158 7910000 Allograft replacement of mitral valve

93485 791B200 Operations on mitral subvalvar apparatus

93924 7912.12 Tricuspid valvuloplasty

93968 791D.00 Excision of valve of heart

94068 790D600 Creation valved conduit between left vent heart pulm artery

94345 7913z00 Plastic repair of pulmonary valve NOS

94521 Gyu1100 [X]Other rheumatic aortic valve diseases

95532 791C500 Aortoventriculoplasty with pulmonary valve autograft

95784 7918500 Closure of pulmonary valve

95799 7913411 Pulmonary valve repair NEC

95817 7914y00 Other specified plastic repair of unspecified valve of heart

96199 7912511 Tricuspid valve repair NEC

96247 7914500 Truncal valve repair

96316 7918400 Closure of tricuspid valve

97087 7919z00 Therapeutic transluminal operation on heart valve NOS

97908 7919y00 Therapeutic transluminal operation on heart valve OS

98173 7911600 Transluminal aortic valve implantation

98538 G542X00 Nonrheumatic tricuspid valve disorder, unspecified

98560 Gyu5500 [X]Other nonrheumatic mitral valve disorders

98580 7912000 Allograft replacement of tricuspid valve

98783 7912100 Xenograft replacement of tricuspid valve

99077 Gyu1200 [X]Other tricuspid valve diseases

99557 7912500 Tricuspid valvuloplasty NEC

99614 7917200 Closed tricuspid valvotomy

100482 7911500 Transapical aortic valve implantation

100910 Gyu5D00 [X]Multiple valve disorders/diseases CE

102236 7917y00 Other specified closed incision of valve of heart

102843 790Dy00 Other specified creation of valved cardiac conduit

103943 790D300 Creation of valved conduit between left ventricle and aorta

105159 7915z00 Revision of plastic repair of valve of heart NOS

105655 7914411 Repair of valve of heart NEC

47887 G541011 Aortic insufficiency, non-rheumatic

49355 G131.00 Mitral stenosis and aortic insufficiency

58076 P722z00 Atresia or stenosis of aorta NOS

58810 G541211 Aortic insufficiency alone, cause unspecified

61250 G131.13 Mitral stenosis and aortic incompetence

62293 791A300 Repair of supraaortic stenosis

63960 G122.00 Rheumatic aortic stenosis with insufficiency

68198 P722.00 Atresia and stenosis of aorta

94872 G133.11 Mitral and aortic insufficiency

1885 G110.00 Mitral stenosis

5058 G540000 Mitral incompetence, non-rheumatic

8274 G130.00 Mitral and aortic stenosis

11878 G133.12 Mitral and aortic regurgitation

17596 G131.14 Mitral stenosis and aortic regurgitation

21807 G111.11 Mitral incompetence – rheumatic

22837 G111.12 Mitral regurgitation - rheumatic

31727 G133.00 Mitral and aortic incompetence

31759 G132.12 Mitral incompetence and aortic stenosis

32435 G110.11 Rheumatic mitral stenosis

33262 G132.00 Mitral insufficiency and aortic stenosis

33907 G132.13 Mitral regurgitation and aortic stenosis

34240 G540100 Mitral incompetence, cause unspecified

44328 G112.13 Mitral stenosis with regurgitation

44488 G112.00 Mitral stenosis with insufficiency

49355 G131.00 Mitral stenosis and aortic insufficiency

50983 G112.12 Mitral stenosis with incompetence

51879 G111.00 Rheumatic mitral insufficiency

57091 P65..00 Congenital mitral stenosis

61250 G131.13 Mitral stenosis and aortic incompetence

61651 P66..00 Congenital mitral insufficiency

93158 7910000 Allograft replacement of mitral valve

94872 G133.11 Mitral and aortic insufficiency

1779 G542000 Tricuspid incompetence, non-rheumatic

2817 G542.00 Tricuspid valve disorders, non-rheumatic

9286 G140413 Tricuspid regurgitation, cause unspecified

12244 7N40200 [SO]Tricuspid valve

16373 G140.00 Tricuspid valve disease NEC

18475 G544200 Combined disorders of mitral, aortic and tricuspid valves

19699 G544100 Disorders of both mitral and tricuspid valves

21980 G140111 Tricuspid regurgitation – rheumatic

26153 7918100 Annuloplasty of tricuspid valve

26168 7912.00 Plastic repair of tricuspid valve

31505 G140000 Rheumatic tricuspid stenosis

32930 7912300 Replacement of tricuspid valve NEC

34869 G140412 Tricuspid incompetence, cause unspecified

35372 G542012 Tricuspid regurgitation, non-rheumatic

35724 G542100 Tricuspid stenosis, non-rheumatic

40582 G544000 Disorders of both aortic and tricuspid valves

42128 G140400 Tricuspid insufficiency, cause unspecified

42239 G140112 Tricuspid incompetence – rheumatic

43855 G542z00 Tricuspid valve disorders NOS

47014 7912.11 Replacement of tricuspid valve

49551 G140514 Tricuspid stenosis and regurgitation, cause unspecified

52271 G542200 Nonrheumatic tricuspid valve stenosis with insufficiency

53413 7918111 De Vega tricuspid annuloplasty

56029 G140300 Tricuspid stenosis, cause unspecified

59965 24D9.00 O/E - tricuspid murmur

60266 G140100 Rheumatic tricuspid insufficiency

61427 7915200 Revision of plastic repair of tricuspid valve

61436 A932300 Syphilitic endocarditis of tricuspid valve

62186 G14021Y Rheumatic tricuspid stenosis and incompetence

66201 7912200 Prosthetic replacement of tricuspid valve

67931 7916200 Open tricuspid valvotomy

69169 P611.00 Congenital tricuspid stenosis

72306 G140500 Tricuspid stenosis and insufficiency, cause unspecified

93113 G14021X Rheumatic tricuspid stenosis and regurgitation

93114 G140200 Rheumatic tricuspid stenosis and insufficiency

97738 G542011 Tricuspid insufficiency, non-rheumatic

1779 G542000 Tricuspid incompetence, non-rheumatic

2817 G542.00 Tricuspid valve disorders, non-rheumatic

9286 G140413 Tricuspid regurgitation, cause unspecified

21980 G140111 Tricuspid regurgitation – rheumatic

31505 G140000 Rheumatic tricuspid stenosis

34869 G140412 Tricuspid incompetence, cause unspecified

35372 G542012 Tricuspid regurgitation, non-rheumatic

35724 G542100 Tricuspid stenosis, non-rheumatic

42128 G140400 Tricuspid insufficiency, cause unspecified

42239 G140112 Tricuspid incompetence – rheumatic

49551 G140514 Tricuspid stenosis and regurgitation, cause unspecified

53413 7918111 De Vega tricuspid annuloplasty

56029 G140300 Tricuspid stenosis, cause unspecified

59965 24D9.00 O/E - tricuspid murmur

60266 G140100 Rheumatic tricuspid insufficiency

62186 G14021Y Rheumatic tricuspid stenosis and incompetence

72306 G140500 Tricuspid stenosis and insufficiency, cause unspecified

93113 G14021X Rheumatic tricuspid stenosis and regurgitation

93114 G140200 Rheumatic tricuspid stenosis and insufficiency

97738 G542011 Tricuspid insufficiency, non-rheumatic

6077 G543215 Pulmonary regurgitation, cause unspecified

14723 G543100 Pulmonary stenosis, non-rheumatic

15496 G543012 Pulmonary regurgitation, non-rheumatic

15640 G543011 Pulmonary insufficiency, non-rheumatic

19721 8CE6.00 Chronic obstructive pulmonary disease leaflet given

23608 G543000 Pulmonary incompetence, non-rheumatic

46836 7913.00 Plastic repair of pulmonary valve

54088 G141100 Rheumatic pulmonary insufficiency

61878 G543311 Pulmonary stenosis, cause unspecified

62207 G141000 Rheumatic pulmonary stenosis

105626 G141200 Rheumatic pulmonary stenosis and insufficiency

*Fractures*

S020 Fracture nose (closed)

S030 Fracture skull

S02 Fracture of face bones

S8363 Fracture tooth

S102 Fracture thoracic vertebra

S104 Fracture lumbar vertebra

S106 Fracture sacrum

S108 Fracture coccyx

S120 Fracture rib (closed)

S20 Fracture clavicle

S2240 Fracture elbow (closed)

S226 Fracture upper end humerus

S230 Fracture proximal radius/ulnar

S231 Fracture proximal radius/ulnar

S232 Fracture shaft radius/ulnar

S233 Fracture shaft radius/ulnar

S234 Fracture distal radius/ulna (closed)

S2341 Colles’ fracture (closed)

S2401 Fracture scaphoid (closed)

S24z Fracture carpal bome

S25 Fracture metacarpal bone

S26 Fracture phalanges hand

S30 Fracture neck of femur

S4F Fracture /dislocation knee

S32 Fracture patella

S33 Fracture tibia and ﬁ bula

S339 Fracture of ﬁ bula alone

S34 Fracture ankle

S352 Fracture tarsal bones/metatarsals (closed)

S3521 Fracture talus (closed)

S3527 Fracture metatarsal (closed)

S36 Fracture phalanges of foot

Falls

| TC...11 | Fall - accidental |
| --- | --- |
| T324100 | Fall from animal being ridden, rider of animal injured |
| T307100 | PCA-fall from pedal cycle - pedal cyclist injured |
| T307.00 | Pedal cycle accident involving fall from pedal cycle |
| TC...00 | Accidental falls |
| TCz..00 | Accidental falls NOS |
| TC4yz00 | Other fall from one level to another NOS |
| TC52.00 | Fall on same level from stumbling |
| U10..00 | [X]Falls |
| TC29.00 | Fall through roof |
| TCy..00 | Other falls |
| TC60y00 | Other fall in sport |
| U10z000 | [X]Unspecified fall, occurrence at home |
| T324.00 | Fall from animal being ridden |
| TC0..00 | Fall on or from stairs or steps |
| TCyz.00 | Other accidental fall NOS |
| TC51.00 | Fall on same level from tripping |
| T183.00 | MVTA - fall from motor vehicle while in motion |
| TC5..00 | Fall on same level from slipping, tripping or stumbling |
| TCy0.00 | Fall from bump against object |
| T04..00 | Fall in, on, or from train |
| T534.00 | Fall from aircraft |
| TC01.00 | Fall on or from stairs |
| TH03.00 | Late effects of accidental fall |
| TC01000 | Fall on stairs |
| U080000 | [X]Rider/occ inj by fall/thrown from anml/anml-drawn vh nncl |
| TC50.00 | Fall on same level from slipping |
| TC10.00 | Fall from ladder |
| U102.00 | [X]Fall involv ice-skates skis roller-skates or skateboards |
| T170.00 | MVTA - fall down stairs of motor bus while board/alighting |
| TC4y300 | Fall from tree |
| T337.00 | Fall from road vehicle NEC |
| TC2..00 | Fall from or out of building or other structure |
| TG04.00 | Accidentally struck by falling tree |
| TN7..00 | Injury ?accidental, fall from high place |
| TC01100 | Fall from stairs |
| TC4z.00 | Fall from one level to another NOS |
| U105.00 | [X]Fall involving wheelchair |
| U100.00 | [X]Fall on same level involving ice and snow |
| TG03.00 | Accidentally struck by falling stone |
| TE6y100 | Fallen on by unridden horse |
| TG06.00 | Accidentally struck - object falling from stationary vehicle |
| U110.00 | [X]Struck by thrown projected or falling object |
| U10z.00 | [X]Unspecified fall |
| TC28.00 | Fall from window |
| T163.00 | MVTA - motor vehicle out of control - driver falling asleep |
| T402.00 | Submersion and drowning due to falling from burning ship |
| T60E.00 | Fall from powered vehicle, industrial or commercial |
| TC42100 | Fall from bed |
| T201.00 | MVNTA-fall from motor-driven snow vehicle |
| T315000 | ADVA-fall from animal-drawn veh - pedestrian injured |
| TC3yz00 | Fall into other hole, unspecified |
| U101.00 | [X]Fall on same level from slipping, tripping and stumbling |
| TD07.00 | Accident due to fall from burning private dwelling |
| T336.00 | Fall on road vehicle NEC |
| U018411 | [X]Fall from pedal cycle without collision |
| TC5z.00 | Fall on same level from slipping, tripping or stumbling NOS |
| TC4..00 | Other fall from one level to another |
| TC11.00 | Fall from scaffolding |
| TC1..00 | Fall on or from ladders or scaffolding |
| T44zz00 | Fall in water transport NOS - unspecified person injured |
| U4B..00 | [X]Falling jumping/pushed from high place undeterm intent |
| TC23.00 | Fall from flagpole |
| U10A.00 | [X]Fall on and from stairs and steps |
| TG0z.00 | Accidentally struck by falling object NOS |
| TC32.00 | Accidental fall into manhole |
| U10F000 | [X]Fall from cliff, occurrence at home |
| TC40.00 | Fall from playground equipment |
| T171700 | MVTA-fall from car in street - pedestrian injured |
| T170100 | MVTA-fall down bus stairs - motor vehicle passenger injured |
| TC42000 | Fall from chair |
| U142600 | [X]Thret breth due cave-in fall erth+oth sub ind/constr area |
| T420.00 | Submersion or drowning due to fall from gangplank |
| T43..00 | Fall on stairs or ladders in water transport |
| T253.00 | MVNTA - fall from moving motor vehicle, except off-road MV |
| T335.00 | Fall in road vehicle NEC |
| TC60000 | Fall on same level from tackle in sport |
| U10A511 | [X]Fall on or from escalator |
| T171.00 | MVTA - fall from car in street while boarding/alighting |
| T307000 | PCA-fall from pedal cycle - pedestrian injured |
| T183200 | MVTA-fall from moving MV - motor cyclist injured |
| TC4y.00 | Other fall from one level to another |
| TC01z00 | Fall on or from stairs NOS |
| T253200 | MVNTA-fall from MV - motor cyclist injured |
| TC41.00 | Fall from cliff |
| TC02000 | Fall on steps |
| U10J000 | [X]Other fall on same level, occurrence at home |
| T421.00 | Submersion or drowning due to fall overboard |
| TC3..00 | Fall into hole or other opening in surface |
| U100300 | [X]Fall same levl involv ice/snow, occ sport/athlet area |
| U10D300 | [X]Fall frm out/thro bldng/struct occ at sport/athlet area |
| U106000 | [X]Fall involving bed, occurrence at home |
| TC4y100 | Fall from haystack |
| TD17.00 | Accident due to fall from other burning structure/building |
| TC02.00 | Fall on or from steps |
| T315.11 | Fall from animal-drawn vehicle |
| T211.00 | MVNTA-fall from other off-road motor vehicle |
| T337y00 | Fall from road vehicle NEC - other specified person injured |
| TG30800 | Accident caused by fall from moving part of machinery |
| T440.00 | Fall from one level to another NEC in water transport |
| T404.00 | Submersion/drowning due to falling from crushed watercraft |
| U10z100 | [X]Unspecified fall, occurrence in residential institution |
| TP43.00 | War injury due to crushing by falling aeroplane |
| T0x0.00 | Hit by object falling in train |
| U110600 | [X]Strck by thrwn projectd/fallng obj occ indust/constr area |
| U10J.00 | [X]Other fall on same level |
| TC32000 | Accidental fall into manhole, unspecified |
| U101000 | [X]Fall same levl frm slip trip + stumb, occurrence at home |
| U101300 | [X]Fall sme levl frm slip trip+stumb, occ sport/athlet area |
| U101100 | [X]Fall same level from slip trip + stumb occ resid instit |
| U101y00 | [X]Fall same level, slip trip+stumb, occ other specif place |
| U10Hz00 | [X]Othr fall frm one level to anothr occurrn at unspec plce |
| TG0..00 | Accidentally struck by falling object |
| T413.00 | Fall due to collision or other accident to watercraft |
| U102300 | [X]Fall inv ice-skt ski rol-skt/skbrd occ sport/athlet area |
| U10D.00 | [X]Fall from, out of or through building or structure |
| U131600 | [X]Drown+subm foll fall into bth-tub occ indust/constr area |
| U107.00 | [X]Fall involving chair |
| U10A500 | [X]Fall on + from stair + step occurrn at trade/servce area |
| TC2z.00 | Fall from or out of building or other structure NOS |
| U10H000 | [X]Other fall from one level to another, occurrence at home |
| U104100 | [X]Fall whle carried/supported oth persons occ resid instit |
| T534000 | Fall from aircraft - occupant of spacecraft injured |
| T402600 | Fall from burning ship - docker or stevedore injured |
| T404600 | Fall from crushed watercraft - docker or stevedore injured |
| U103.00 | [X]Oth fall same levl due collisn/pushing by another person |
| U106.00 | [X]Fall involving bed |
| U10D000 | [X]Fall from out of/through building/structur occurn home |
| U104000 | [X]Fall while carried/supported by other persons, occ home |
| U10A000 | [X]Fall on and from stairs and steps, occurrence at home |
| U101400 | [X]Fall same level from slip trip+stumb, occ street/highway |
| U10A100 | [X]Fall on + from stair + step occurrnce resident instit'n |
| TC02100 | Fall from steps |
| U110y00 | [X]Struck by thrwn projectd/fallng obj occ oth specif place |
| TC6z.00 | Fall on same level- push/shove/collide - other person NOS |
| T183600 | MVTA-fall from moving MV - pedal cyclist injured |
| T44zy00 | Fall in water transport NOS - other specified person injured |
| U10z600 | [X]Unspecified fall occurrn at industrial/construction area |
| TC00.00 | Fall on or from escalator |
| TC20.00 | Fall from balcony |
| U082500 | [X]Occupant of streetcar injured by fall in streetcar |
| TC60.00 | Fall on same level from sports contact |
| TC6y100 | Fall on same level from pushing by other person, unspecified |
| U10z300 | [X]Unspecified fall, occurrence at sports / athletics area |
| U107600 | [X]Fall involving chair occurrence at indust/construct area |
| T43z.00 | Fall on stairs or ladders in water transport, NOS |
| U108.00 | [X]Fall involving other furniture |
| TC6..00 | Fall on same level- collision/push/shove by/with oth person |
| T613.00 | Accident involving fall from cable car, not on rails |
| U109.00 | [X]Fall involving playground equipment |
| TC31.00 | Accidental fall into well |
| T308.00 | Pedal cycle accident hit by falling/thrown object |
| TC00000 | Fall on escalator |
| U10B.00 | [X]Fall on/from ladder |
| U107y00 | [X]Fall involving chair occurrence at other specified place |
| TC3y600 | Fall into tank |
| TG05.00 | Accidentally struck by object falling from still machine |
| U10C.00 | [X]Fall on and from scaffolding |
| T414.00 | Hit by falling object due to accident to watercraft |
| TC22.00 | Fall from building |
| T0x3y00 | Train hit by falling object - other specified person injured |
| T171200 | MVTA-fall from car in street - motor cyclist injured |
| U103300 | [X]Oth fall sme levl col/push anoth pers occ sport/athl area |
| TC21.00 | Fall from bridge |
| U142.00 | [X]Threat to breathng due cave-in fallng earth + oth substn |
| TF33z00 | Accidental mechanical suffocation by falling earth etc NOS |
| T315100 | ADVA-fall from animal-drawn veh - rider of animal injured |
| T040.00 | Fall in train |
| 8HTl.00 | Referral to elderly falls prevention clinic |
| TC3y200 | Fall into hole |
| T011.00 | Train collision with fallen tree on railway |
| T0x3.00 | Train hit by falling object |
| TC42.00 | Fall from chair or bed |
| U110000 | [X]Struck by thrown projectd/falling object occurrn at home |
| U110300 | [X]Strck by thrwn proj/fallng obj occ sport/athletics area |
| T042.00 | Fall from train |
| U131000 | [X]Drowning+submersn follow fall into bath-tub occ at home |
| T041.00 | Fall on train |
| TC42z00 | Fall from chair or bed NOS |
| U081500 | [X]Occup rail train/rail veh inj by fall in rail train/veh |
| U101200 | [X]Fall sme levl slp trp+stmb occ sch, oth inst/pub adm area |
| U100400 | [X]Fall same levl inv ice and snow, occ street / highway |
| T53..00 | Fall in, on, or from aircraft |
| U109200 | [X]Fall inv playgrnd equip occ sch oth inst/pub admin area |
| U10zz00 | [X]Unspecified fall, occurrence at unspecified place |
| T335100 | Fall in road vehicle NEC - occupant of tram injured |
| U101700 | [X]Fall same level from slip trip+stumbling, occur on farm |
| U10J300 | [X]Other fall on same level occurrn at sports/athletic area |
| U103y00 | [X]Oth fall sme levl coll/push anoth per occ oth spec place |
| U135.00 | [X]Drowning + submersion following fall into natural water |
| T324y00 | Fall from animal being ridden, other specified person inj |
| U102z00 | [X]Fall inv ice-skat ski roll-skat/skbrd occ unspecif place |
| TG00.00 | Accidentally struck by falling building |
| TC6y000 | Fall on same level from collision with other person, unspec |
| T315z00 | ADVA-fall from animal-drawn veh - unspecified person injured |
| TD18100 | Hit by object falling from burning church |
| TC3y300 | Fall into pit |
| U106200 | [X]Fall involv bed occurrn school oth instit/pub admin area |
| U100200 | [X]Fall sam lvl inv ice/snw occ sch oth inst/pub admin area |
| TF33.00 | Accidental mechanical suffocation by falling earth etc |
| TC6y200 | Fall on same level from shoving by other person, unspecified |
| U10Az00 | [X]Fall on + from stair + step occurrnce at unspecif place |
| TC3z.00 | Fall into hole NOS |
| U107200 | [X]Fall invlv chair occ at school oth instit/pub admin area |
| TC0z.00 | Fall on or from stairs or steps NOS |
| TC02z00 | Fall on or from steps NOS |
| T171100 | MVTA-fall from car in street - motor vehicle passenger inj |
| TC27.00 | Fall from wall |
| T240100 | MVNTA-fall-boarding/alighting - motor vehicle passenger inj |
| TC3y400 | Fall into quarry |
| T477000 | Hit boat after fall from boat- occ small unpowered boat inj |
| U4C..00 | [X]Falling lying running befor/into moving obj undet intent |
| TC60z00 | Fall on same level from sports contact NOS |
| U110400 | [X]Struck by thrwn projectd/fallng obj occurrn street/h'way |
| U110700 | [X]Struck by thrown projectd/falling object occurrn on farm |
| TN70.00 | Injury ?accidental, fall from residential premises |
| T44..00 | Other falls in water transport (WT) |
| U082600 | [X]Occupant of streetcar injured by fall from streetcar |
| U108100 | [X]Fall involv other furniture occurrn resident institut'n |
| TC3y.00 | Fall into other hole or other opening in surface |
| U103000 | [X]Oth fall same levl, collisn/push by anoth pers, occ home |
| U10H300 | [X]Othr fall from one level to anothr occ sport/athlet area |
| U104.00 | [X]Fall while being carried or supported by other persons |
| T53z000 | Aircraft fall NOS - occupant of spacecraft injured |
| TG30700 | Accident caused by fall into moving part of machinery |
| U104600 | [X]Fall whle carr'd/supprtd oth pers occ indust/constr area |
| T183000 | MVTA-fall from moving MV - motor vehicle driver injured |
| U105100 | [X]Fall involvng wheelchair occurrence residential instit'n |
| T335y00 | Fall in road vehicle NEC - other specified person injured |
| U109000 | [X]Fall involving playground equipment, occurrence at home |
| U10Ez00 | [X]Fall from tree, occurrence at unspecified place |
| U10E000 | [X]Fall from tree, occurrence at home |
| U100000 | [X]Fall on same level involving ice and snow occurrn home |
| U109z00 | [X]Fall involv playgrnd equipm occurrnce at unspecif place |
| U10H400 | [X]Othr fall from one level to anothr occurrn street/h'way |
| U102000 | [X]Fall inv ice-skate skis roll-skate/skateboard, occ home |
| U101z00 | [X]Fall same levl frm slip trip+stumbling, occ unspec place |
| U108000 | [X]Fall involving other furniture, occurrence at home |
| U107000 | [X]Fall involving chair, occurrence at home |
| U10H500 | [X]Other fall frm one level to anothr occ at trde/serv area |
| U10J600 | [X]Other fall on same levl, occurrn at indust/constuct area |
| U10H200 | [X]Othr fall frm one level to anothr, sch inst/pub adm area |
| U10Ay00 | [X]Fall on + from stair + step occurrn at oth specif place |
| U101500 | [X]Fall sme lvl frm slip trip+stumb, occ trade/service area |
| U107z00 | [X]Fall involving chair, occurrence at unspecified place |
| U10Bz00 | [X]Fall on and from ladder, occurrence at unspecified place |
| U10F300 | [X]Fall from cliff, occurrence at sports and athletics area |
| T431z00 | Fall-ladder water transport - unspecified person injured |
| U101600 | [X]Fall same levl, slip trip+stumb occ indust/construct area |
| U081600 | [X]Occup rail train/rail veh inj by fall from rail train/veh |
| TC4y200 | Fall from stationary vehicle |
| U142400 | [X]Threat breath due cave-in fall earth+oth subs strt/h'way |
| U10E.00 | [X]Fall from tree |
| TN7z.00 | Injury ?accidental, fall from high place NOS |
| U106100 | [X]Fall involving bed occurrence in residential institution |
| T315y00 | ADVA-fall from animal-drawn veh - other specified person inj |
| T315.00 | ADVA involving fall from animal-drawn vehicle |
| U10B600 | [X]Fall on + from ladder occurrn at industr/constructn area |
| T183y00 | MVTA-fall from moving MV - other specified person injured |
| TN71.00 | Injury ?accidental, fall from other man-made structure |
| TC3y100 | Fall into dock |
| T307z00 | PCA-fall from pedal cycle - unspecified person injured |
| T534600 | Fall from aircraft - occupant unpowered aircraft injured |
| U102y00 | [X]Fall inv ice-skt ski roll-skt/skbrd, occ oth spec place |
| T416.00 | Struck by boat/part boat after fall/jump from damaged boat |
| T170y00 | MVTA-fall down bus stairs - other specified person injured |
| T430.00 | Fall on stairs in water transport (WT) |
| T43z000 | Fall-stairs/ladders-WT NOS - occ small unpowered boat inj |
| U100z00 | [X]Fall same levl inv ice / snow, occ at unspecified place |
| T430300 | Fall-stairs water transport - passenger other watercraft inj |
| U102400 | [X]Fall inv ice-skat ski roll-skat/skbrd occ street/highway |
| U10D600 | [X]Fall from out/thro buildng/struct occ indust/constr area |
| T532.00 | Fall in aircraft |
| T440z00 | Fall to other level NEC in WT - unspecified person injured |
| T430z00 | Fall-stairs water transport - unspecified person injured |
| T042100 | Fall from train, passenger injured |
| T307y00 | PCA-fall from pedal cycle - other specified person injured |
| T532300 | Fall in aircraft - other occ comm aircraft surf/surf injured |
| T431.00 | Fall on ladder in water transport |
| U10Hy00 | [X]Other fall frm one levl to anothr occ at oth specif plce |
| U10J100 | [X]Other fall on same level, occurrnce in resident instit'n |
| T421000 | Submersion-fall overboard - occ small unpowered boat inj |
| TN72.00 | Injury ?accidental, fall from natural site |
| T43zz00 | Fall-stairs/ladders-WT NOS - unspecified person injured |
| U10B000 | [X]Fall on and from ladder, occurrence at home |
| U10By00 | [X]Fall on+from ladder, occurrence at other specified place |
| TC6yz00 | Other fall on same level- push/shove/collide-oth person NOS |
| TD07500 | Accident due to fall from burning house |
| T170600 | MVTA-fall down bus stairs - pedal cyclist injured |
| T324200 | Fall from animal being ridden, occupant of tram injured |
| T533200 | Fall on aircraft - crew commercial aircraft surface/surf inj |
| T534100 | Fall from aircraft - occupant of military aircraft injured |
| T404400 | Fall from crushed watercraft - water skier injured |
| T533100 | Fall on aircraft - occupant of military aircraft injured |
| T170400 | MVTA-fall down bus stairs - occupant of tram injured |
| T170500 | MVTA-fall down bus stairs - occ animal-drawn veh/rider inj |
| T404200 | Fall from crushed watercraft - crew other watercraft injured |
| T189100 | MVTA-obj falling on mov MV - motor vehicle passenger injured |
| T152100 | MVTA+collision+fallen object - motor vehicle passenger inj |
| T404100 | Fall from crushed watercraft - occ small powered boat inj |
| U105500 | [X]Fall involvng wheelchair occurrnce at trade/service area |
| T402500 | Fall from burning ship - swimmer injured |
| U10C600 | [X]Fall on+from scaffold occurrn at industr/constructn area |
| U135200 | [X]Drown+subm foll fall into natrl watr sch ins/pub adm area |
| U102700 | [X]Fall inv ice-skat ski roll-skat/skatebrd, occur on farm |
| T253600 | MVNTA-fall from MV - pedal cyclist injured |
| U131.00 | [X]Drowning and submersion following fall into bath-tub |
| U10H.00 | [X]Other fall from one level to another |
| U142200 | [X]Thret breth cav-in fall erth+oth sub sch ins/pub adm area |
| U108600 | [X]Fall involv oth furnitre occurrnce at indust/constr area |
| U100500 | [X]Fall same levl inv ice / snow, occ trade / service area |
| T440y00 | Fall to other level NEC in WT - other specified person inj |
| U103z00 | [X]Oth fall same levl coll/push anoth pers occ unspec place |
| T253y00 | MVNTA-fall from MV - other specified person injured |
| T533.00 | Fall on aircraft |
| U110z00 | [X]Struck by thrwn projectd/fallng obj occ unspecif place |
| U10Cz00 | [X]Fall on + from scaffold, occurrence at unspecified place |
| T040100 | Fall in train, passenger injured |
| T335000 | Fall in road vehicle NEC - pedestrian injured |
| U10H600 | [X]Other fall frm one level to anoth occ indust/constr area |
| TC1z.00 | Fall from ladder or scaffolding NOS |
| T253z00 | MVNTA-fall from MV - unspecified person injured |
| U10zy00 | [X]Unspecified fall, occurrence at other specified place |
| T240400 | MVNTA-fall-boarding/alighting - occupant of tram injured |
| TG30B00 | Accident caused by object falling from/on/moved by,machinery |
| U10z400 | [X]Unspecified fall, occurrence on street and highway |
| T04z.00 | Fall in, on or from train NOS |
| TC6y.00 | Fall on same level from other push/shove/collide oth person |
| U10Dz00 | [X]Fall from out/thro buildng/struct occurrn unspecif place |
| TC26.00 | Fall from viaduct |
| TC25.00 | Fall from turret |
| T421100 | Submersion-fall overboard - occupant small powered boat inj |
| T0x0100 | Hit by object falling in train - passenger |
| U105000 | [X]Fall involving wheelchair, occurrence at home |
| U105700 | [X]Fall involving wheelchair, occurrence on farm |
| TD08400 | Hit by object falling from burning farmhouse |
| U10Jz00 | [X]Other fall on same level occurrence at unspecified place |
| U10F100 | [X]Fall from cliff, occurrence in residential institution |
| T43zy00 | Fall-stairs/ladders-WT NOS - other specified person injured |
| T324z00 | Fall from animal being ridden, unspecified person injured |
| T431y00 | Fall-ladder water transport - other specified person injured |
| T335z00 | Fall in road vehicle NEC - unspecified person injured |
| U10A400 | [X]Fall on + from stairs + steps occurrn on street/highway |
| TG01.00 | Accidentally struck by falling rock |
| TC4y000 | Fall from embankment |
| T475.00 | Crushed by falling object on ship or while loading/unloading |
| U108z00 | [X]Fall involv oth furniture, occurrnce at unspecif place |
| T413y00 | Fall due to watercraft accid - other specified person inj |
| U10J200 | [X]Other fall on same levl occ schl oth inst/pub admin area |
| U4Bz.00 | [X]Fall jump/push frm high plce undt intnt occ unspecif plce |
| T420000 | Submersion-fall from gangplank- occ small unpowered boat inj |
| T44z400 | Fall in water transport NOS - water skier injured |
| U10D100 | [X]Fall from out of/thro buildng/struct occ resid instit'n |
| TC30500 | Accident caused by fall into swimming pool |
| U10J400 | [X]Other fall on same level, occurrence on street / highway |
| U10Jy00 | [X]Other fall on same level occurrn at oth specified place |
| T337100 | Fall from road vehicle NEC - occupant of tram injured |
| T477z00 | Hit boat after fall from boat- unspecified person injured |
| TD08300 | Hit by object falling from burning caravan |
| T211200 | MVNTA-fall from other vehicle - motor cyclist injured |
| U110200 | [X]Strck by thr proj/fallng obj occ sch oth ins/pub adm area |
| T402400 | Fall from burning ship - water skier injured |
| U4B6.00 | [X]Fall jump/push frm high plce undt intn indust/constr area |
| T240.00 | MVNTA - fall while boarding or alighting from motor vehicle |
| T253000 | MVNTA-fall from MV - motor vehicle driver injured |
| T152000 | MVTA+collision+fallen object - motor vehicle driver injured |
| T477.00 | Hit by boat, or part thereof, after fall from boat |
| TC3y000 | Fall into cavity, unspecified |
| T170300 | MVTA-fall down bus stairs - motor cycle passenger injured |
| U135z00 | [X]Drown+subm foll fall into naturl watr occ unspecif place |
| T152.00 | MVTA involving collision between motor vehicle+fallen object |
| U105z00 | [X]Fall involving wheelchair occurrnce at unspecified place |
| TD07300 | Accident due to fall from burning caravan |
| T430y00 | Fall-stairs water transport - other specified person injured |
| U109300 | [X]Fall involv playgrnd equipm occurrn at sport/athlet area |
| T324000 | Fall from animal being ridden, pedestrian injured |
| TC32100 | Accidental fall into storm drain |
| U10Ey00 | [X]Fall from tree, occurrence at other specified place |

Body Mass Index

| 22A..00 | O/E - weight |
| --- | --- |
| 22A1.00 | O/E - weight > 20% below ideal |
| 22A2.00 | O/E -weight 10-20% below ideal |
| 22A3.00 | O/E - weight within 10% ideal |
| 22A4.00 | O/E - weight 10-20% over ideal |
| 22A4.11 | O/E - overweight |
| 22A5.00 | O/E - weight > 20% over ideal |
| 22A5.11 | O/E - obese |
| 22A6.00 | O/E - Underweight |
| 22K..00 | Body Mass Index |
| 22K1.00 | Body Mass Index normal K/M2 |
| 22K2.00 | Body Mass Index high K/M2 |
| 22K3.00 | Body Mass Index low K/M2 |
| 22K4.00 | Body mass index index 25-29 - overweight |
| 22K5.00 | Body mass index 30+ - obesity |
| 22K6.00 | Body mass index less than 20 |
| 22K7.00 | Body mass index 40+ - severely obese |
| 22K8.00 | Body mass index 20-24 - normal |
| 22Z..00 | Height and Weight |

*Antihypertensive medication*

Blood pressure lowering medications will be identified from the therapy file using product codes. These include all medications from the BNF chapters:

2.2.1 – Thiazide diuretics, with product codes:

| 2 | Bendroflumethiazide 2.5mg tablets |
| --- | --- |
| 58 | Bendroflumethiazide 5mg tablets |
| 542 | Hydrochlorothiazide 25mg tablets |
| 605 | Chlortalidone 50mg tablets |
| 1125 | Navidrex -k Tablet (Novartis Pharmaceuticals UK Ltd) |
| 1170 | Cyclopenthiazide 500microgram tablets |
| 1209 | Neo-Naclex 5mg tablets (Mercury Pharma Group Ltd) |
| 1211 | Bendroflumethiazide 2.5mg / Potassium chloride 630mg (potassium 8.4mmol) modified-release tablets |
| 1213 | Neo-Naclex-K modified-release tablets (Mercury Pharma Group Ltd) |
| 2046 | Navidrex 500microgram tablets (Mercury Pharma Group Ltd) |
| 2612 | Indapamide 2.5mg tablets |
| 2833 | CYCLOPENTHIAZIDE -K tablets |
| 3056 | Natrilix SR 1.5mg tablets (Servier Laboratories Ltd) |
| 3517 | Hydrochlorothiazide 50mg tablets |
| 3997 | Hygroton 50mg tablets (Alliance Pharmaceuticals Ltd) |
| 4044 | Diurexan 20mg tablets (Meda Pharmaceuticals Ltd) |
| 4332 | Metolazone 5mg tablets |
| 4334 | Metolazone 500microgram low dose Tablet |
| 5112 | Indapamide 1.5mg modified-release tablets |
| 6816 | Chlorothiazide 250mg/5ml oral suspension |
| 7351 | Bendroflumethiazide 2.5mg/5ml oral suspension |
| 7618 | Xipamide 20mg tablets |
| 7641 | Natrilix 2.5mg tablets (Servier Laboratories Ltd) |
| 7698 | Aprinox 5mg tablets (Amdipharm Plc) |
| 8526 | Aprinox 2.5mg tablets (Amdipharm Plc) |
| 8602 | Metenix 5mg tablets (Sanofi) |
| 8836 | Chlorothiazide 500mg tablets |
| 8891 | Hygroton -k Tablet (Novartis Pharmaceuticals UK Ltd) |
| 12110 | Hydroflumethiazide 50mg Tablet |
| 12360 | Nephril 1mg Tablet (Pfizer Ltd) |
| 13246 | Chlorothiazide 150mg/5ml oral suspension |
| 13363 | Esidrex 50mg Tablet (Novartis Pharmaceuticals UK Ltd) |
| 13525 | Hydrenox 50mg Tablet (Knoll Ltd) |
| 15457 | Baycaron 25mg Tablet (Bayer Plc) |
| 17143 | Mefruside 25mg Tablet |
| 17252 | Esidrex 25mg Tablet (Novartis Pharmaceuticals UK Ltd) |
| 17720 | Saluric 500mg Tablet (Merck Sharp & Dohme Ltd) |
| 18267 | Enduron 5mg Tablet (Abbott Laboratories Ltd) |
| 18973 | Centyl 2.5mg Tablet (Edwin Burgess Ltd) |
| 19352 | Xuret 0.5mg Tablet (Galen Ltd) |
| 20057 | Methyclothiazide 5mg Tablet |
| 21803 | Berkozide 2.5mg Tablet (Berk Pharmaceuticals Ltd) |
| 21867 | Berkozide 5mg Tablet (Berk Pharmaceuticals Ltd) |
| 23427 | Bendroflumethiazide 5mg tablets (A A H Pharmaceuticals Ltd) |
| 24189 | Neo-bendromax 2.5mg Tablet (Ashbourne Pharmaceuticals Ltd) |
| 24190 | Neo-bendromax 5mg Tablet (Ashbourne Pharmaceuticals Ltd) |
| 26256 | Opumide 2.5mg Tablet (Opus Pharmaceuticals Ltd) |
| 26275 | Nindaxa 2.5 tablets (Ashbourne Pharmaceuticals Ltd) |
| 27256 | Bendroflumethiazide 2.5mg tablets (Wockhardt UK Ltd) |
| 27689 | Bendroflumethiazide 2.5mg tablets (IVAX Pharmaceuticals UK Ltd) |
| 27957 | Natramid 2.5mg Tablet (Trinity Pharmaceuticals Ltd) |
| 29991 | Centyl 5mg Tablet (Edwin Burgess Ltd) |
| 31670 | Bendroflumethiazide 2.5mg tablets (Teva UK Ltd) |
| 31820 | Bendroflumethiazide 5mg tablets (Wockhardt UK Ltd) |
| 33083 | Indapamide 2.5mg tablets (Teva UK Ltd) |
| 33415 | Bendroflumethiazide 2.5mg tablets (Generics (UK) Ltd) |
| 33651 | Bendroflumethiazide 2.5mg tablets (A A H Pharmaceuticals Ltd) |
| 33724 | Diuril 250mg/5ml oral suspension (Imported (United States)) |
| 34059 | Bendroflumethiazide 2.5mg tablets (Actavis UK Ltd) |
| 34124 | Bendroflumethiazide 5mg tablets (Actavis UK Ltd) |
| 34551 | Indapamide 2.5mg tablets (Generics (UK) Ltd) |
| 34602 | Bendroflumethiazide 2.5mg tablets (Sovereign Medical Ltd) |
| 34803 | Bendroflumethiazide 2.5mg Tablet (Regent Laboratories Ltd) |
| 39447 | Varbim XL 1.5mg tablets (Teva UK Ltd) |
| 40149 | Bendroflumethiazide 5mg tablets (IVAX Pharmaceuticals UK Ltd) |
| 40886 | Bendroflumethiazide 2.5mg tablets (Almus Pharmaceuticals Ltd) |
| 40907 | Indapamide 2.5mg tablets (Genus Pharmaceuticals Ltd) |
| 41517 | Bendroflumethiazide 5mg tablets (Teva UK Ltd) |
| 41861 | Tensaid XL 1.5mg tablets (Generics (UK) Ltd) |
| 41885 | Ethibide XL 1.5mg tablets (Genus Pharmaceuticals Ltd) |
| 42906 | Indapamide 2.5mg tablets (Niche Generics Ltd) |
| 43184 | Mapemid XL 1.5mg tablets (Teva UK Ltd) |
| 43516 | Indapamide 2.5mg tablets (Actavis UK Ltd) |
| 44168 | Indipam XL 1.5mg tablets (Actavis UK Ltd) |
| 46302 | Neo-Naclex 2.5mg tablets (Mercury Pharma Group Ltd) |
| 46675 | Indapamide 1.5mg modified-release tablets (A A H Pharmaceuticals Ltd) |
| 47844 | Bendroflumethiazide 2.5mg tablets (Kent Pharmaceuticals Ltd) |
| 48079 | Indapamide 2.5mg tablets (Zentiva) |
| 48099 | Indapamide 2.5mg tablets (A A H Pharmaceuticals Ltd) |
| 48132 | Hydrochlorothiazide Capsule |
| 49529 | Indapamide 2.5mg tablets (Phoenix Healthcare Distribution Ltd) |
| 49752 | Metolazone 2.5mg/5ml oral solution |
| 5189 | Enalapril 20mg / Hydrochlorothiazide 12.5mg tablets |
| 32166 | Capto-co 25mg+50mg Tablet (IVAX Pharmaceuticals UK Ltd) |
| 37978 | Perindopril arginine 5mg / Indapamide 1.25mg tablets |
| 50607 | Perindopril arginine 2mg with Indapamide 625 micrograms tablet |
| 51258 | Coversyl Arginine Plus 5mg/1.25mg tablets (Doncaster Pharmaceuticals Ltd) |
| 18200 | Olmesartan medoxomil 20mg / Hydrochlorothiazide 12.5mg tablets |
| 24268 | Hydrochlorothiazide with valsartan 12.5mg with 80mg Tablet |
| 24632 | Hydrochlorothiazide with losartan 25mg with 100mg Tablet |
| 35196 | CoAprovel 300mg/25mg tablets (Sanofi) |
| 35380 | Hydrochlorothiazide with olmesartan medoxomil 12.5mg with 20mg tablet |
| 38367 | Hydrochlorothiazide with losartan 12.5mg with 100mg Tablet |
| 43915 | Olmetec Plus 40mg/12.5mg tablets (Daiichi Sankyo UK Ltd) |
| 46687 | Olmesartan medoxomil with amlodipine and hydrochlorothiazide 20mg + 5mg + 12.5mg Tablet |
| 47467 | Olmesartan medoxomil with amlodipine and hydrochlorothiazide 40mg + 5mg + 25mg Tablet |
| 8303 | Tenavoid Tablet (Edwin Burgess Ltd) |
| 8464 | Meprobamate with bendroflumethiazide Tablet |
| 581 | Atenolol 50mg with Chlortalidone 12.5mg tablets |
| 1788 | Atenolol 100mg with Chlortalidone 25mg tablets |
| 5721 | Co-tenidone 100mg/25mg tablets |
| 8147 | Lopresoretic Tablet (Novartis Pharmaceuticals UK Ltd) |
| 8673 | Oxprenolol with cyclopenthiazide 160mg+0.25mg Modified-release tablet |
| 9783 | Co-tenidone 50mg/12.5mg tablets |
| 11338 | Bendroflumethiazide 5mg with Nadolol 40mg tablets |
| 15488 | Metoprolol tartrate with chlortalidone Tablet |
| 19055 | Chlortalidone 12.5mg with Atenolol 50mg tablets |
| 19142 | Bendroflumethiazide 2.5mg with Timolol maleate 10mg tablets |
| 21025 | Prestim forte Tablet (LEO Pharma) |
| 23131 | Bendroflumethiazide 5mg with Propanolol 160mg modified-release capsules |
| 24280 | Totaretic 100mg+25mg Tablet (C P Pharmaceuticals Ltd) |
| 28177 | Hydrochlorothiazide with atenolol and amiloride Capsule |
| 29427 | Hydrochlorothiazide with metoprolol tartrate 12.5mg with 100mg tablet |
| 30519 | Amiloride with timolol with hydrochlorothiazide tablets |
| 31708 | Co-tenidone 50mg/12.5mg tablets (Actavis UK Ltd) |
| 32094 | Co-tenidone 50mg/12.5mg tablets (A A H Pharmaceuticals Ltd) |
| 34034 | Co-tenidone 50mg/12.5mg tablets (IVAX Pharmaceuticals UK Ltd) |
| 34825 | Co-tenidone 50mg/12.5mg tablets (Teva UK Ltd) |
| 37725 | Co-tenidone 100mg/25mg tablets (Generics (UK) Ltd) |
| 52145 | Cyclopenthiazide 0.25mg with oxprenolol 160mg modified-release tablets |
| 23505 | Adizem xl plus 150mg+12.5mg Modified-release capsule (Napp Pharmaceuticals Ltd) |
| 6794 | Perindopril erbumine 4mg / Indapamide 1.25mg tablets |
| 3054 | Hygroton 100mg Tablet (Alliance Pharmaceuticals Ltd) |
| 348 | Moduretic Tablet (Bristol-Myers Squibb Pharmaceuticals Ltd) |
| 2002 | Amiloride 5mg / hydrochlorothiazide 50mg tablets |
| 3701 | Amiloride 2.5mg / hydrochlorothiazide 25mg tablets |
| 4034 | Amiloride 5mg / hydrochlorothiazide 50mg/5ml solution |
| 7961 | Spironolactone 50mg with hydroflumethiazide 50mg tablet |
| 8058 | Normetic Tablet (Abbott Laboratories Ltd) |
| 8521 | Spironolactone 25mg with hydroflumethiazide 25mg tablet |
| 9223 | Triamterene with hydrochlorothiazide 50mg + 25mg Tablet |
| 15127 | Hydrochlorothiazide with triamterene 25mgwith50mg Tablet |
| 18733 | Co-amilozide 5mg with 50mg/ml oral solution |
| 19890 | Hydrochlorothiazide with amiloride 25mgwith2.5mg Tablet |
| 25505 | Spiro-co 50mg+50mg Tablet (IVAX Pharmaceuticals UK Ltd) |
| 26220 | Delvas Tablet (Berk Pharmaceuticals Ltd) |
| 29529 | Hydroflumethiazide with spironolactone 25mg+25mg Tablet |
| 37294 | Triamterene with chlortalidone 50mg + 25mg Tablet |

2.2.3 – Potassium sparing diuretics and aldosterone receptor antagonists:

| 692 | Spironolactone 25mg tablets |
| --- | --- |
| 708 | Spironolactone 50mg tablets |
| 787 | Spironolactone 100mg capsule |
| 1060 | Amiloride 5mg tablets |
| 2142 | Spironolactone 100mg tablets |
| 2179 | Triamterene 50mg capsules |
| 2389 | Aldactone 25mg tablets (Pfizer Ltd) |
| 4068 | Dytac 50mg capsules (Mercury Pharma Group Ltd) |
| 4161 | Spiroctan 25mg Tablet (Roche Products Ltd) |
| 4960 | Aldactone 50mg tablets (Pfizer Ltd) |
| 6815 | Spironolactone 50mg/5ml oral suspension sugar free |
| 7952 | Aldactone 100mg tablets (Pfizer Ltd) |
| 7991 | Spiroctan 100mg Capsule (Roche Products Ltd) |
| 9935 | Amiloride 5mg/5ml oral solution sugar free |
| 10214 | Spironolactone 5mg/5ml oral suspension sugar free |
| 10251 | Eplerenone 25mg tablets |
| 11156 | Spirolone 25mg Tablet (Berk Pharmaceuticals Ltd) |
| 11519 | Spironolactone 25mg/5ml oral suspension sugar free |
| 12946 | Spironolactone 10mg/5ml oral suspension sugar free |
| 13264 | Spironolactone 15mg/5ml oral suspension |
| 13352 | Midamor 5mg Tablet (MSD Thomas Morson Pharmaceuticals) |
| 14109 | Spironolactone 100mg/5ml oral solution sugar free |
| 14144 | Inspra 25mg tablets (Pfizer Ltd) |
| 15052 | Spiroctan 50mg Tablet (Roche Products Ltd) |
| 16531 | Eplerenone 50mg tablets |
| 17902 | Spirolone 100mg Tablet (Berk Pharmaceuticals Ltd) |
| 17950 | Spirolone 50mg Tablet (Berk Pharmaceuticals Ltd) |
| 19195 | Spironolactone 50mg Tablet (Wyeth Pharmaceuticals) |
| 21911 | Spirospare 25mg Tablet (Ashbourne Pharmaceuticals Ltd) |
| 23091 | Spirospare 100 tablets (Ashbourne Pharmaceuticals Ltd) |
| 24893 | Amilospare Tablet (Ashbourne Pharmaceuticals Ltd) |
| 25494 | Diatensec 50mg Tablet (Pharmacia Ltd) |
| 26217 | Berkamil 5mg Tablet (Berk Pharmaceuticals Ltd) |
| 29397 | Spiretic 100mg Tablet (DDSA Pharmaceuticals Ltd) |
| 29694 | Inspra 50mg tablets (Pfizer Ltd) |
| 31219 | Spironolactone 100mg tablets (A A H Pharmaceuticals Ltd) |
| 31375 | Amilamont 5mg/5ml oral solution sugar free (Rosemont Pharmaceuticals Ltd) |
| 31529 | Spironolactone 25mg tablets (Teva UK Ltd) |
| 32837 | Spironolactone 50mg tablets (Teva UK Ltd) |
| 33837 | Amiloride 5mg tablets (A A H Pharmaceuticals Ltd) |
| 34296 | Spironolactone 25mg tablets (A A H Pharmaceuticals Ltd) |
| 34324 | Amiloride 5mg tablets (Teva UK Ltd) |
| 34347 | Spironolactone 25mg tablets (Actavis UK Ltd) |
| 34750 | Amiloride 5mg tablets (Actavis UK Ltd) |
| 34908 | Spironolactone 25mg tablets (IVAX Pharmaceuticals UK Ltd) |
| 35789 | Spironolactone 25mg Tablet (Celltech Pharma Europe Ltd) |
| 41074 | Spironolactone 25mg tablets (Almus Pharmaceuticals Ltd) |
| 41592 | Spironolactone 100mg tablets (Actavis UK Ltd) |
| 41630 | Amiloride 5mg Tablet (IVAX Pharmaceuticals UK Ltd) |
| 41660 | Spironolactone 100mg tablets (Teva UK Ltd) |
| 41706 | Spironolactone 50mg tablets (IVAX Pharmaceuticals UK Ltd) |
| 43514 | Spironolactone 50mg tablets (A A H Pharmaceuticals Ltd) |
| 43523 | Amiloride 5mg tablets (Generics (UK) Ltd) |
| 44254 | Amiloride 5.67mg tablets |
| 45078 | Spironolactone 25mg/5ml Oral solution sugar free (Rosemont Pharmaceuticals Ltd) |
| 46674 | Spironolactone 50mg/5ml Oral suspension sugar free (Rosemont Pharmaceuticals Ltd) |
| 46930 | Amiloride 5mg tablets (Wockhardt UK Ltd) |
| 46990 | Spironolactone 50mg/5ml oral suspension |
| 47018 | Spironolactone 25mg/5ml oral suspension |
| 47687 | Spiretic 25mg Tablet (DDSA Pharmaceuticals Ltd) |
| 49388 | Spironolactone 100mg/5ml oral suspension |
| 50079 | Spironolactone 10mg/5ml oral suspension |
| 50370 | Spironolactone 5mg/5ml oral suspension |
| 51652 | Spironolactone 25mg tablets (Doncaster Pharmaceuticals Ltd) |
| 51720 | Spironolactone 25mg/5ml oral solution |
| 51933 | Spironolactone 50mg/5ml oral solution |
| 52366 | Spironolactone 5mg/5ml oral solution |
| 52970 | Spironolactone 10mg/5ml oral solution |
| 53253 | Spironolactone 50mg/5ml oral suspension (Drug Tariff Special Order) |
| 47647 | Co-amilofruse oral liquid |

2.2.4 – Potassium sparing diuretics and other diuretics, with product codes:

| 56 | Co-amilofruse 5mg/40mg tablets |
| --- | --- |
| 193 | Co-amilofruse 2.5mg/20mg tablets |
| 923 | Co-amilozide 5mg/50mg tablets |
| 924 | Co-amilozide 2.5mg/25mg tablets |
| 1251 | Moduret 25 tablets (Merck Sharp & Dohme Ltd) |
| 1297 | Aldactide 50 tablets (Pfizer Ltd) |
| 1721 | Dyazide 50mg/25mg tablets (Mercury Pharma Group Ltd) |
| 2001 | Aldactide 25 tablets (Pfizer Ltd) |
| 2255 | Navispare 2.5mg/250microgram tablets (Mercury Pharma Group Ltd) |
| 2493 | Burinex A 5mg/1mg tablets (LEO Pharma) |
| 2772 | Lasoride 5mg/40mg tablets (Sanofi) |
| 2961 | Frusene 50mg/40mg tablets (Orion Pharma (UK) Ltd) |
| 3793 | Co-amilofruse 10mg/80mg tablets |
| 4661 | Spironolactone 50mg / Furosemide 20mg capsules |
| 4873 | Fru-Co 5mg/40mg tablets (Teva UK Ltd) |
| 5416 | Co-triamterzide 50mg/25mg tablets |
| 5727 | Amiloride 2.5mg / Cyclopenthiazide 250microgram tablets |
| 7441 | Lasilactone 20mg/50mg capsules (Sanofi) |
| 8897 | Triam-Co 50mg/25mg tablets (IVAX Pharmaceuticals UK Ltd) |
| 11265 | Triamterene 50mg / Furosemide 40mg tablets |
| 11384 | Co-flumactone 50mg/50mg tablets |
| 12547 | Triamterene 50mg / Chlortalidone 50mg tablets |
| 13435 | Frumil Forte 10mg/80mg tablets (Sanofi) |
| 14587 | Amiloride 5mg / Bumetanide 1mg tablets |
| 15811 | Co-flumactone 25mg/25mg tablets |
| 16498 | Kalspare tablets (DHP Healthcare Ltd) |
| 18361 | Amilmaxco 5mg/50mg tablets (Ashbourne Pharmaceuticals Ltd) |
| 18726 | Triamaxco 50mg/25mg tablets (Ashbourne Pharmaceuticals Ltd) |
| 20066 | Amil-Co 5mg/50mg tablets (IVAX Pharmaceuticals UK Ltd) |
| 21938 | Froop Co 5mg/40mg tablets (Ashbourne Pharmaceuticals Ltd) |
| 25965 | Co-amilofruse 2.5mg/20mg tablets (Wockhardt UK Ltd) |
| 28129 | Co-amilofruse 5mg/40mg tablets (Teva UK Ltd) |
| 31150 | Co-amilozide 5mg/50mg tablets (IVAX Pharmaceuticals UK Ltd) |
| 31773 | Co-amilofruse 5mg/40mg tablets (Wockhardt UK Ltd) |
| 33527 | Co-amilofruse 5mg/40mg tablets (Generics (UK) Ltd) |
| 33658 | Co-amilofruse 5mg/40mg tablets (A A H Pharmaceuticals Ltd) |
| 34280 | Co-amilofruse 2.5mg/20mg tablets (Sandoz Ltd) |
| 34367 | Co-amilozide 2.5mg/25mg tablets (Wockhardt UK Ltd) |
| 34622 | Co-amilofruse 10mg/80mg tablets (Wockhardt UK Ltd) |
| 38901 | Frumil LS 20mg/2.5mg tablets (Sanofi) |
| 39807 | Frumil 40mg/5mg tablets (Sanofi) |
| 41533 | Co-amilofruse 2.5mg/20mg tablets (Teva UK Ltd) |
| 41556 | Co-amilozide 5mg/50mg tablets (Teva UK Ltd) |
| 41719 | Co-amilofruse 5mg/40mg tablets (Actavis UK Ltd) |
| 42142 | Moduretic 5mg/50mg tablets (Merck Sharp & Dohme Ltd) |
| 43508 | Co-amilofruse 5mg/40mg tablets (Sandoz Ltd) |
| 46916 | Co-amilozide 5mg/50mg tablets (A A H Pharmaceuticals Ltd) |
| 47804 | Co-triamterzide 50mg/25mg tablets (A A H Pharmaceuticals Ltd) |
| 53508 | Spironolactone 5mg/5ml / Chlorothiazide 50mg/5ml oral suspension |
| 1301 | Frumil ls 20mg+2.5mg Tablet (Helios Healthcare Ltd) |
| 2495 | Bumetanide with Amiloride tablets |
| 3050 | Furosemide with triamterene 40mgwith50mg Tablet |
| 4211 | Furosemide with amiloride 20mg+2.5mg Tablet |
| 9456 | Amiloride 5mg / furosemide 40mg tablets |
| 18332 | Aridil 20mg+2.5mg Tablet (C P Pharmaceuticals Ltd) |
| 18497 | Amiloride 10mg / furosemide 80mg tablets |
| 3293 | Moduretic Oral solution (Bristol-Myers Squibb Pharmaceuticals Ltd) |
| 12546 | Kalspare Tablet (Dominion Pharma) |
| 22923 | Hydrochlorothiazide with amiloride 50mg with 5mg Tablet |
| 24008 | Vasetic Tablet (Shire Pharmaceuticals Ltd) |
| 25500 | Hypertane 50 Tablet (Schwarz Pharma Ltd) |
| 26219 | Zida-co 5mg+50mg Tablet (Opus Pharmaceuticals Ltd) |
| 28157 | Kalspare ls Tablet (Dominion Pharma) |
| 30272 | Benthiazide with Triamterene capsules |
| 31131 | Spiro-co 25mg+25mg Tablet (IVAX Pharmaceuticals UK Ltd) |
| 45916 | Hydroflumethiazide with spironolactone 50mg+50mg Tablet |

2.4 - Beta-adrenoreceptor blocking drugs:

| 5 | Atenolol 50mg tablets |
| --- | --- |
| 24 | Atenolol 100mg tablets |
| 26 | Atenolol 25mg tablets |
| 197 | Atenolol 5mg/10ml solution for injection ampoules |
| 751 | Nebivolol 5mg tablets |
| 817 | Carvedilol 3.125mg tablets |
| 822 | Bisoprolol 1.5mg/5ml oral suspension |
| 1295 | Labetalol 400mg tablets |
| 1333 | Oxprenolol 40mg tablets |
| 1334 | Oxprenolol 160mg modified-release tablets |
| 1597 | Labetalol 100mg tablets |
| 1684 | Beta-Adalat modified-release capsules (Bayer Plc) |
| 2361 | Trasicor 80mg Tablet (Novartis Pharmaceuticals UK Ltd) |
| 2432 | Tenormin LS 50mg tablets (AstraZeneca UK Ltd) |
| 2499 | Nadolol 80mg tablets |
| 2587 | Tenormin 100mg tablets (AstraZeneca UK Ltd) |
| 2590 | Tenormin 25mg tablets (AstraZeneca UK Ltd) |
| 2629 | Carvedilol 12.5mg tablets |
| 2775 | Labetalol 200mg tablets |
| 2780 | Oxprenolol 80mg tablets |
| 3516 | Oxprenolol 20mg tablets |
| 3691 | Sotalol 160mg with hydrochlorothiazide 25mg tablet |
| 3748 | Oxprenolol 160mg Tablet |
| 4025 | Slow-Trasicor 160mg tablets (Amdipharm Plc) |
| 4265 | Celectol 200mg Tablet (Pantheon Healthcare Ltd) |
| 4410 | Carvedilol 6.25mg tablets |
| 4429 | Trasidrex modified-release tablets (Mercury Pharma Group Ltd) |
| 4542 | Atenolol 50mg / Nifedipine 20mg modified-release capsules |
| 4588 | Visken 5mg Tablet (Sovereign Medical Ltd) |
| 4725 | Labetalol 50mg tablets |
| 5284 | Pindolol 5mg tablets |
| 6066 | Atenolol 25mg/5ml oral solution sugar free |
| 7049 | Carvedilol 25mg tablets |
| 7429 | Tenormin 5mg/10ml solution for injection ampoules (AstraZeneca UK Ltd) |
| 7474 | Trasicor 20mg Tablet (Novartis Pharmaceuticals UK Ltd) |
| 7528 | Nebilet 5mg tablets (A Menarini Pharma UK S.R.L.) |
| 7620 | Acebutolol 400mg tablets |
| 7974 | Celiprolol 400mg tablets |
| 8023 | Sectral 400mg tablets (Sanofi) |
| 8061 | Sotalol 80mg with hydrochlorothiazide 12.5mg tablet |
| 8113 | Acebutolol 200mg capsules |
| 8172 | Acebutolol 100mg capsules |
| 8262 | Celiprolol 200mg tablets |
| 8290 | Trasicor 40mg Tablet (Novartis Pharmaceuticals UK Ltd) |
| 8555 | Sectral 200mg capsules (Sanofi) |
| 8642 | Tenif 50mg/20mg modified-release capsules (AstraZeneca UK Ltd) |
| 8707 | Trandate 200mg tablets (Focus Pharmaceuticals Ltd) |
| 8807 | Trandate 400mg tablets (Focus Pharmaceuticals Ltd) |
| 9016 | Trandate 100mg tablets (Focus Pharmaceuticals Ltd) |
| 9273 | Trandate 50mg tablets (Focus Pharmaceuticals Ltd) |
| 10191 | Atenix 50 tablets (Ashbourne Pharmaceuticals Ltd) |
| 10429 | Lopresor 50mg Tablet (Novartis Pharmaceuticals UK Ltd) |
| 10716 | Corgard 80mg tablets (Sanofi) |
| 10777 | Trasicor 160mg Tablet (Novartis Pharmaceuticals UK Ltd) |
| 12296 | Sectral 100mg capsules (Sanofi) |
| 12456 | Sotazide Tablet (Bristol-Myers Squibb Pharmaceuticals Ltd) |
| 13394 | Tenormin 25mg/5ml syrup (AstraZeneca UK Ltd) |
| 13499 | Lopresor 100mg Tablet (Novartis Pharmaceuticals UK Ltd) |
| 13871 | Co-prenozide 160mg/0.25mg modified-release tablets |
| 14117 | Eucardic 3.125mg tablets (Roche Products Ltd) |
| 14146 | Eucardic 6.25mg tablets (Roche Products Ltd) |
| 14502 | Metoprolol 5mg/5ml solution for injection ampoules |
| 14673 | Pindolol 15mg tablets |
| 15042 | Tolerzide Tablet (Bristol-Myers Squibb Pharmaceuticals Ltd) |
| 15176 | Totamol 50mg Tablet (C P Pharmaceuticals Ltd) |
| 15730 | Totamol 100mg Tablet (C P Pharmaceuticals Ltd) |
| 16645 | Labrocol 400mg Tablet (Lagap) |
| 16776 | Celectol 400mg Tablet (Pantheon Healthcare Ltd) |
| 17122 | Isoprenaline 200micrograms/1ml solution for injection ampoules |
| 17322 | Atenix 25 tablets (Ashbourne Pharmaceuticals Ltd) |
| 17783 | Spiroprop Tablet (Pharmacia Ltd) |
| 18414 | Eucardic 12.5mg tablets (Roche Products Ltd) |
| 18950 | Totamol 25mg Tablet (C P Pharmaceuticals Ltd) |
| 19172 | Atenolol 25mg tablets (IVAX Pharmaceuticals UK Ltd) |
| 19182 | Atenolol 50mg tablets (IVAX Pharmaceuticals UK Ltd) |
| 19191 | Atenolol 100mg tablets (Teva UK Ltd) |
| 19202 | Carvedilol 6.25mg tablets (Teva UK Ltd) |
| 19437 | Eucardic 25mg tablets (Roche Products Ltd) |
| 19998 | Trandate 100mg/20ml solution for injection ampoules (Focus Pharmaceuticals Ltd) |
| 20012 | Visken 15mg Tablet (Sovereign Medical Ltd) |
| 20169 | Practolol 2mg/ml injection |
| 20502 | Atenix 100 tablets (Ashbourne Pharmaceuticals Ltd) |
| 20728 | Atenamin 25mg Tablet (OPD Pharm) |
| 21133 | Atenamin 50mg Tablet (OPD Pharm) |
| 21885 | Oxyprenix SR 160mg tablets |
| 22793 | Labrocol 200mg Tablet (Lagap) |
| 24094 | Trasicor 40mg tablets (Amdipharm Plc) |
| 24191 | Antipressan 50mg tablets (Teva UK Ltd) |
| 24195 | Antipressan 100mg tablets (Teva UK Ltd) |
| 24461 | Betaloc I.V. 5mg/5ml solution for injection ampoules (AstraZeneca UK Ltd) |
| 25462 | Clopamide 5mg with Pindolol 10mg tablets |
| 25644 | Apsolox 80mg Tablet (Approved Prescription Services Ltd) |
| 26211 | Antipressan 25mg tablets (Teva UK Ltd) |
| 26922 | Brevibloc Premixed 100mg/10ml solution for injection vials (Baxter Healthcare Ltd) |
| 27357 | Oxprenolol 40mg Tablet (Actavis UK Ltd) |
| 27719 | Metoros ls 95mg Tablet (Geigy Pharmaceuticals) |
| 28700 | Cartrol 10mg Tablet (Novartis Consumer Health UK Ltd) |
| 29180 | Trasicor 80mg tablets (Amdipharm Plc) |
| 29230 | Slow-pren 160mg Tablet (IVAX Pharmaceuticals UK Ltd) |
| 29368 | Atenolol 25mg tablets (Teva UK Ltd) |
| 29398 | Atenamin 100mg Tablet (OPD Pharm) |
| 29762 | Mepranix 50mg Tablet (Ashbourne Pharmaceuticals Ltd) |
| 29827 | Carteolol HCl 10mg tablets |
| 29998 | Metoros 190mg Tablet (Novartis Pharmaceuticals UK Ltd) |
| 30400 | Mepranix 100mg Tablet (Ashbourne Pharmaceuticals Ltd) |
| 30541 | Esmolol HCl 250mg/ml concentrate solution for infusion |
| 30636 | Vasaten 50mg Tablet (Shire Pharmaceuticals Ltd) |
| 30770 | Labetalol 200mg tablets (A A H Pharmaceuticals Ltd) |
| 31536 | Atenolol 25mg tablets (Kent Pharmaceuticals Ltd) |
| 31934 | Atenolol 100mg tablets (IVAX Pharmaceuticals UK Ltd) |
| 32787 | Visken 15mg tablets (Amdipharm Plc) |
| 33079 | Atenolol 100mg tablets (Generics (UK) Ltd) |
| 33085 | Atenolol 100mg tablets (A A H Pharmaceuticals Ltd) |
| 33092 | Atenolol 50mg tablets (A A H Pharmaceuticals Ltd) |
| 33184 | Atenolol 100mg tablets (Wockhardt UK Ltd) |
| 33374 | Carvedilol 12.5mg tablets (Genus Pharmaceuticals Ltd) |
| 33569 | Oxprenolol sr 160mg Modified-release tablet (Hillcross Pharmaceuticals Ltd) |
| 33650 | Atenolol 50mg tablets (Generics (UK) Ltd) |
| 33657 | Atenolol 25mg tablets (A A H Pharmaceuticals Ltd) |
| 33850 | Atenolol 50mg tablets (Actavis UK Ltd) |
| 34171 | Labetalol 100mg Tablet (C P Pharmaceuticals Ltd) |
| 34177 | Labetalol 100mg tablets (A A H Pharmaceuticals Ltd) |
| 34188 | Labetalol 200mg Tablet (Celltech Pharma Europe Ltd) |
| 34265 | Atenolol 50mg tablets (Sandoz Ltd) |
| 34365 | Atenolol 50mg tablets (Teva UK Ltd) |
| 34443 | Atenolol 50mg tablets (Wockhardt UK Ltd) |
| 34492 | Atenolol 25mg tablets (Generics (UK) Ltd) |
| 34501 | Carvedilol 12.5mg tablets (Actavis UK Ltd) |
| 34575 | Atenolol 25mg tablets (Wockhardt UK Ltd) |
| 34585 | Atenolol 25mg tablets (Sandoz Ltd) |
| 34695 | Atenolol 50mg tablets (Kent Pharmaceuticals Ltd) |
| 34740 | Carvedilol 6.25mg tablets (Actavis UK Ltd) |
| 34741 | Carvedilol 3.125mg tablets (IVAX Pharmaceuticals UK Ltd) |
| 34754 | Atenolol 100mg tablets (Sandoz Ltd) |
| 34882 | Atenolol 50mg Tablet (Berk Pharmaceuticals Ltd) |
| 34890 | Metoprolol 50mg Tablet (Berk Pharmaceuticals Ltd) |
| 34976 | Atenolol 25mg tablets (Tillomed Laboratories Ltd) |
| 35054 | Celectol 200mg tablets (Zentiva) |
| 35062 | Trasicor 20mg tablets (Amdipharm Plc) |
| 35695 | Visken 5mg tablets (Amdipharm Plc) |
| 35778 | Labrocol 100mg Tablet (Lagap) |
| 35940 | Celectol 400mg tablets (Zentiva) |
| 36261 | Atenolol 50mg tablets (Tillomed Laboratories Ltd) |
| 37837 | Bisoprolol 2.5mg Tablet (Teva UK Ltd) |
| 38370 | Labetalol 100mg/20ml solution for injection ampoules |
| 39646 | Bisoprolol 0.625mg/5ml oral solution |
| 39819 | Esmolol 2.5g/250ml infusion bags |
| 40240 | Labetalol 400mg tablets (A A H Pharmaceuticals Ltd) |
| 40761 | Nebivolol 2.5mg tablets |
| 41740 | Celiprolol 200mg tablets (Teva UK Ltd) |
| 41827 | Labetalol 100mg tablets (Generics (UK) Ltd) |
| 42795 | Celiprolol 200mg tablets (Generics (UK) Ltd) |
| 43564 | Bisoprolol 5mg Tablet (PLIVA Pharma Ltd) |
| 44083 | Labetalol 200mg tablets (Actavis UK Ltd) |
| 44808 | Nebivolol 2.5mg tablets (A A H Pharmaceuticals Ltd) |
| 44858 | Atenolol 25mg tablets (Actavis UK Ltd) |
| 45250 | Labetalol 400mg tablets (Sandoz Ltd) |
| 45289 | Metoprolol tartrate Oral solution |
| 45309 | Acebutolol 400mg tablets (A A H Pharmaceuticals Ltd) |
| 46908 | Atenolol 100mg tablets (Kent Pharmaceuticals Ltd) |
| 46931 | Atenolol 100mg tablets (Actavis UK Ltd) |
| 46935 | Carvedilol 3.125mg tablets (Actavis UK Ltd) |
| 46936 | Carvedilol 3.125mg tablets (A A H Pharmaceuticals Ltd) |
| 47107 | Carvedilol 5mg/5ml oral suspension |
| 47300 | Nebivolol 2.5mg tablets (Glenmark Generics (Europe) Ltd) |
| 47536 | Metoprolol tartrate 12.5mg/5ml Oral suspension |
| 47673 | Labetalol 400mg Tablet (Approved Prescription Services Ltd) |
| 47674 | Labetalol 200mg Tablet (C P Pharmaceuticals Ltd) |
| 47870 | Atenolol 25mg tablets (Almus Pharmaceuticals Ltd) |
| 49142 | Carvedilol 3.125mg/5ml oral suspension |
| 49953 | Atenolol 25mg tablets (Bristol Laboratories Ltd) |
| 50403 | Bisoprolol 1.25mg Tablet (Teva UK Ltd) |
| 50702 | Atenolol 25mg tablets (Alliance Healthcare (Distribution) Ltd) |
| 51643 | Atenolol 25mg/5ml oral solution sugar free (Alliance Healthcare (Distribution) Ltd) |
| 51998 | Atenolol 25mg tablets (Co-Pharma Ltd) |
| 52310 | Atenolol 25mg tablets (Crescent Pharma Ltd) |
| 52500 | Atenolol 50mg tablets (Almus Pharmaceuticals Ltd) |
| 52728 | Beta-Adalat modified-release capsules (Lexon (UK) Ltd) |
| 53204 | Atenolol 50mg tablets (Alliance Healthcare (Distribution) Ltd) |
| 53215 | Atenolol 50mg tablets (Bristol Laboratories Ltd) |
| 53414 | Atenolol 50mg tablets (Accord Healthcare Ltd) |
| 7543 | Kalten capsules (M & A Pharmachem Ltd) |
| 9143 | Viskaldix tablets (Amdipharm Plc) |
| 12651 | Timolol 10mg / Bendroflumethiazide 2.5mg tablets |
| 14057 | Pindolol 10mg / Clopamide 5mg tablets |
| 25363 | Prestim tablets (Meda Pharmaceuticals Ltd) |
| 48745 | Generic Timolol 10mg / Amiloride 2.5mg / Hydrochlorothiazide 25mg tablets |
| 2414 | Inderal 10mg tablets (AstraZeneca UK Ltd) |
| 34804 | Propranolol 10mg tablets (Teva UK Ltd) |
| 42152 | Syprol 50mg/5ml oral solution (Rosemont Pharmaceuticals Ltd) |
| 45494 | Propranolol 10mg tablets (Almus Pharmaceuticals Ltd) |
| 707 | Propranolol 40mg tablets |
| 1572 | Sotalol 80mg tablets |
| 4004 | Sotacor 80mg tablets (Bristol-Myers Squibb Pharmaceuticals Ltd) |
| 13051 | Sotalol 200mg tablets |
| 13487 | Beta-Cardone 200mg tablets (Focus Pharmaceuticals Ltd) |
| 34371 | Sotalol 40mg tablets (A A H Pharmaceuticals Ltd) |
| 34520 | Sotalol 80mg tablets (Generics (UK) Ltd) |
| 34690 | Sotalol 80mg tablets (Sandoz Ltd) |
| 35710 | Sotalol 25mg/5ml oral suspension |
| 51492 | Sotalol 25mg/5ml oral solution |
| 3005 | Inderal LA 160mg capsules (AstraZeneca UK Ltd) |
| 594 | Bisoprolol 2.5mg tablets |
| 5713 | Bisoprolol 7.5mg tablets |
| 7091 | Bisoprolol 3.75mg tablets |
| 14058 | Cardicor 1.25mg tablets (Merck Serono Ltd) |
| 32552 | Congescor 2.5mg tablets (Tillomed Laboratories Ltd) |
| 33909 | Congescor 1.25mg tablets (Tillomed Laboratories Ltd) |
| 38991 | Bisoprolol 7.5mg tablets (A A H Pharmaceuticals Ltd) |
| 43251 | Bisoprolol 1.25mg tablets (Generics (UK) Ltd) |
| 50224 | Congescor 2.5mg tablets (Teva UK Ltd) |
| 50514 | Bisoprolol 2.5mg tablets (Chanelle Medical UK Ltd) |
| 5968 | Monocor 10mg tablets (Wyeth Pharmaceuticals) |
| 7553 | Bisoprolol 5mg/5ml oral suspension |
| 21966 | Bipranix 5mg tablets (Ashbourne Pharmaceuticals Ltd) |
| 34821 | Bisoprolol 10mg tablets (Generics (UK) Ltd) |
| 44000 | Bisoprolol 2.5mg/5ml oral suspension |
| 52611 | Bisoprolol 10mg/5ml oral solution |
| 26529 | Furosemide with penbutolol Tablet |
| 472 | Bisoprolol 5mg tablets |
| 1290 | Bisoprolol 10mg tablets |
| 4771 | Emcor LS 5mg tablets (Merck Serono Ltd) |
| 24083 | Bisoprolol 5mg tablets (Teva UK Ltd) |
| 32630 | Vivacor 10mg tablets (Lexon (UK) Ltd) |
| 34963 | Bisoprolol 5mg tablets (Actavis UK Ltd) |
| 39846 | Vivacor 5mg tablets (Lexon (UK) Ltd) |
| 3474 | Betaloc-SA 200mg tablets (AstraZeneca UK Ltd) |
| 7852 | Blocadren 10mg Tablet (Merck Sharp & Dohme Ltd) |
| 8068 | Metoprolol 200mg modified-release tablets |
| 8071 | Betaloc 50mg tablets (AstraZeneca UK Ltd) |
| 8978 | Propanix 160mg Modified-release capsule (Ashbourne Pharmaceuticals Ltd) |
| 11793 | Metoprolol 50mg/5ml oral suspension |
| 20082 | Lopresor SR 200mg tablets (Recordati Pharmaceuticals Ltd) |
| 22208 | Half propanix la 80mg Modified-release capsule (Ashbourne Pharmaceuticals Ltd) |
| 23326 | Betadur cr 160mg Modified-release capsule (Monmouth Pharmaceuticals Ltd) |
| 26228 | Propanix LA 160mg Modified-release capsule (Ashbourne Pharmaceuticals Ltd) |
| 26255 | Lopranol la 160mg Capsule (Opus Pharmaceuticals Ltd) |
| 28128 | Propranolol 80mg Modified-release capsule (Actavis UK Ltd) |
| 28788 | Half propatard la 80mg Modified-release capsule (Galen Ltd) |
| 29610 | Betim 10mg tablets (Meda Pharmaceuticals Ltd) |
| 33376 | Probeta LA 160mg Capsule (Trinity Pharmaceuticals Ltd) |
| 34094 | Metoprolol 50mg tablets (A A H Pharmaceuticals Ltd) |
| 34125 | Metoprolol 100mg tablets (A A H Pharmaceuticals Ltd) |
| 34407 | Metoprolol 50mg tablets (Teva UK Ltd) |
| 34584 | Metoprolol 50mg tablets (IVAX Pharmaceuticals UK Ltd) |
| 34884 | Propranolol 160mg Modified-release capsule (Sandoz Ltd) |
| 34945 | Propranolol 160mg Modified-release capsule (Lagap) |
| 40167 | Metoprolol 100mg tablets (IVAX Pharmaceuticals UK Ltd) |
| 40241 | Propranolol LA 160mg Capsule (Approved Prescription Services Ltd) |
| 46614 | Lopresor 50mg tablets (Recordati Pharmaceuticals Ltd) |
| 46740 | Lopresor 100mg tablets (Recordati Pharmaceuticals Ltd) |
| 51447 | Metoprolol 12.5mg/5ml oral suspension |
| 297 | Propranolol 10mg tablets |
| 25359 | Rapranol SR 160mg capsules (Ranbaxy (UK) Ltd) |
| 26229 | Beta-Prograne 160mg modified-release capsules (Tillomed Laboratories Ltd) |
| 24635 | Sotacor 10mg/ml Injection (Bristol-Myers Squibb Pharmaceuticals Ltd) |
| 3827 | Propanix 40mg Tablet (Ashbourne Pharmaceuticals Ltd) |
| 8331 | Inderal 160mg Tablet (AstraZeneca UK Ltd) |
| 12495 | Berkolol 10mg Tablet (Berk Pharmaceuticals Ltd) |
| 14552 | Propanix 10mg Tablet (Ashbourne Pharmaceuticals Ltd) |
| 21838 | Propanix 80mg Tablet (Ashbourne Pharmaceuticals Ltd) |
| 21866 | Berkolol 40mg Tablet (Berk Pharmaceuticals Ltd) |
| 24218 | Berkolol 160mg Tablet (Berk Pharmaceuticals Ltd) |
| 31833 | Angilol 80mg Tablet (DDSA Pharmaceuticals Ltd) |
| 1124 | Tenoretic 100mg/25mg tablets (AstraZeneca UK Ltd) |
| 1288 | Tenoret 50mg/12.5mg tablets (AstraZeneca UK Ltd) |
| 3526 | Amiloride with atenolol with hydrochlorothiazide capsules |
| 4983 | Atenolol with amiloride and hydrochlorothiazide capsules |
| 8623 | Prestim Tablet (ICN Pharmaceuticals France S.A.) |
| 12517 | Timolol maleate with bendroflumethiazide 20mg + 5mg Tablet |
| 13526 | Atenix Co 100 tablets (Ashbourne Pharmaceuticals Ltd) |
| 16786 | Chlortalidone 25mg with Atenolol 100mg tablets |
| 21182 | Hydrochlorothiazide with timolol and amiloride 25mg with 10mg with 2.5mg Tablet |
| 21873 | Atenix Co 50 tablets (Ashbourne Pharmaceuticals Ltd) |
| 22912 | Bendroflumethiazide 2.5mg with Propanolol 80mg capsules |
| 25730 | Timolol maleate with amiloride and hydrochlorothiazide Tablet |
| 26248 | Tenchlor 100mg/25mg tablets (Teva UK Ltd) |
| 26741 | Totaretic 50mg+12.5mg Tablet (C P Pharmaceuticals Ltd) |
| 31470 | Tenchlor 50mg/12.5mg tablets (Teva UK Ltd) |
| 33659 | Hydrochlorothiazide with metoprolol tartrate 25mg with 200mg Modified-release tablet |
| 34012 | Co-tenidone 100mg/25mg tablets (IVAX Pharmaceuticals UK Ltd) |
| 34449 | Co-tenidone 50mg/12.5mg tablets (Generics (UK) Ltd) |
| 34899 | Co-tenidone 100mg/25mg tablets (A A H Pharmaceuticals Ltd) |
| 41572 | Co-tenidone 100mg/25mg tablets (Teva UK Ltd) |
| 46952 | Co-tenidone 100mg/25mg tablets (Actavis UK Ltd) |

2.5 – Hypertension and heart failure drugs

| 599 | Bisoprolol 1.25mg tablets |
| --- | --- |
| 14030 | Cardicor 2.5mg tablets (Merck Serono Ltd) |
| 18185 | Cardicor 7.5mg tablets (Merck Serono Ltd) |
| 19853 | Cardicor 3.75mg tablets (Merck Serono Ltd) |
| 37118 | Bisoprolol 2.5mg tablets (A A H Pharmaceuticals Ltd) |
| 47041 | Bisoprolol 2.5mg tablets (Generics (UK) Ltd) |
| 50300 | Congescor 1.25mg tablets (Teva UK Ltd) |
| 51528 | Bisoprolol 1.25mg tablets (Actavis UK Ltd) |
| 52548 | Bisoprolol 1.25mg tablets (Almus Pharmaceuticals Ltd) |
| 19178 | Bisoprolol 10mg tablets (Ranbaxy (UK) Ltd) |
| 32114 | Bisoprolol 5mg tablets (Generics (UK) Ltd) |
| 33839 | Bisoprolol 10mg tablets (Actavis UK Ltd) |
| 52635 | Bisoprolol 5mg tablets (Alliance Healthcare (Distribution) Ltd) |
| 17615 | Cardicor 5mg tablets (Merck Serono Ltd) |
| 21905 | Bipranix 10mg tablets (Ashbourne Pharmaceuticals Ltd) |

2.6.2 – Calcium channel blockers

| 29 | Amlodipine besilate 5mg tablets |
| --- | --- |
| 71 | Amlodipine besilate 10mg tablets |
| 219 | Diltiazem 120mg modified-release tablets |
| 269 | Nifedipine 5mg capsules |
| 410 | Nifedipine 10mg modified-release tablets |
| 452 | Nifedipine 10mg capsules |
| 491 | Felodipine 2.5mg modified-release tablets |
| 501 | Felodipine 5mg modified-release tablets |
| 517 | Adizem sr 120mg Modified-release capsule (Napp Pharmaceuticals Ltd) |
| 536 | Tildiem la 200mg Modified-release capsule (Sanofi) |
| 541 | Adalat LA 20 tablets (Bayer Plc) |
| 568 | Felodipine 10mg modified-release tablets |
| 636 | Diltiazem 60mg modified-release capsules |
| 662 | Adalat 5mg capsules (Bayer Plc) |
| 700 | Vera-Til SR 120mg tablets (Tillomed Laboratories Ltd) |
| 729 | Amlodipine maleate 5mg tablets |
| 737 | Nifedipine 20mg modified-release capsules |
| 749 | Amlodipine 5mg tablets |
| 793 | Adizem xl 240mg Capsule (Napp Pharmaceuticals Ltd) |
| 939 | Tildiem Retard 90mg tablets (Sanofi) |
| 1118 | Verapamil 40mg tablets |
| 1120 | Verapamil 80mg tablets |
| 1130 | Viazem XL 300mg capsules (Genus Pharmaceuticals Ltd) |
| 1262 | Nifedipine 12 20mg Modified-release tablet |
| 1289 | Tildiem Retard 120mg tablets (Sanofi) |
| 1298 | Verapamil 240mg modified-release tablets |
| 1300 | Nifensar xl 20mg Modified-release tablet (Rhone-Poulenc Rorer Ltd) |
| 1449 | Nifedipine 24 30mg Modified-release tablet |
| 1529 | Posicor 50mg Tablet (Roche Products Ltd) |
| 1538 | Diltiazem 60mg tablets |
| 1574 | Verapamil 120mg modified-release capsules |
| 1686 | Diltiazem 90mg modified-release capsules |
| 1747 | Verapamil 120mg tablets |
| 1748 | Cordilox 120mg tablets (IVAX Pharmaceuticals UK Ltd) |
| 1836 | Diltiazem 60mg modified-release tablets |
| 1854 | Adalat la 30mg Tablet (Bayer Plc) |
| 1995 | Diltiazem 12hr 120mg modified-release capsules |
| 2280 | Adalat retard 10mg tablets (Bayer Plc) |
| 2343 | Adalat retard 20mg tablets (Bayer Plc) |
| 2453 | Diltiazem 60mg modified-release capsules |
| 2521 | Adalat 10mg capsules (Bayer Plc) |
| 2528 | Slozem 120mg capsules (Merck Serono Ltd) |
| 2592 | Viazem XL 120mg capsules (Genus Pharmaceuticals Ltd) |
| 2605 | Nifedipine 10mg modified-release capsules |
| 2663 | Diltiazem 240mg modified-release capsules |
| 2686 | Dilzem xl mr 240mg Modified-release capsule (Elan Pharma) |
| 2746 | Coracten SR 10mg capsules (UCB Pharma Ltd) |
| 2811 | Adizem sr 180mg Modified-release capsule (Napp Pharmaceuticals Ltd) |
| 2888 | Tildiem 60mg modified-release tablets (Sanofi) |
| 2926 | Nicardipine 20mg capsules |
| 3057 | Securon 120mg tablets (Abbott Laboratories Ltd) |
| 3061 | Diltiazem 12hr 180mg modified-release capsules |
| 3118 | Adizem sr 90mg Modified-release capsule (Napp Pharmaceuticals Ltd) |
| 3221 | Lacidipine 4mg tablets |
| 3302 | Cardene SR 30mg capsules (Astellas Pharma Ltd) |
| 3342 | Securon SR 240mg tablets (Abbott Laboratories Ltd) |
| 3343 | Half Securon SR 120mg tablets (Abbott Laboratories Ltd) |
| 3370 | Dilzem xl mr 120mg Modified-release capsule (Elan Pharma) |
| 3676 | Dilzem xl mr 180mg Modified-release capsule (Elan Pharma) |
| 3711 | Adipine MR 20 tablets (Chiesi Ltd) |
| 3712 | Coracten XL 30mg capsules (UCB Pharma Ltd) |
| 3917 | Istin 5mg tablets (Pfizer Ltd) |
| 3930 | Nifedipine 60mg modified-release tablets |
| 3931 | Posicor 100mg Tablet (Roche Products Ltd) |
| 3943 | Verapamil 240mg modified-release capsules |
| 4227 | Adalat la 60mg Tablet (Bayer Plc) |
| 4239 | Adipine MR 10 tablets (Chiesi Ltd) |
| 4308 | Dilzem sr 90mg Capsule (Elan Pharma) |
| 4408 | Slozem 240mg capsules (Merck Serono Ltd) |
| 4635 | Diltiazem 200mg modified-release capsules |
| 4732 | Diltiazem 90mg modified-release tablets |
| 4808 | Diltiazem 240mg modified-release capsules |
| 4852 | Adizem sr 120mg Modified-release tablet (Napp Pharmaceuticals Ltd) |
| 4856 | Coracten SR 20mg capsules (UCB Pharma Ltd) |
| 4923 | Diltiazem 24hr 180mg modified-release capsules |
| 4939 | Coracten XL 60mg capsules (UCB Pharma Ltd) |
| 5054 | Angitil SR 180 capsules (Chiesi Ltd) |
| 5158 | Lacidipine 2mg tablets |
| 5162 | Nifedipine 30mg modified-release capsules |
| 5181 | Angiopine MR 20mg tablets (Ashbourne Pharmaceuticals Ltd) |
| 5194 | Dilzem sr 120mg Capsule (Elan Pharma) |
| 5234 | Slozem 180mg capsules (Merck Serono Ltd) |
| 5277 | Fortipine LA 40 tablets (Mercury Pharma Group Ltd) |
| 5296 | Tildiem la 300mg Modified-release capsule (Sanofi) |
| 5326 | Diltiazem 24hr 300mg modified-release capsules |
| 5348 | Diltiazem 300mg modified-release capsules |
| 5477 | Nicardipine 30mg modified-release capsules |
| 5513 | Dilzem sr 60mg Capsule (Elan Pharma) |
| 5570 | Zanidip 10mg tablets (Recordati Pharmaceuticals Ltd) |
| 5593 | Lercanidipine 10mg tablets |
| 5806 | Tensipine MR 20 tablets (Genus Pharmaceuticals Ltd) |
| 5914 | Istin 10mg tablets (Pfizer Ltd) |
| 6309 | Adizem xl 300mg Capsule (Napp Pharmaceuticals Ltd) |
| 6477 | Amlodipine maleate 10mg tablets |
| 6510 | Univer 120mg modified-release capsules (Teva UK Ltd) |
| 6856 | Amlodipine 10mg tablets |
| 7280 | Plendil 10mg modified-release tablets (AstraZeneca UK Ltd) |
| 7398 | Viazem XL 360mg capsules (Genus Pharmaceuticals Ltd) |
| 7541 | Nifopress Retard 20mg tablets (Mercury Pharma Group Ltd) |
| 7562 | Cardene 30mg capsules (Astellas Pharma Ltd) |
| 7681 | Synadrin 60mg Tablet (Hoechst UK Ltd) |
| 8201 | Nicardipine 30mg capsules |
| 8213 | Nifedipine 24 20mg Modified-release tablet |
| 8257 | Prescal 2.5mg tablets (Novartis Pharmaceuticals UK Ltd) |
| 8310 | Isradipine 2.5mg tablets |
| 8558 | Adizem xl 120mg Capsule (Napp Pharmaceuticals Ltd) |
| 8884 | Cordilox 40mg tablets (IVAX Pharmaceuticals UK Ltd) |
| 8945 | Univer 240mg modified-release capsules (Teva UK Ltd) |
| 8975 | Verapamil 180mg modified-release capsules |
| 9240 | Adizem xl 180mg Capsule (Napp Pharmaceuticals Ltd) |
| 9269 | Nifedipine 40mg modified-release tablets |
| 9334 | Plendil 2.5mg modified-release tablets (AstraZeneca UK Ltd) |
| 9374 | Adizem 60mg Modified-release tablet (Napp Pharmaceuticals Ltd) |
| 9386 | Nicardipine 45mg modified-release capsules |
| 9410 | Angitil SR 120 capsules (Chiesi Ltd) |
| 9437 | Plendil 5mg modified-release tablets (AstraZeneca UK Ltd) |
| 9485 | Hypolar Retard 20 tablets (Sandoz Ltd) |
| 9553 | Slofedipine XL 60 tablets (Zentiva) |
| 9569 | Verapamil 120mg modified-release tablets |
| 9573 | Slofedipine XL 30mg tablets (Zentiva) |
| 9670 | Motens 4mg tablets (Boehringer Ingelheim Ltd) |
| 9708 | Diltiazem 24hr 120mg modified-release capsules |
| 9723 | Calcicard CR 90mg tablets (Teva UK Ltd) |
| 9750 | Nifedipine 60mg modified-release capsules |
| 10135 | Nifedipress mr 10mg Modified-release tablet (Sandoz Ltd) |
| 10136 | Nifedipress MR 20 tablets (Dexcel-Pharma Ltd) |
| 10153 | Felendil xl 5mg Modified-release tablet (Ratiopharm UK Ltd) |
| 10246 | Adipine XL 60mg tablets (Chiesi Ltd) |
| 10267 | Adizem-XL 200mg capsules (Napp Pharmaceuticals Ltd) |
| 10595 | Nimotop 30mg tablets (Bayer Plc) |
| 10688 | Verapamil 160mg tablets |
| 11223 | Angitil SR 90 capsules (Chiesi Ltd) |
| 11512 | Nifedipress MR 10 tablets (Dexcel-Pharma Ltd) |
| 11547 | Nimodipine 30mg tablets |
| 11769 | Calchan MR 20 tablets (Ranbaxy (UK) Ltd) |
| 11770 | Dilzem SR 60 capsules (Teva UK Ltd) |
| 11777 | Verapamil 40mg/5ml oral solution sugar free |
| 11922 | Diltiazem 60mg/5ml oral suspension |
| 11943 | Cardene 20mg capsules (Astellas Pharma Ltd) |
| 11966 | Motens 2mg tablets (Boehringer Ingelheim Ltd) |
| 11972 | Vertab SR 240 tablets (Chiesi Ltd) |
| 11973 | Calcicard CR 120mg tablets (Teva UK Ltd) |
| 12104 | Cordilox 160mg tablets (IVAX Pharmaceuticals UK Ltd) |
| 12392 | Univer 180mg modified-release capsules (Teva UK Ltd) |
| 12606 | Nifelease 20mg Modified-release tablet (Eastern Pharmaceuticals Ltd) |
| 12613 | Unipine xl 30mg Modified-release tablet (Genus Pharmaceuticals Ltd) |
| 12639 | Diltiazem HCl 90mg Modified-release tablet (Actavis UK Ltd) |
| 12705 | Angiozem CR 90mg tablets (Ashbourne Pharmaceuticals Ltd) |
| 12875 | Cardene SR 45mg capsules (Astellas Pharma Ltd) |
| 13027 | Viazem XL 240mg capsules (Genus Pharmaceuticals Ltd) |
| 13033 | Angitil XL 240 capsules (Chiesi Ltd) |
| 13075 | Dilzem XL 180 capsules (Teva UK Ltd) |
| 13127 | Dilzem XL 240 capsules (Teva UK Ltd) |
| 13139 | Adipine XL 30mg tablets (Chiesi Ltd) |
| 13240 | Dilzem XL 120 capsules (Teva UK Ltd) |
| 13243 | Lercanidipine 20mg tablets |
| 13251 | Vera-Til SR 240mg tablets (Tillomed Laboratories Ltd) |
| 13302 | Dilzem SR 90 capsules (Teva UK Ltd) |
| 13410 | Angiozem 60mg modified-release tablets (Ashbourne Pharmaceuticals Ltd) |
| 13672 | Angiopine MR 10mg tablets (Ashbourne Pharmaceuticals Ltd) |
| 13699 | Angiopine la 40mg Tablet (Ashbourne Pharmaceuticals Ltd) |
| 13856 | Verapress MR 240mg tablets (Actavis UK Ltd) |
| 13926 | Diltiazem 360mg modified-release capsules |
| 13965 | Cordilox MR 240mg tablets (Teva UK Ltd) |
| 14300 | Zanidip 20mg tablets (Recordati Pharmaceuticals Ltd) |
| 14305 | Vascalpha 10mg modified-release tablets (Actavis UK Ltd) |
| 14861 | Calchan MR 10 tablets (Ranbaxy (UK) Ltd) |
| 15221 | Dilcardia xl 180mg Modified-release capsule (Generics (UK) Ltd) |
| 15288 | Angitil XL 300 capsules (Chiesi Ltd) |
| 15652 | Mibefradil 50mg Tablet |
| 15715 | Genalat retard 20mg Modified-release tablet (Wyeth Pharmaceuticals) |
| 16038 | Dilzem SR 120 capsules (Teva UK Ltd) |
| 16073 | Nifedipress MR 10 tablets (Teva UK Ltd) |
| 16162 | Amlodipine 5mg/5ml oral suspension |
| 16328 | Verapress MR 240mg tablets (Dexcel-Pharma Ltd) |
| 16677 | Cordilox 80mg tablets (IVAX Pharmaceuticals UK Ltd) |
| 16850 | Angiozem CR 120mg tablets (Ashbourne Pharmaceuticals Ltd) |
| 17325 | Cardilate MR 10mg tablets (Teva UK Ltd) |
| 17338 | Nifedotard 20 mr 20mg Modified-release tablet (Galen Ltd) |
| 17342 | Nivaten retard 10mg Modified-release tablet (Actavis UK Ltd) |
| 17406 | Zemtard 180 XL capsules (Galen Ltd) |
| 17425 | Zemtard 120 XL capsules (Galen Ltd) |
| 17448 | Nifedipress mr 10mg Modified-release tablet (Sterwin Medicines) |
| 17492 | Zemtard 300 XL capsules (Galen Ltd) |
| 17557 | Felotens XL 5mg tablets (Genus Pharmaceuticals Ltd) |
| 17566 | Felotens XL 10mg tablets (Genus Pharmaceuticals Ltd) |
| 17586 | Slozem 300mg capsules (Merck Serono Ltd) |
| 17599 | Verapress MR 240mg tablets (Sandoz Ltd) |
| 17640 | Amlostin 5mg tablets (Discovery Pharmaceuticals Ltd) |
| 17666 | Viazem XL 180mg capsules (Genus Pharmaceuticals Ltd) |
| 18038 | Nisoldipine 20mg modified-release tablets |
| 18379 | Dilcardia SR 90mg capsules (Generics (UK) Ltd) |
| 18403 | Diltiazem HCl 180mg Modified-release capsule (Hillcross Pharmaceuticals Ltd) |
| 18404 | Diltiazem 60mg modified-release capsules (A A H Pharmaceuticals Ltd) |
| 18830 | Disogram SR 90mg capsules (Ranbaxy (UK) Ltd) |
| 18834 | Disogram SR 60mg capsules (Ranbaxy (UK) Ltd) |
| 18852 | Disogram SR 120mg capsules (Ranbaxy (UK) Ltd) |
| 18874 | Disogram SR 180mg capsules (Ranbaxy (UK) Ltd) |
| 18975 | Calcicard 60mg Tablet (3M Health Care Ltd) |
| 19013 | Clinium 120mg Tablet (LEO Pharma) |
| 19129 | Syscor MR 10 tablets (Forest Laboratories UK Ltd) |
| 19170 | Tensipine MR 10 tablets (Genus Pharmaceuticals Ltd) |
| 19175 | Verapamil 40mg tablets (IVAX Pharmaceuticals UK Ltd) |
| 19426 | Disogram SR 240mg capsules (Ranbaxy (UK) Ltd) |
| 19440 | Disogram SR 300mg capsules (Ranbaxy (UK) Ltd) |
| 19457 | Ranvera MR 240mg tablets (Ranbaxy (UK) Ltd) |
| 19459 | Verapamil 240mg modified-release tablets (A A H Pharmaceuticals Ltd) |
| 20257 | Cardilate MR 20mg tablets (IVAX Pharmaceuticals UK Ltd) |
| 20311 | Nifedipress mr 20mg Modified-release tablet (Generics (UK) Ltd) |
| 20459 | Felendil xl 10mg Modified-release tablet (Ratiopharm UK Ltd) |
| 20591 | Nifedipress MR 20 tablets (Teva UK Ltd) |
| 20642 | Bi-carzem sr 60mg Modified-release capsule (Tillomed Laboratories Ltd) |
| 20878 | Angiopine 10 capsules (Ashbourne Pharmaceuticals Ltd) |
| 20890 | Zemtard 240 XL capsules (Galen Ltd) |
| 21145 | Dilcardia SR 60mg capsules (Generics (UK) Ltd) |
| 21216 | Hypolar Retard 10mg tablets (Sandoz Ltd) |
| 21245 | Nifedipress mr 10mg Modified-release tablet (Actavis UK Ltd) |
| 21763 | Diltiazem 60mg modified-release tablets (A A H Pharmaceuticals Ltd) |
| 21773 | Diltiazem HCl 60mg Tablet (Generics (UK) Ltd) |
| 21778 | Diltiazem 60mg modified-release tablets (Teva UK Ltd) |
| 21795 | Retalzem 60 modified-release tablets (Kent Pharmaceuticals Ltd) |
| 21872 | Angiopine 5mg Capsule (Ashbourne Pharmaceuticals Ltd) |
| 21886 | Nifedipress MR 20 tablets (Actavis UK Ltd) |
| 21918 | Optil 60mg modified-release tablets (Opus Pharmaceuticals Ltd) |
| 22019 | Calanif 10mg Capsule (Berk Pharmaceuticals Ltd) |
| 22142 | Calcilat 10mg Capsule (Eastern Pharmaceuticals Ltd) |
| 22217 | Nimodrel 10mg modified-release tablet (Opus Pharmaceuticals Ltd) |
| 22241 | Mibefradil 100mg Tablet |
| 22619 | Britiazim 60mg Modified-release tablet (Thames Laboratories Ltd) |
| 22696 | Slofedipine 20mg tablets (Sterwin Medicines) |
| 23233 | Bi-carzem sr 90mg Modified-release capsule (Tillomed Laboratories Ltd) |
| 23733 | Optil sr 90mg Modified-release capsule (Opus Pharmaceuticals Ltd) |
| 23736 | Hypolar XL 30 tablets (Sandoz Ltd) |
| 23805 | Nisoldipine 10mg modified-release tablets |
| 23823 | Nisoldipine 30mg modified-release tablets |
| 24228 | Nimodrel 20mg modified-release tablet (Opus Pharmaceuticals Ltd) |
| 24365 | Cardioplen XL 5mg tablets (Chiesi Ltd) |
| 24366 | Cardioplen XL 10mg tablets (Chiesi Ltd) |
| 25132 | Nifopress MR 20mg tablets (Teva UK Ltd) |
| 25572 | Felogen XL 5mg tablets (Generics (UK) Ltd) |
| 25646 | Nivaten retard 20mg Modified-release tablet (Actavis UK Ltd) |
| 25777 | Dilcardia SR 120mg capsules (Generics (UK) Ltd) |
| 25919 | Nifedipine 20mg modified-release tablets (A A H Pharmaceuticals Ltd) |
| 26265 | Calanif 5mg Capsule (Berk Pharmaceuticals Ltd) |
| 26267 | Optil sr 120mg Modified-release capsule (Opus Pharmaceuticals Ltd) |
| 26269 | Optil sr 180mg Modified-release capsule (Opus Pharmaceuticals Ltd) |
| 26270 | Optil xl 300mg Modified-release capsule (Opus Pharmaceuticals Ltd) |
| 26309 | Optil xl 240mg Modified-release capsule (Opus Pharmaceuticals Ltd) |
| 26337 | Cabren 10mg modified-release tablets (Teva UK Ltd) |
| 26460 | Dilcardia xl 240mg Modified-release capsule (Generics (UK) Ltd) |
| 26463 | Zemret xl 240mg Capsule (Neo Laboratories Ltd) |
| 26674 | Verapamil 5mg/2ml solution for injection ampoules |
| 26759 | Zildil SR 60mg capsules (Chanelle Medical UK Ltd) |
| 26774 | Nifedipine 10mg/5ml Oral suspension |
| 27135 | Diltiazem sr 90mg Capsule (Hillcross Pharmaceuticals Ltd) |
| 27136 | Diltiazem 90mg modified-release tablets (A A H Pharmaceuticals Ltd) |
| 27295 | Securon IV 5mg/2ml solution for injection ampoules (Abbott Laboratories Ltd) |
| 27401 | Kenzem SR 90mg capsules (Kent Pharmaceuticals Ltd) |
| 27685 | Diltiazem HCl 300mg Capsule (PLIVA Pharma Ltd) |
| 28688 | Nifedipine 10mg modified-release tablets (A A H Pharmaceuticals Ltd) |
| 28721 | Neofel XL 5mg tablets (Fannin UK Ltd) |
| 28949 | Bi-carzem sr 120mg Modified-release capsule (Tillomed Laboratories Ltd) |
| 29044 | Neofel XL 10mg tablets (Fannin UK Ltd) |
| 29145 | Felendil xl 2.5mg Modified-release tablet (Ratiopharm UK Ltd) |
| 29637 | Verapress MR 240mg tablets (Teva UK Ltd) |
| 29676 | Calazem 60mg Modified-release tablet (Berk Pharmaceuticals Ltd) |
| 30197 | Diltiazem 120mg modified-release capsules |
| 30199 | Nifedipine 30mg modified-release tablets |
| 30242 | Diltiazem 180mg modified-release capsules |
| 30462 | Ethimil MR 240mg tablets (Genus Pharmaceuticals Ltd) |
| 30473 | Coroday MR 20mg tablets (Generics (UK) Ltd) |
| 30557 | Felogen XL 10mg tablets (Generics (UK) Ltd) |
| 30758 | Lidoflazine 120mg Tablet |
| 30915 | Cabren 2.5mg modified-release tablets (Teva UK Ltd) |
| 30991 | Cabren 5mg modified-release tablets (Teva UK Ltd) |
| 31336 | Syscor MR 30 tablets (Forest Laboratories UK Ltd) |
| 31337 | Syscor MR 20 tablets (Forest Laboratories UK Ltd) |
| 31489 | Bi-carzem xl 240mg Capsule (Tillomed Laboratories Ltd) |
| 31490 | Zolvera 40mg/5ml oral solution (Rosemont Pharmaceuticals Ltd) |
| 31676 | Diltiazem HCl 120mg Modified-release tablet (Actavis UK Ltd) |
| 31711 | Verapamil 80mg tablets (A A H Pharmaceuticals Ltd) |
| 31737 | Zildil SR 120mg capsules (Chanelle Medical UK Ltd) |
| 31761 | Amlostin 10mg tablets (Discovery Pharmaceuticals Ltd) |
| 32089 | Diltiazem HCl 120mg Modified-release capsule (Hillcross Pharmaceuticals Ltd) |
| 32262 | Diltiazem HCl 60mg Tablet (C P Pharmaceuticals Ltd) |
| 32590 | Verapamil 40mg tablets (Generics (UK) Ltd) |
| 32595 | Amlodipine 5mg tablets (A A H Pharmaceuticals Ltd) |
| 32658 | Dilcardia xl 120mg Modified-release capsule (Generics (UK) Ltd) |
| 32870 | Diltiazem 60mg modified-release tablets (Sterwin Medicines) |
| 32917 | Amlodipine 5mg tablets (IVAX Pharmaceuticals UK Ltd) |
| 32922 | Felodipine 10mg Modified-release tablet (Sandoz Ltd) |
| 33025 | Nimodrel XL 30mg tablets (Zurich Pharmaceuticals) |
| 33091 | Felodipine 10mg modified-release tablets (A A H Pharmaceuticals Ltd) |
| 33471 | Verapamil 40mg tablets (Actavis UK Ltd) |
| 33932 | Parmid XL 5mg tablets (Sandoz Ltd) |
| 34093 | Amlodipine 10mg tablets (A A H Pharmaceuticals Ltd) |
| 34101 | Nifedipine mr 20mg Modified-release tablet (IVAX Pharmaceuticals UK Ltd) |
| 34115 | Nifedipine 60mg Modified-release tablet |
| 34146 | Nifedipine mr 10mg Modified-release tablet (IVAX Pharmaceuticals UK Ltd) |
| 34187 | Nifedipine 10mg Modified-release tablet (Generics (UK) Ltd) |
| 34247 | Nifedipine 10mg Capsule (Berk Pharmaceuticals Ltd) |
| 34377 | Diltiazem HCl 90mg Modified-release capsule (Hillcross Pharmaceuticals Ltd) |
| 34475 | Diltiazem HCl 90mg Modified-release tablet (IVAX Pharmaceuticals UK Ltd) |
| 34522 | Nifedipine 5mg capsules (A A H Pharmaceuticals Ltd) |
| 34581 | Diltiazem HCl 60mg Modified-release tablet (Kent Pharmaceuticals Ltd) |
| 34607 | Nifedipine 5mg capsules (IVAX Pharmaceuticals UK Ltd) |
| 34824 | Diltiazem HCl 120mg Modified-release tablet (IVAX Pharmaceuticals UK Ltd) |
| 34959 | Verapamil 40mg tablets (A A H Pharmaceuticals Ltd) |
| 34975 | Nifedipine 5mg capsules (Teva UK Ltd) |
| 35084 | Vascalpha 5mg modified-release tablets (Actavis UK Ltd) |
| 35096 | Exforge 10mg/160mg tablets (Novartis Pharmaceuticals UK Ltd) |
| 35189 | Amlodipine 10mg / Valsartan 160mg tablets |
| 35317 | Exforge 5mg/80mg tablets (Novartis Pharmaceuticals UK Ltd) |
| 35329 | Amlodipine 5mg / Valsartan 80mg tablets |
| 35343 | Amlodipine 5mg / Valsartan 160mg tablets |
| 35592 | Cardioplen XL 2.5mg tablets (Chiesi Ltd) |
| 35646 | Neozipine XL 60mg tablets (Fannin UK Ltd) |
| 35696 | Kenzem SR 120mg capsules (Kent Pharmaceuticals Ltd) |
| 35697 | Exforge 5mg/160mg tablets (Novartis Pharmaceuticals UK Ltd) |
| 35729 | Verapamil 80mg tablets (Teva UK Ltd) |
| 36202 | Amlodipine 10mg tablets (Actavis UK Ltd) |
| 36583 | Zemret xl 180mg Capsule (Neo Laboratories Ltd) |
| 36620 | Parmid XL 10mg tablets (Sandoz Ltd) |
| 36664 | Zemret xl 300mg Capsule (Neo Laboratories Ltd) |
| 37025 | Nifedipine 20mg modified-release tablets |
| 37184 | Valni XL 30mg tablets (Zentiva) |
| 37530 | Neozipine XL 30mg tablets (Fannin UK Ltd) |
| 37726 | Nifedipine 100mg/5ml oral suspension |
| 37774 | Kenzem SR 60mg capsules (Kent Pharmaceuticals Ltd) |
| 37897 | Felotens XL 2.5mg tablets (Genus Pharmaceuticals Ltd) |
| 38066 | Diltiazem HCl 60mg Modified-release tablet (Lagap) |
| 38107 | Nifedipine sr 30mg Tablet (Hillcross Pharmaceuticals Ltd) |
| 38434 | Keloc SR 10mg tablets (Teva UK Ltd) |
| 38545 | Tildiem LA 200 capsules (Sanofi) |
| 38632 | Adizem-SR 90mg capsules (Napp Pharmaceuticals Ltd) |
| 38634 | Adizem-XL 300mg capsules (Napp Pharmaceuticals Ltd) |
| 38818 | Adizem-SR 120mg capsules (Napp Pharmaceuticals Ltd) |
| 38831 | Adizem-SR 180mg capsules (Napp Pharmaceuticals Ltd) |
| 38855 | Adizem-XL 180mg capsules (Napp Pharmaceuticals Ltd) |
| 38865 | Adizem-XL 120mg capsules (Napp Pharmaceuticals Ltd) |
| 38876 | Tildiem LA 300 capsules (Sanofi) |
| 38882 | Adizem-XL 240mg capsules (Napp Pharmaceuticals Ltd) |
| 38964 | Adizem-SR 120mg tablets (Napp Pharmaceuticals Ltd) |
| 39009 | Verapamil 40mg tablets (Teva UK Ltd) |
| 39171 | Bi-Carzem SR 60mg capsules (Tillomed Laboratories Ltd) |
| 39298 | Bi-Carzem SR 90mg capsules (Tillomed Laboratories Ltd) |
| 39357 | Neofel XL 2.5mg tablets (Fannin UK Ltd) |
| 39800 | Valni XL 60mg tablets (Zentiva) |
| 39804 | Amlodipine 5mg tablets (Dr Reddy's Laboratories (UK) Ltd) |
| 39914 | Amlodipine 5mg tablets (Teva UK Ltd) |
| 40074 | Nifedipine 20mg Capsule |
| 40405 | Verapamil 120mg tablets (Teva UK Ltd) |
| 40633 | Vascalpha 5mg modified-release tablets (Almus Pharmaceuticals Ltd) |
| 41489 | Bi-Carzem SR 120mg capsules (Tillomed Laboratories Ltd) |
| 41586 | Verapamil 80mg tablets (Actavis UK Ltd) |
| 41635 | Diltiazem 60mg modified-release tablets (IVAX Pharmaceuticals UK Ltd) |
| 41679 | Verapamil 80mg tablets (IVAX Pharmaceuticals UK Ltd) |
| 41693 | Verapamil 120mg tablets (Generics (UK) Ltd) |
| 41979 | Adipine la 30mg Modified-release tablet (Chiesi Ltd) |
| 42210 | Amlodipine 10mg tablets (Zentiva) |
| 42625 | Vera-Til SR 120mg tablets (Actavis UK Ltd) |
| 42731 | Diltiazem sr 120mg Capsule (Hillcross Pharmaceuticals Ltd) |
| 42804 | Diltiazem HCl 180mg Capsule (PLIVA Pharma Ltd) |
| 42819 | Diltiazem xl 240mg Capsule (Hillcross Pharmaceuticals Ltd) |
| 42912 | Nifedipine 10mg capsules (Teva UK Ltd) |
| 43222 | Valni 20 Retard tablets (Tillomed Laboratories Ltd) |
| 43394 | Pinefeld XL 10mg tablets (Tillomed Laboratories Ltd) |
| 43410 | Nifedipine extra 60mg Modified-release tablet |
| 43430 | Diltiazem 120mg modified-release tablets (A A H Pharmaceuticals Ltd) |
| 43470 | Amlodipine 5mg tablets (Wockhardt UK Ltd) |
| 43511 | Nifedipine 10mg capsules (A A H Pharmaceuticals Ltd) |
| 43512 | Felodipine 5mg modified-release tablets (A A H Pharmaceuticals Ltd) |
| 43515 | Nifedipine 10mg capsules (Actavis UK Ltd) |
| 43753 | Adalat LA 30 tablets (Bayer Plc) |
| 43790 | Vascalpha 10mg modified-release tablets (Almus Pharmaceuticals Ltd) |
| 43818 | Adalat LA 60 tablets (Bayer Plc) |
| 43879 | Vera-Til SR 240mg tablets (Actavis UK Ltd) |
| 43880 | Amlodipine 5mg tablets (Almus Pharmaceuticals Ltd) |
| 44192 | Zemret 240 XL capsules (Tillomed Laboratories Ltd) |
| 44859 | Felodipine sr 5mg Tablet (Approved Prescription Services Ltd) |
| 44887 | Bi-carzem xl 300mg Capsule (Tillomed Laboratories Ltd) |
| 45070 | Amlodipine 10mg/5ml oral suspension |
| 45279 | Amlodipine 5mg tablets (Sandoz Ltd) |
| 45292 | Nicardipine 30mg capsules (A A H Pharmaceuticals Ltd) |
| 45308 | Verapamil 240mg modified-release tablets (Generics (UK) Ltd) |
| 45564 | Diltiazem 2% ointment |
| 45685 | Adanif XL 30mg tablets (Focus Pharmaceuticals Ltd) |
| 45759 | Diltiazem HCl 240mg Capsule (PLIVA Pharma Ltd) |
| 46009 | Verapamil 120mg tablets (Kent Pharmaceuticals Ltd) |
| 46233 | Amlodipine Oral solution |
| 46445 | Nifedipine 10mg capsules (IVAX Pharmaceuticals UK Ltd) |
| 46724 | Amlodipine 5mg/5ml oral solution |
| 46887 | Adanif XL 60mg tablets (Focus Pharmaceuticals Ltd) |
| 46937 | Diltiazem 60mg modified-release tablets (Actavis UK Ltd) |
| 46955 | Verapamil 80mg tablets (Generics (UK) Ltd) |
| 47002 | Amlodipine 10mg/5ml sugar free Oral suspension |
| 47027 | Nifedipine 10mg Modified-release tablet (Kent Pharmaceuticals Ltd) |
| 47217 | Adipine la 60mg Modified-release tablet (Chiesi Ltd) |
| 47222 | Verapamil 120mg modified-release tablets (A A H Pharmaceuticals Ltd) |
| 47230 | Verapamil 240mg modified-release tablets (Teva UK Ltd) |
| 47285 | Nifedipine xl 60mg Tablet (Hillcross Pharmaceuticals Ltd) |
| 47331 | Lercanidipine 10mg tablets (Generics (UK) Ltd) |
| 47415 | Diltiazem sr 60mg Capsule (Hillcross Pharmaceuticals Ltd) |
| 47529 | Nifedipine 20mg/ml oral drops |
| 47530 | Horizem SR 60mg capsules (Horizon lifecare) |
| 47608 | Zemret 300 XL capsules (Tillomed Laboratories Ltd) |
| 47614 | Nifedipine 30mg modified-release tablets (A A H Pharmaceuticals Ltd) |
| 47707 | Nifedipine Oral solution |
| 47724 | Bi-Carzem XL 240mg capsules (Tillomed Laboratories Ltd) |
| 47732 | Zemret 180 XL capsules (Tillomed Laboratories Ltd) |
| 47887 | Nimodrel XL 60mg tablets (Zurich Pharmaceuticals) |
| 47996 | Diltiazem 2% gel |
| 48009 | Felodipine 5mg Modified-release tablet (Sandoz Ltd) |
| 48272 | Diltiazem 60mg modified-release capsules (Alliance Healthcare (Distribution) Ltd) |
| 48282 | Diltiazem 90mg modified-release capsules (A A H Pharmaceuticals Ltd) |
| 48288 | Diltiazem 120mg modified-release capsules (A A H Pharmaceuticals Ltd) |
| 48457 | Diltiazem 90mg modified-release capsules (Alliance Healthcare (Distribution) Ltd) |
| 48870 | Adizem-SR 90mg capsules (Doncaster Pharmaceuticals Ltd) |
| 49001 | Diltiazem 120mg modified-release tablets (Alliance Healthcare (Distribution) Ltd) |
| 49289 | Diltiazem 120mg modified-release capsules (Alliance Healthcare (Distribution) Ltd) |
| 49338 | Nifedipine 20mg modified-release tablets (Alliance Healthcare (Distribution) Ltd) |
| 49390 | Diltiazem 90mg modified-release tablets (Alliance Healthcare (Distribution) Ltd) |
| 49500 | Diltiazem 2% ointment (Drug Tariff Special Order) |
| 49636 | Amlodipine 10mg tablets (Doncaster Pharmaceuticals Ltd) |
| 49762 | Nifedipine 10mg modified-release tablets (Alliance Healthcare (Distribution) Ltd) |
| 51261 | Tildiem Retard 120mg tablets (Mawdsley-Brooks & Company Ltd) |
| 51461 | Securon SR 240mg tablets (Waymade Healthcare Plc) |
| 51917 | Adalat LA 60 tablets (Sigma Pharmaceuticals Plc) |
| 52017 | Adalat LA 30 tablets (Mawdsley-Brooks & Company Ltd) |
| 52276 | Adizem-XL 180mg capsules (Doncaster Pharmaceuticals Ltd) |
| 52440 | Amlodipine 10mg/5ml oral solution |
| 52701 | Tildiem LA 200 capsules (Mawdsley-Brooks & Company Ltd) |
| 53278 | Adalat LA 30 tablets (Necessity Supplies Ltd) |
| 53357 | Nifedipine 10mg/5ml oral suspension |
| 53500 | Adalat LA 30 tablets (Doncaster Pharmaceuticals Ltd) |
| 53629 | Adalat retard 20mg tablets (Lexon (UK) Ltd) |
| 18223 | Trandolapril with verapamil 2mg + 180mg Modified-release capsule |
| 40316 | Olmesartan medoxomil 20mg / Amlodipine 5mg tablets |
| 41203 | Sevikar 40mg/10mg tablets (Daiichi Sankyo UK Ltd) |
| 15117 | Nifedipine with atenolol 20mg + 50mg Capsule |
| 8524 | Securon 40mg Tablet (Abbott Laboratories Ltd) |
| 10832 | Securon 80mg Tablet (Abbott Laboratories Ltd) |
| 22826 | Securon 160mg Tablet (Abbott Laboratories Ltd) |
| 26252 | Berkatens 160mg Tablet (Berk Pharmaceuticals Ltd) |
| 28843 | Verapamil hc 80mg Tablet (Celltech Pharma Europe Ltd) |
| 28844 | Berkatens 120mg Tablet (Berk Pharmaceuticals Ltd) |
| 45051 | Verapamil hc 240mg Modified-release tablet (Actavis UK Ltd) |
| 46884 | Verapamil hc 240mg Modified-release tablet (Sandoz Ltd) |
| 18606 | Diltiazem and hydrochlorothiazide 150mg+12.5mg modified-release capsules |
