# Supplementary material for: Elevated blood pressure and risk of mitral regurgitation: A longitudinal cohort study of 5.5 million United Kingdom adults
Source: PLoS Med. 2017 Oct 17;14(10):e1002404. doi: 10.1371/journal.pmed.1002404 (PMC5644976; doi:10.1371/journal.pmed.1002404)

### S1 Fig. Log cumulative hazards plots for systolic blood pressure relating to mitral regurgitation


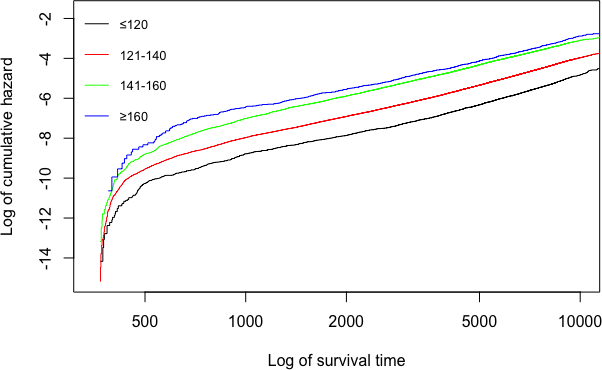

Supplement: S1 Fig — Abbreviation: SBP, systolic blood pressure. (DOCX) [file pmed.1002404.s003.docx]
