# Supplementary material for: Elevated blood pressure and risk of mitral regurgitation: A longitudinal cohort study of 5.5 million United Kingdom adults
Source: PLoS Med. 2017 Oct 17;14(10):e1002404. doi: 10.1371/journal.pmed.1002404 (PMC5644976; doi:10.1371/journal.pmed.1002404)
Supplement: S2 Fig — Abbreviations: DBP, diastolic blood pressure; HR, hazard ratio; PP, pulse pressure. (DOCX) [file pmed.1002404.s004.docx]

### **S2 Fig.** Hazard ratios for mitral regurgitation per 10 mmHg higher usual diastolic blood pressure or per 15 mmHg increase in usual pulse pressure, by age categories.

Hazard ratios (HR) and 95% confidence intervals (CI) are displayed using floating absolute risks and corrected for regression dilution. Models are adjusted for age, sex, BMI, smoking, calendar year, total cholesterol, LDL and HDL cholesterol. MR = mitral regurgitation; DBP = diastolic blood pressure; PP = pulse pressure

_
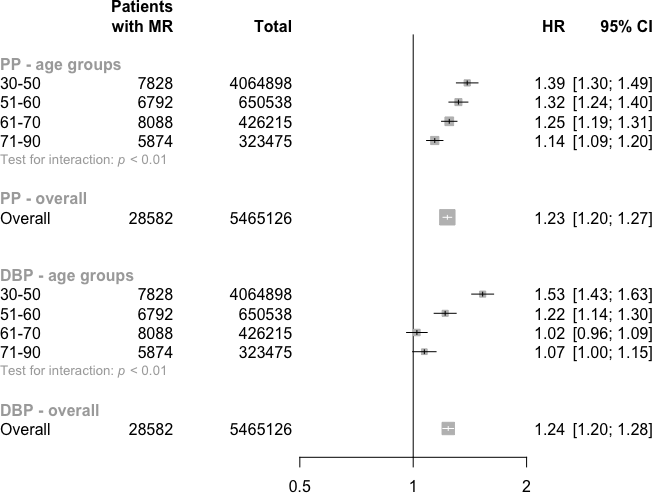
_
