# Supplementary material for: Elevated blood pressure and risk of mitral regurgitation: A longitudinal cohort study of 5.5 million United Kingdom adults
Source: PLoS Med. 2017 Oct 17;14(10):e1002404. doi: 10.1371/journal.pmed.1002404 (PMC5644976; doi:10.1371/journal.pmed.1002404)

### **S4 Fig.** Adjusted age-specific hazard ratios of systolic blood pressure for mitral stenosis

Adjustments were for sex, BMI, smoking, total cholesterol, LDL, HDL. Confidence intervals are displayed as floating absolute risks. The area of each square is proportional to the inverse variance of the estimate. Hazard ratios (HRs) and 95% confidence intervals (CI) for each category are displayed relative to the reference category (individuals aged 30-50 years with usual systolic blood pressure 115-120 mmHg).


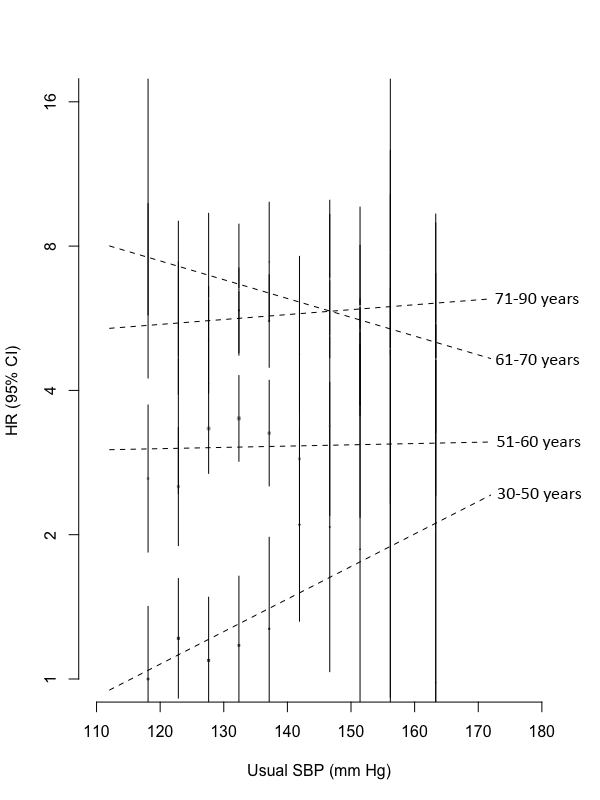

Supplement: S4 Fig — Abbreviations: HR, hazard ratio; SBP, systolic blood pressure. (DOCX) [file pmed.1002404.s006.docx]
