# Supplementary material for: Elevated blood pressure and risk of mitral regurgitation: A longitudinal cohort study of 5.5 million United Kingdom adults
Source: PLoS Med. 2017 Oct 17;14(10):e1002404. doi: 10.1371/journal.pmed.1002404 (PMC5644976; doi:10.1371/journal.pmed.1002404)

### **S5 Fig.** Hazard ratios for mitral stenosis by categories of usual systolic blood pressure.

Hazard ratios (HR) and 95% confidence intervals (CI) are displayed using floating absolute risk and corrected for regression dilution. Models are adjusted for age, sex, BMI, calendar year, smoking, total cholesterol, LDL and HDL. MS = mitral stenosis; SBP = systolic blood pressure


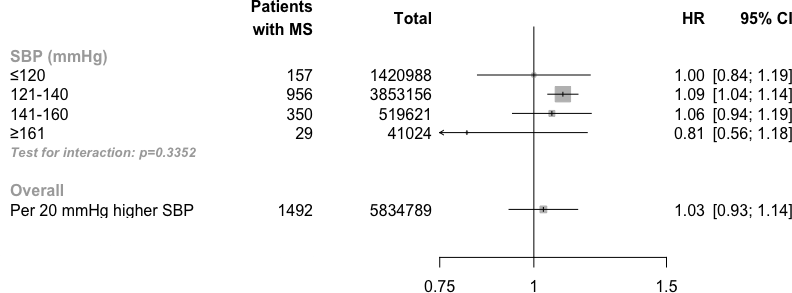

Supplement: S5 Fig — Abbreviations: HR, hazard ratio; SBP, systolic blood pressure. (DOCX) [file pmed.1002404.s007.docx]
