# Supplementary material for: Elevated blood pressure and risk of mitral regurgitation: A longitudinal cohort study of 5.5 million United Kingdom adults
Source: PLoS Med. 2017 Oct 17;14(10):e1002404. doi: 10.1371/journal.pmed.1002404 (PMC5644976; doi:10.1371/journal.pmed.1002404)

### **S6 Fig.** Hazard ratios per 20 mmHg higher usual systolic blood pressure for mitral stenosis, with progressive adjustment for age, sex, calendar year, BMI, smoking and baseline LDL, HDL, and total cholesterol, and stratified by practice level.


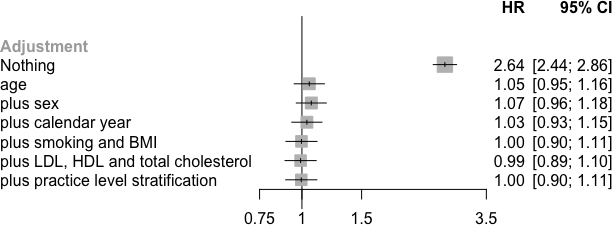

Supplement: S6 Fig — Abbreviations: BMI, body mass index; HDL, high-density lipoprotein; HR, hazard ratio; LDL, low-density lipoprotein; SBP, systolic blood pressure. (DOCX) [file pmed.1002404.s008.docx]
