# Supplementary material for: Elevated blood pressure and risk of mitral regurgitation: A longitudinal cohort study of 5.5 million United Kingdom adults
Source: PLoS Med. 2017 Oct 17;14(10):e1002404. doi: 10.1371/journal.pmed.1002404 (PMC5644976; doi:10.1371/journal.pmed.1002404)
Supplement: S1 Table — (DOCX) [file pmed.1002404.s009.docx]

### **S1 Table**. Read and ICD-10 codes for mitral valve disease.

| Read code | Description | Regurgitation | Stenosis | Replacement |
| --- | --- | --- | --- | --- |
| G540.16 | Mitral regurgitation | 1 |  |  |
| G110.00 | Mitral stenosis |  | 1 |  |
| G540.00 | Mitral valve incompetence | 1 |  |  |
| 7910300 | Replacement of mitral valve NEC |  |  | 1 |
| 7910.12 | Replacement of mitral valve |  |  | 1 |
| 7N40000 | [SO]Mitral valve |  |  | 1 |
| 7910 | Plastic repair of mitral valve |  |  | 1 |
| 7918000 | Annuloplasty of mitral valve |  |  | 1 |
| 7915000 | Revision of plastic repair of mitral valve |  |  | 1 |
| 7916000 | Open mitral valvotomy |  |  | 1 |
| 7917000 | Closed mitral valvotomy |  |  | 1 |
| 7910200 | Prosthetic replacement of mitral valve |  |  | 1 |
| G111.11 | Mitral incompetence - rheumatic | 1 |  |  |
| 7910400 | Mitral valvuloplasty NEC |  |  | 1 |
| G111.12 | Mitral regurgitation - rheumatic | 1 |  |  |
| G113.00 | Nonrheumatic mitral valve stenosis |  | 1 |  |
| 7910211 | Bjork-Shiley prosthetic replacement of mitral valve |  |  | 1 |
| G110.11 | Rheumatic mitral stenosis |  | 1 |  |
| G540100 | Mitral incompetence, cause unspecified | 1 |  |  |
| 7910.11 | Mitral valvuloplasty |  |  | 1 |
| G540.12 | Mitral valve insufficiency | 1 |  |  |
| 7910z00 | Plastic repair of mitral valve NOS |  |  | 1 |
| G112.13 | Mitral stenosis with regurgitation |  | 1 |  |
| 7910213 | Carpentier prosthetic replacement of mitral valve |  |  | 1 |
| 7910100 | Xenograft replacement of mitral valve |  |  | 1 |
| G111.00 | Rheumatic mitral insufficiency | 1 |  |  |
| 7910212 | Bjork-Shiley prosthetic replacement of mitral valve |  |  | 1 |
| G114.00 | Ruptured mitral valve cusp |  |  |  |
| 7910y00 | Other specified plastic repair of mitral valve |  |  | 1 |
| 7910214 | Edwards prosthetic replacement of mitral valve |  |  | 1 |
| 7910411 | Mitral valve repair NEC |  |  | 1 |
| 7910000 | Allograft replacement of mitral valve |  |  | 1 |
| 791B200 | Operations on mitral subvalvar apparatus |  |  | 1 |
| G540.14 | Mitral valve regurgitation | 1 |  |  |
| G540000 | Mitral incompetence, non-rheumatic | 1 |  |  |
