# Supplementary material for: Elevated blood pressure and risk of mitral regurgitation: A longitudinal cohort study of 5.5 million United Kingdom adults
Source: PLoS Med. 2017 Oct 17;14(10):e1002404. doi: 10.1371/journal.pmed.1002404 (PMC5644976; doi:10.1371/journal.pmed.1002404)
Supplement: S2 Table — Abbreviation: BP, blood pressure. (DOCX) [file pmed.1002404.s010.docx]

### **S2 Table**. Relative ability of different blood pressure indices to independently predict mitral regurgitation, by age categories and overall.

The concordance of Cox proportional hazard model relating age-specific hazard ratios to baseline blood pressure indices (i.e., without regression dilution correction) with their standard errors (shown in brackets). Effects of each blood pressure index are adjusted for age, sex, BMI, smoking, calendar year, total cholesterol, LDL, HDL and practice-level index of multiple deprivation.

| Blood pressure at baseline | Age at baseline | | | | |
| --- | --- | --- | --- | --- | --- |
|  | 30-50 | 51-60 | 61-70 | 71-90 | Overall |
| Systolic blood pressure | 0.57 (0.0042) | 0.54 (0.0039) | 0.52 (0.0035) | 0.51 (0.0042) | 0.68 (0.0019) |
| Diastolic blood pressure | 0.56 (0.0041) | 0.52 (0.0039) | 0.50 (0.0035) | 0.51 (0.0042) | 0.61 (0.0019) |
| Pulse pressure | 0.55 (0.0041) | 0.54 (0.0039) | 0.53 (0.0035) | 0.51 (0.0042) | 0.67 (0.0019) |
| Mean arterial pressure | 0.57 (0.0042) | 0.53 (0.0039) | 0.51 (0.0035) | 0.50 (0.0042) | 0.66 (0.0019) |
| Mid blood pressure | 0.57 (0.0042) | 0.54 (0.0039) | 0.51 (0.0035) | 0.50 (0.0042) | 0.67 (0.0019) |
